# Medium Composition Determines the Dynamics of Boar In Vitro Sperm Capacitation-Associated Events

**DOI:** 10.3390/ijms27104567

**Published:** 2026-05-19

**Authors:** Barbora Klusackova, Zuzana Pilsova, Barbora Bryndova, Aneta Pilsova, Natalie Zelenkova, Petr Pecina, Michal Knezu, Petra Secova, Pavla Tymich Hegrova, Eva Chmelikova, Katerina Komrskova, Ondrej Simonik, Pavla Postlerova

**Affiliations:** 1 Department of Veterinary Sciences, Faculty of Agrobiology, Food, and Natural Resources, Czech University of Life Sciences Prague, 165 00 Prague, Czech Republic; klusackovab@af.czu.cz (B.K.); pilsovaz@af.czu.cz (Z.P.); pilsova@af.czu.cz (A.P.); zelenkovan@af.czu.cz (N.Z.); pokornapavla@af.czu.cz (P.T.H.); chmelikova@af.czu.cz (E.C.); postlerova@af.czu.cz (P.P.); 2 Laboratory of Reproductive Biology, Institute of Biotechnology of the Czech Academy of Sciences, BIOCEV, 252 50 Vestec, Czech Republic; barbora.bryndova@natur.cuni.cz (B.B.); katerina.komrskova@ibt.cas.cz (K.K.); ondrej.simonik@ibt.cas.cz (O.S.);; 3 Department of Biochemistry, Faculty of Science, Charles University, 128 00 Prague, Czech Republic; 4 Laboratory of Bioenergetics, Institute of Physiology, Czech Academy of Sciences, 142 00 Prague, Czech Republic; petr.pecina@fgu.cas.cz (P.P.); michal.knezu@fgu.cas.cz (M.K.); 5 Department of Cell Biology, Faculty of Science, Charles University, 128 00 Prague, Czech Republic; 6 Laboratory of Reproductive Physiology, Institute of Animal Biochemistry and Genetics, Centre of Biosciences, Slovak Academy of Sciences, 841 04 Bratislava, Slovakia; petra.secova@savba.sk; 7 Department of Zoology, Faculty of Science, Charles University, 128 00 Prague, Czech Republic

**Keywords:** phosphotyrosine, phosphorylation, protein kinase A, motility, oxidative phosphorylation, reproduction

## Abstract

Capacitation is a key maturation process that enables spermatozoa to acquire fertilizing ability and can be induced in vitro using capacitation media. Because capacitation protocols differ markedly among laboratories, we compared three compositionally distinct Hepes-, Tris-, and TALP-based media. This study was performed in boar spermatozoa using 3–6 biological replicates of pooled ejaculates depending on the assay, with 46 ejaculate samples from 12 boars in total. The aim was to determine whether such non-standardized conditions differentially affect signaling pathways leading to capacitation and thereby influence the detection of commonly used capacitation markers. We found clear differences among the tested media. All three induced capacitation-associated events, but their functional and molecular effects were not equivalent. The Hepes-based medium supported sperm motility most effectively, increasing total and progressive motility to 60.0% and 48.7%, respectively, after 1 h of incubation and maintaining the highest motility throughout the incubation period. In contrast, the Tris-based medium maintained lower but relatively stable motility, whereas the TALP-based medium showed a rapid decline in total motility from 53.1% to 15.2% during the first hour. The TALP-based medium induced the highest and most sustained protein kinase A (PKA) activity, reaching 0.047 U/mL at 0 h and 0.040 U/mL after 3 h, whereas the Hepes- and Tris-based media showed lower and less sustained activity ranging from 0.003 to 0.030 U/mL during incubation. In addition, distinct patterns of protein tyrosine phosphorylation were observed depending on the medium used. In particular, the TALP-based medium containing bicarbonate and bovine serum albumin (BSA) and the Hepes-based medium with the highest BSA concentration were associated with the highest levels of total protein tyrosine phosphorylation. Phosphoproteomic analysis further revealed condition-specific phosphorylation events, indicating that sperm maturation is dynamically regulated by the surrounding molecular environment. In contrast, no significant differences were detected in oxidative phosphorylation or in electron transport system complexes among the tested media. These findings show that differences in capacitation media composition, particularly in bicarbonate and BSA content, can markedly alter signaling outcomes and the interpretation of capacitation markers, with important implications for reproductive technologies and experimental standardization.

## 1. Introduction

Capacitation is a crucial maturation step enabling sperm to fertilize the oocyte. In vivo, it occurs in the female reproductive tract, triggering biochemical and structural changes required for fertilization [1]. Capacitation involves multiple intracellular pathways, including membrane remodeling, ion fluxes, and protein phosphorylation [2,3]. Protein tyrosine phosphorylation (PTyr) is a key marker of this process, linked to hyperactivation and fertilization signaling [3,4]. In vitro, capacitation can be induced using defined capacitation media (CMs) [5], though sperm responses to different CMs vary significantly [6].

A central regulator underlying these molecular events in boar sperm is intracellular Ca^2+^ signaling, which is fundamental to regulating sperm motility, acrosomal exocytosis, and protein phosphorylation [7]. During capacitation, bicarbonate (HCO_3_^−^) influx increases cytosolic Ca^2+^ levels [8]. This process is further modulated by voltage-gated proton (HVC1) and K^+^ channels, which promote membrane hyperpolarization and whose roles have been characterized in boar [9] and other mammalian sperm [10]. Ca^2+^ then activates calmodulin-dependent kinases, triggering serine/threonine (Ser/Thr) and subsequent Tyr phosphorylation of essential capacitation-related proteins [11].

The cyclic adenosine monophosphate–protein kinase A (cAMP–PKA) pathway is central to boar sperm capacitation. HCO_3_^−^ activates soluble adenylate cyclase (sAC), producing cAMP, which then activates PKA [12]. PKA phosphorylates Ser/Thr residues, initiating cascades that lead to PTyr and capacitation in boar and other mammalian sperm [13,14,15,16,17].

Mammalian spermatozoa rely on ATP, primarily generated via oxidative phosphorylation (OXPHOS) and glycolysis, to maintain motility and fertilization competence [18]. The metabolic pathway mainly used by spermatozoa for energy production is highly species-specific, and while OXPHOS supports general motility, glycolysis is more associated with capacitation and hyperactivation [19,20,21]. Boar spermatozoa, although commonly considered to preferentially utilize glycolysis to obtain energy, depend on mitochondrial OXPHOS for ATP production to promote motility [20]. Capacitation involves a metabolic shift, depleting glucose and citrate while increasing lactate and phosphate, suggesting that CM composition, by modulating substrate availability, critically affects ATP production, PTyr signaling, motility, and overall capacitation efficiency [18,21].

As highlighted in our review [6], boar sperm CMs vary in composition across studies, with both common and unique additives potentially influencing the signaling pathways that initiate capacitation. The selection of the three CMs was based on their repeated use in boar sperm capacitation studies and their distinct compositions, allowing for a representative comparison of commonly applied formulations. The Hepes-based medium has long been used both in ours [22,23] and in other studies on sperm capacitation [24,25,26,27,28]. The TALP-based medium represents a widely accepted model for capacitation [5,29,30], while the Tris-based medium, although minimalistic, has been used in several studies describing capacitation-associated events [31,32,33,34]. The inclusion of this simplified formulation was intentional, as it enabled us to evaluate whether even low-buffered, low-BSA systems can induce detectable capacitation-related markers, as previously observed by Ded et al. (2019) [32]. We hypothesize that such variations impact/alter phosphorylation-dependent signaling underlying capacitation, including PKA-mediated pathways and motility regulation. As no single biochemical marker fully captures the complexity of capacitation, we applied a complementary analytical approach integrating multiple assays analyzing calcium distribution, PKA activity, OXPHOS, PTyr patterns, and phosphoproteome in spermatozoa incubated in three differently composed CMs. Overall, this study targets the need for the standardization of in vitro capacitation protocols, ensuring greater reproducibility and optimization of boar sperm capacitation for assisted reproductive technologies.

## 2. Results

### 2.1. Assessment of Sperm Capacitation Status by Chlortetracycline (CTC) Fluorescence Patterns

**Figure 1:**
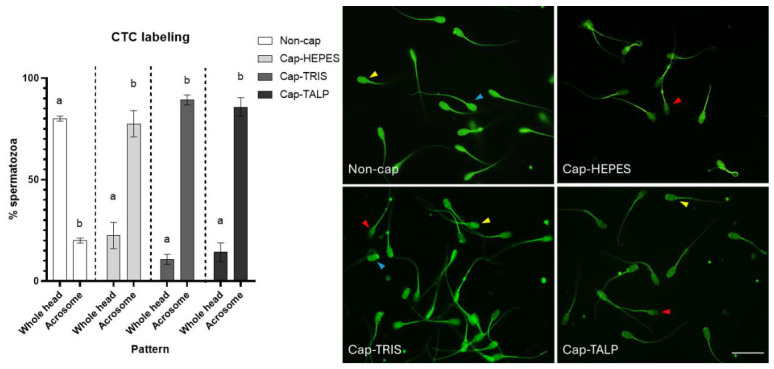
Detection of capacitation-associated events using chlortetracycline (CTC) in sperm samples. The graph shows the percentage of spermatozoa presenting specific patterns (as shown in the images); letters (a, b) show statistical significance between individual patterns inside the sperm group (Non-cap—non-capacitated spermatozoa, Cap-HEPES—spermatozoa capacitated in Hepes-based medium, Cap-TRIS—spermatozoa capacitated in Tris-based medium, Cap-TALP—spermatozoa capacitated in TALP-based medium) (*p* < 0.05); error bars represent SEM. Blue arrows indicate spermatozoa with strong fluorescence over the entire head (Whole head), yellow arrows indicate spermatozoa with strong fluorescence over the acrosome and with a weaker signal in the postacrosomal region (Acrosome), and red arrows indicate acrosomally reacted spermatozoa (omitted from analysis, the percentage of acrosomally reacted spermatozoa in individual groups is shown in the Supplementary Figure S1); scale = 20 µm; *n* = 5 independent biological replicates, each replicate consisted of a pooled ejaculate from three boars.

The analysis was based on five independent biological replicates (*n* = 5), each consisting of a pooled ejaculate from three boars, at least 200 spermatozoa per sample were evaluated. Figure 1 displays data from the fluorescent CTC assay. It shows the percentage of spermatozoa presenting two predominant patterns—Whole head (strong fluorescence over the whole head) and Acrosome (strong fluorescence over the acrosome with a lower signal in the postacrosomal region). Based on established interpretations of CTC staining, the Whole head pattern is characteristic for non-capacitated spermatozoa (Non-cap) [32], whereas the Acrosome pattern reflects capacitation-associated redistribution of intracellular Ca^2+^ and was therefore considered to be indicative of spermatozoa undergoing capacitation-associated changes. The Whole head pattern was specific for Non-cap sperm (80%), while all three in vitro capacitated (IVC) groups (spermatozoa capacitated in Hepes-based medium—Cap-HEPES, spermatozoa capacitated in Tris-based medium—Cap-TRIS, spermatozoa capacitated in modified Tyrode’s albumin lactate pyruvate medium—Cap-TALP) showed this pattern significantly less (Cap-HEPES—22.5%, Cap-TRIS—10.7%, and Cap-TALP—14.2%; *p* < 0.0001). The Acrosome pattern was represented in 20% in the Non-cap group. The Acrosome pattern was the most represented in the Cap-TRIS (89.3%), Cap-TALP (85.8%), and Cap-HEPES (77.5%) groups, indicating a predominance of capacitation-associated Ca^2+^ redistribution under these conditions. Together, these results confirm that spermatozoa incubated in all three CMs underwent capacitation-associated calcium redistribution.

### 2.2. Time-Dependent Modulation of Sperm PKA Activity During IVC

**Figure 2:**
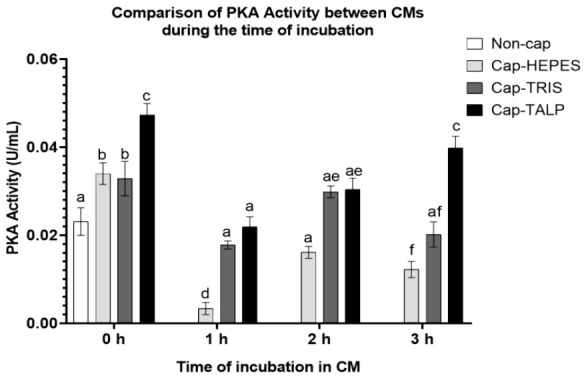
Protein kinase A (PKA) activity determination. Comparison of the differences between sperm groups, incubated in different capacitation media (CMs) (Non-cap—non-capacitated spermatozoa, Cap-HEPES—spermatozoa capacitated in Hepes-based medium, Cap-TRIS—spermatozoa capacitated in Tris-based medium, Cap-TALP—spermatozoa capacitated in TALP-based medium) at individual measurement time points. For the Non-cap sperm group, PKA activity was evaluated only at 0 h. Error bars represent SEM; statistically significant differences (*p* < 0.05) in PKA activity are indicated by letters; *n* = 4 independent biological replicates, each replicate consisted of a pooled ejaculate from three boars. Complementary statistical perspective of the same data is shown in Supplementary Figure S2, highlighting within-group temporal dynamics of PKA activity during incubation. This dual presentation allows a clearer visualization of both medium-dependent and time-dependent effects on PKA signaling.

This analysis was based on four independent biological replicates (*n* = 4), each consisting of a pooled ejaculate from three boars. Tukey’s multiple comparisons test revealed a significant increase in PKA activity (Figure 2) immediately after spermatozoa were added to all three types of CMs: Cap-HEPES 0 h–0.034 U/mL, Cap-TRIS 0 h–0.033 U/mL, and Cap-TALP 0 h–0.047 U/mL. Moreover, the Cap-TALP 0 h group exhibited significantly higher activity compared to both Cap-HEPES 0 h and Cap-TRIS 0 h. After one hour of incubation, a significant decrease in PKA activity was observed across all three IVC groups, with Cap-HEPES showing a tenfold decrease (from 0.034 U/mL to 0.003 U/mL) to levels even lower than those measured in the Non-cap group (0.023 U/mL). At 2 h, PKA activity increased again in all groups; however, Cap-HEPES maintained the lowest activity level (0.016 U/mL), which remained significantly lower compared to the other media (Cap-TRIS—0.030 U/mL, Cap-TALP—0.030 U/mL). After three hours, an increase in activity was detected only in the Cap-TALP group (0.040 U/mL), while Cap-TRIS exhibited a further decrease (0.020 U/mL) and Cap-HEPES showed no change (0.020 U/mL) compared to the two-hour time point. Overall, PKA activity increased rapidly upon exposure to capacitation media, subsequently decreased, and partially recovered over time. Among the tested conditions, TALP-based medium showed higher and more sustained PKA activity than Hepes- and Tris-based media. It is consistent with TALP-based medium containing higher bicarbonate concentration and caffeine, which may enhance PKA activation.

### 2.3. Sperm Motility Parameters and Clusters Distribution During IVC

**Figure 3:**
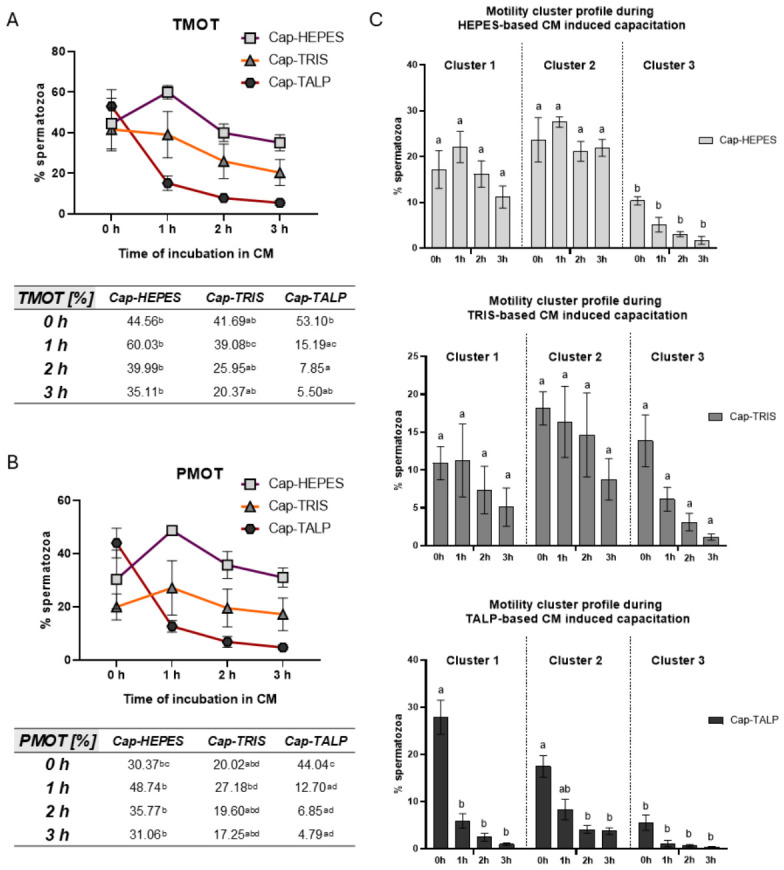
Motility evaluation during sperm in vitro capacitation in Non-cap (non-capacitated spermatozoa), Cap-HEPES (spermatozoa capacitated in Hepes-based medium), Cap-TRIS (spermatozoa capacitated in Tris-based medium), Cap-TALP (spermatozoa capacitated in TALP-based medium) during the time of incubation. Data are presented as means ± SEM. Means for (**A**) Total motility (TMOT) and (**B**) progressive motility (PMOT) are shown in the tables below graphs with letters indicating significant differences (*p* < 0.05) in three replicates. (**C**) K-means cluster analysis evaluation of sperm motility showing the percentual distribution of motile spermatozoa (VAP ≥ 15) of individual sperm groups between 3 clusters. Cluster 1—motile spermatozoa, Cluster 2—progressively motile spermatozoa, Cluster 3—spermatozoa presenting hyperactivated motility; different letters indicate significant differences (*p* < 0.05); *n* = 3 independent biological replicates, each replicate consisted of a pooled ejaculate from three boars.

Total (TMOT) and progressive (PMOT) sperm motility as well as individual kinematic parameters were measured using computer-assisted sperm analysis (CASA) during sperm incubation in CMs at 0, 1, 2, and 3 h (Figure 3). The analysis was based on three independent biological replicates (*n* = 3), each consisting of a pooled ejaculate from three boars, at least 200 spermatozoa per sample were evaluated. Significant differences were detected between individual sperm groups during incubation. In Cap-HEPES, both TMOT and PMOT increased during the first hour of incubation, and this group showed the highest TMOT and PMOT values for the remainder of the incubation. Cap-TRIS showed the lowest values at 0 h compared to the other IVC groups, but these values remained stable throughout the incubation period. The most significant differences during incubation were observed in the Cap-TALP group, which showed the highest TMOT and PMOT at 0 h. However, during the first hour of incubation, TMOT and PMOT decreased significantly and remained at low values until the end of incubation.

Motile spermatozoa (average path velocity; VAP ≥ 15) were analyzed for individual kinematic parameters and classified by k-means clustering into three groups (Figure 3). All CASA-derived kinematic parameters were initially evaluated; however, because several variables were highly correlated, only VSL, STR, ALH, and BCF were used for k-means clustering in order to define non-redundant motility subpopulations. Cluster 1 included sperm with low amplitude of lateral head displacement (ALH), low straight-line velocity (VSL), high straightness (STR), and low beat cross frequency (BCF); Cluster 2 showed high ALH, VSL, STR, and BCF (progressive motility); and Cluster 3 had high ALH but low VSL, STR, and BCF (hyperactivated motility). The clustering results are parallel to the TMOT and PMOT analyses. Motile sperm in Hepes-based medium had higher representation in Clusters 1 and 2, with no time-dependent change. In Tris-based medium, the distribution was constant. Cap-TALP spermatozoa showed greater representation in Clusters 1 and 2 only at 0 h.

### 2.4. Sperm Oxidative Phosphorylation During the Course of IVC

High-resolution respirometry was performed to assess oxidative phosphorylation (OXPHOS) activity in spermatozoa incubated in three CMs (Cap-HEPES, Cap-TRIS, Cap-TALP) for 0, 1, 2, and 3 h. The analysis was based on four independent biological replicates (*n* = 4). Routine respiration reflecting physiological OXPHOS utilization controlled by actual adenosine triphosphate (ATP) demand, LEAK (after inhibiting ATP production by OXPHOS using oligomycin), and maximal capacity of the electron transport chain (electron transport system—ETS after carbonyl cyanide p-trifluoromethoxyphenyl-hydrazone—FCCP) were evaluated.

**Figure 4:**
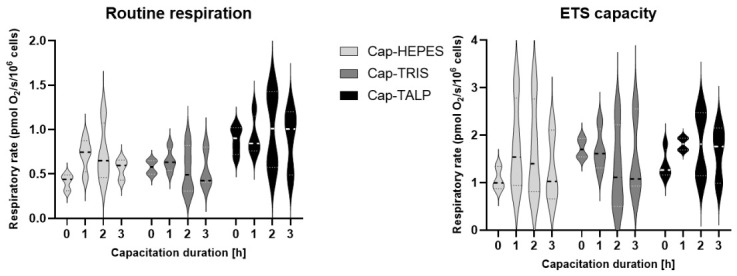
Oxidative phosphorylation evaluation by routine respiration and electron transport system capacity analysis. Data are presented as a violin plot of individual sperm groups (Cap-HEPES—spermatozoa capacitated in Hepes-based medium, Cap-TRIS—spermatozoa capacitated in Tris-based medium, Cap-TALP—spermatozoa capacitated in TALP-based medium) at time 0, 1, 2, and 3 h; ETS—electron transport system; *n* = 4 independent biological replicates.

Data show notable variation in respiratory rates between individual boars. Importantly, across all incubation times, no significant differences were detected in routine respiration or in the maximal electron transport chain capacity between the three CMs (Figure 4). Similarly, LEAK oxygen consumption values remained comparable among groups. These results indicate that OXPHOS activity in boar spermatozoa is not significantly altered during capacitation per se and is not directly influenced by CM composition or incubation time.

**Figure 5:**
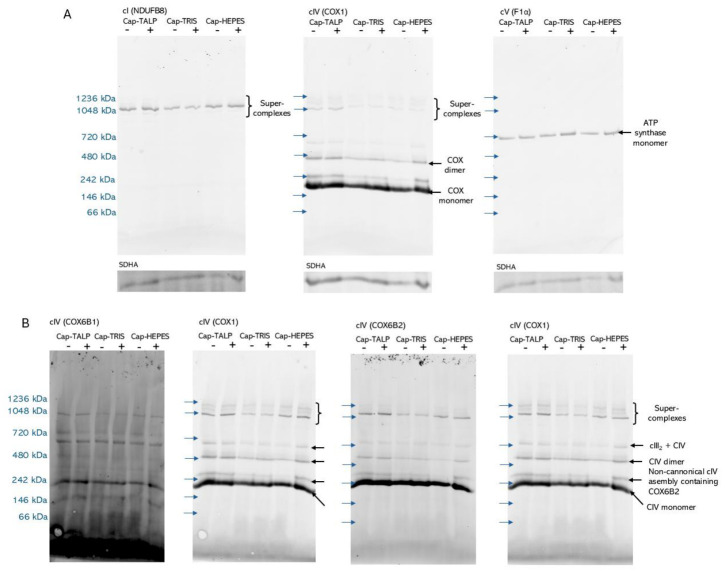
Blue Native-PAGE analysis of mitochondrial OXPHOS complexes in capacitated boar spermatozoa. Representative Western blots showing the distribution of selected mitochondrial OXPHOS complexes in sperm samples incubated under capacitating (+) and non-capacitating (–) conditions for 3 h in three different media (Cap-HEPES—spermatozoa capacitated in Hepes-based medium, Cap-TRIS—spermatozoa capacitated in Tris-based medium, Cap-TALP—spermatozoa capacitated in TALP-based medium). (**A**) Panel displays complex I (NDUFB8), complex IV core subunit (COX1), complex V—F1 α-subunit (F1α). (**B**) Panel shows COX6B1, a canonical complex IV subunit, and COX6B2, a sperm/testis-specific isoform of COX6B1, both analyzed in parallel with the core subunit of complex IV COX1 (double labeling using two secondary antibodies with distinct fluorescence properties). Loading control represented as Succinate dehydrogenase complex flavoprotein subunit A (SDHA; complex II). Markers indicate apparent molecular weights in kDa, blue arrows represent the molecular marker, black arrows represent the detected complexes and subunits.

To further evaluate possible changes in OXPHOS during capacitation, we compared the pattern of OXPHOS complexes in spermatozoa prior to and after 3 h of capacitation using blue native polyacrylamide gel electrophoresis (BN-PAGE) in 10^9^ sperm cell aliquots solubilized by mild detergent digitonin. Complex I (cI) was quantitatively associated with respiratory supercomplexes (a single band overlapping with the supercomplex form of cIV), a pattern typically seen in mammalian cells (Figure 5A). ATP synthase (complex V, cV) was detected only as monomer (Figure 5A), these solubilization conditions in mammalian cells and tissues usually preserve also cV dimers and oligomers. Most of complex IV is observed in its monomeric form; however, other canonical assemblies, such as cIV dimers and respiratory supercomplexes, were observed (Figure 5A,B). These entities contained the canonical cytochrome c oxidase subunit 6B1 (COX6B1), as well as its non-canonical testis/sperm-specific variant COX6B2. However, only COX6B2 was clearly detected in a distinct cIV assembly migrating at approximately 300 kDa, suggesting the existence of a cIV entity specific to spermatozoa. All three capacitation conditions did not cause acute changes in the BN-PAGE pattern (Figure 5A). This is also true for the distribution of COX6B1- and COX6B2-containing cytochrome c oxidase (COX) assemblies (Figure 5B), suggesting no acute reorganization of pre-existing COX and other OXPHOS complexes (i.e., shifts between monomeric forms, higher-order assemblies, and respiratory supercomplexes) occurs within the 3 h capacitation time window.

### 2.5. Distribution of Sperm PTyr Fluorescence Patterns During IVC

**Figure 6:**
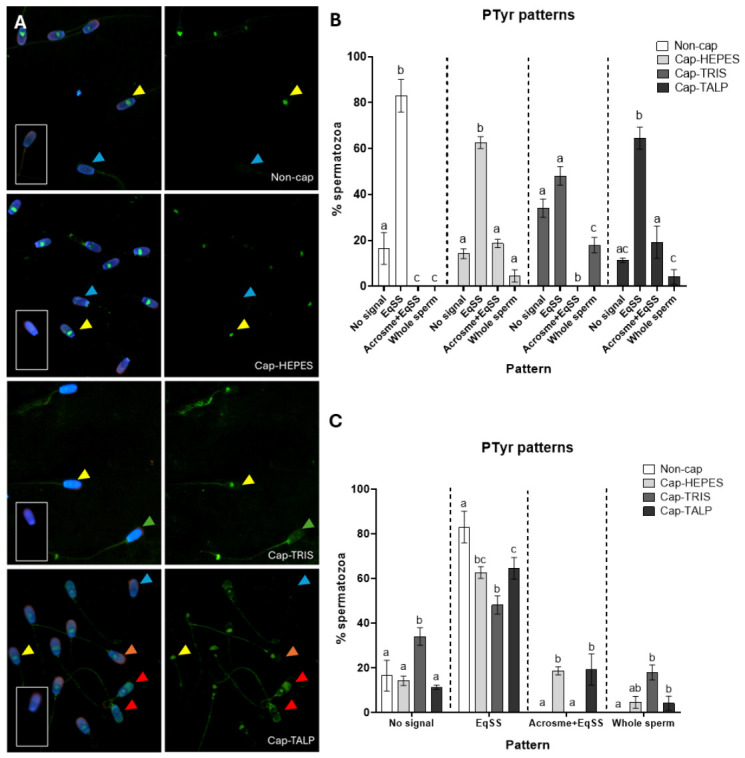
Phosphotyrosine fluorescent patterns in boar spermatozoa incubated in different CMs. Representative images of indirect immunofluorescence of individual groups are shown (**A**). Green color (AlexaFluor 488)—immunofluorescence presenting the reaction of the antibody with antigen; red color (Rhodamine)—PNA lectin staining of sperm acrosome; blue color (DAPI)—staining of the cell nucleus; arrows show different fluorescent patterns (blue—No signal, yellow—EqSS, orange—Acrosome + EqSS, green—Whole sperm, red—spermatozoa without acrosome omitted from counting); negative controls are inserted in corners. Graphs (**B**,**C**) show the percentage of spermatozoa presenting different specific immunofluorescent patterns in two ways. (**B**) The graph compares differences between the percentage of spermatozoa showing individual patterns (No signal, EqSS, acrosome+EqSS, and whole sperm) inside each group (Non-cap—non-capacitated spermatozoa, Cap-HEPES—spermatozoa capacitated in Hepes-based medium, Cap-TRIS—spermatozoa capacitated in Tris-based medium, Cap-TALP—spermatozoa capacitated in TALP-based medium). (**C**) The graph shows differences between the individual groups within specific patterns. Error bars show SEM; statistically significant differences (*p* < 0.05) are indicated by letters; *n* = 3 independent biological replicates, each replicate consisted of a pooled ejaculate from three boars.

While CTC staining was used as the primary criterion to confirm capacitation-associated events, PTyr detection served to characterize associated signaling changes and to evaluate the influence of medium composition on phosphorylation dynamics. Figure 6 shows the distribution of PTyr fluorescence patterns detected with the 4G10 antibody. This analysis was based on three independent biological replicates (*n* = 3), each consisting of a pooled ejaculate from three boars, at least 200 spermatozoa per sample were evaluated. Four patterns were identified: No signal, EqSS (equatorial subsegment) signal, Acrosome + EqSS, and Whole sperm. The No signal pattern predominated in Cap-TRIS sperm (34%) but was less frequent (11–17%) in other groups (Non-cap, Cap-HEPES, and Cap-TALP). The EqSS pattern was most common overall (Non-cap 83%, Cap-HEPES 62.6%, Cap-TRIS 48.1%, Cap-TALP 64.5%). The Acrosome + EqSS pattern occurred only in Cap-HEPES and Cap-TALP, 18.6% and 19.2% respectively, while the Whole sperm pattern appeared exclusively in capacitated samples at low frequency (Cap-HEPES—4.6%, Cap-TRIS—17.9%, and Cap-TALP—4.1%). Even though we detected patterns that occurred only in the IVC groups (Acrosome + EqSS and Whole sperm), these patterns occurred in a small percentage (less than 20%), and even in the IVC samples, the EqSS pattern prevailed. Although PTyr labeling is considered a capacitation marker, EqSS predominated in both Non-cap and IVC groups, indicating limited discriminative value. Only Cap-TRIS differed significantly from Non-cap, showing reduced EqSS (*p* < 0.0001) and increased No signal (*p* < 0.05).

### 2.6. Changes in Sperm Phosphoproteome During IVC

**Figure 7:**
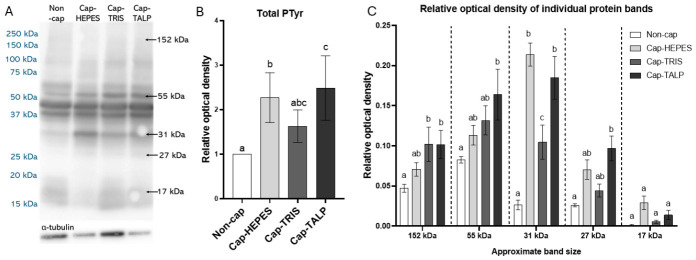
Phosphotyrosine (PTyr) detection in boar sperm protein extracts from spermatozoa after IVC in different capacitation media. Representative Western blot shows the molecular weights of detected phosphoprotein bands (**A**). Graphs show the relative optical density of Total PTyr (**B**) and individual protein bands (**C**). Error bars show SEM; statistically significant differences (*p* < 0.05) in total PTyr (**B**), or within one protein band (**C**), are indicated by letters; Non-cap—non-capacitated spermatozoa, Cap-HEPES—spermatozoa capacitated in Hepes-based medium, Cap-TRIS—spermatozoa capacitated in Tris-based medium, Cap-TALP—spermatozoa capacitated in TALP-based medium; *n* = 6 independent biological replicates, each replicate consisted of a pooled ejaculate from three boars.

Differences in PTyr levels were observed among sperm incubated in Hepes-, Tris-, and TALP-based capacitation media (Figure 7). This analysis was based on six independent biological replicates (*n* = 6), each consisting of a pooled ejaculate from three boars. Total PTyr was significantly higher (*p* < 0.05) in Cap-HEPES and Cap-TALP sperm compared to Non-cap, while Cap-TRIS showed no significant difference. Several PTyr-reactive protein bands were consistently detected in all samples, while the 17, 27, 31, 55, and 152 kDa proteins varied in relative optical density (ROD) between groups. Significant differences were found for the 152 kDa band (Non-cap vs. Cap-TRIS, Cap-TALP), 55 kDa (higher in Cap-TALP), 31 kDa (higher in all IVC groups), and 27 kDa (higher in Cap-TALP). The 17 kDa band showed no significant change but was inconsistently present in Non-cap and Cap-TRIS samples.

**Figure 8:**
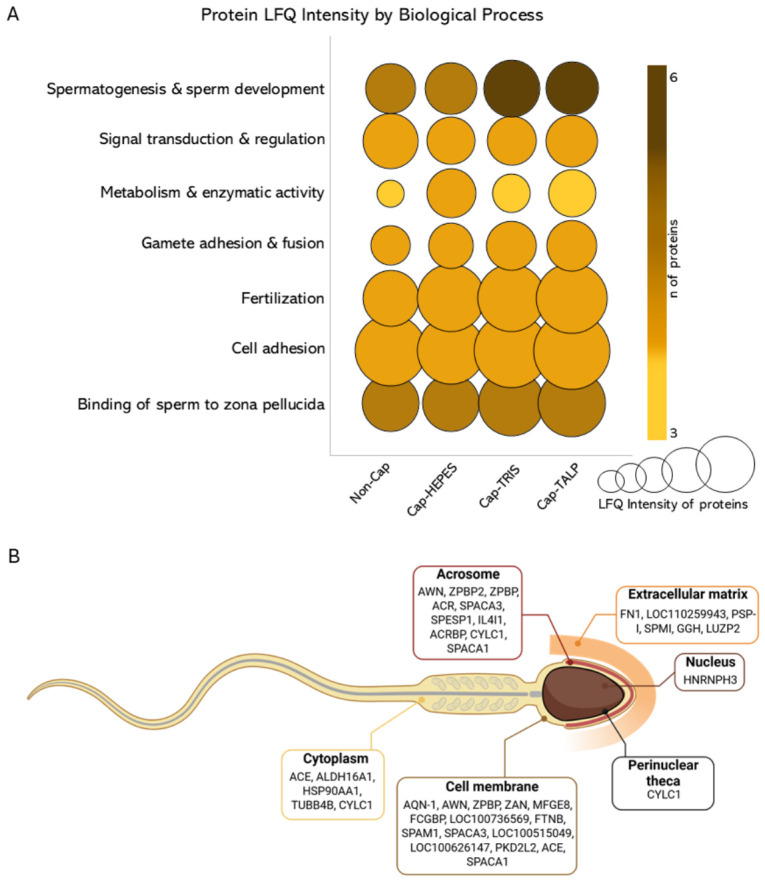
Functional distribution and cellular localization of specific identified phosphorylated sperm proteins with significant differences between sperm groups in Label-free quantification (LFQ) Intensity. (**A**) The bubble chart shows the distribution of proteins across biological processes derived from experimental phosphoproteomic analysis. The size of the bubble indicates the average LFQ Intensity of proteins involved in individual processes within individual sperm groups (Non-cap—non-capacitated spermatozoa, Cap-HEPES—spermatozoa capacitated in Hepes-based medium, Cap-TRIS—spermatozoa capacitated in Tris-based medium, Cap-TALP—spermatozoa capacitated in TALP-based medium), and the color of the bubble indicates the number of proteins participating in the biological process. (**B**) The diagram represents a schematic illustration of sperm cell structure showing the localization of identified proteins based on experimental phosphoproteomic data and UniProt database annotation: acrosin (ACR), acrosin-binding protein (ACRBP), aldehyde dehydrogenase 16 family member A1 (ALDH16A1), amine oxidase (IL4I1), angiotensin-converting enzyme (ACE), angiotensin-converting enzyme (LOC100515049), carbohydrate-binding protein AQN-1 (AQN-1), carbohydrate-binding protein AWN (AWN), cylicin 1 (CYLC1), disintegrin and metalloproteinase domain-containing protein 2 (FTNB), fibronectin (FN1), folate gamma-glutamyl hydrolase (GGH), heat shock protein HSP 90-alpha (HSP90AA1), heterogenous nuclear ribonucleoprotein H3 (HNRNPH3), hyaluronidase (SPAM1), jacalin-type lectin domain-containing protein (LOC110259943), leucine zipper protein 2 (LUZP2), leucine-rich repeat-containing protein 37A2 (LOC100626147), major seminal plasma glycoprotein PSP-I (PSP-I), membrane cofactor protein (LOC100736569), milk fat globule EGF and factor V/VIII domain containing (MFGE8), polycystin-2-like protein 2 (PKD2L2), sperm acrosome membrane-associated protein 1 (SPACA1), sperm acrosome membrane-associated protein 3 (SPACA3), sperm equatorial segment protein 1 (SPESP1), spermadhesin AQN-3-like protein (SPMI), tubulin beta chain (TUBB4B), VWFD domain-containing protein (FCGBP), zona pellucida binding protein 2 (ZPBP2), zona pellucida-binding protein 1 (ZPBP), zonadhesin (ZAN); further described in Table 1 (Panel (**B**) was created in BioRender. Pilsová, A. (2025) https://BioRender.com/rfp1q4j, accessed on 8 May 2026).

Mass spectrometry analysis identified a subset of phosphorylated proteins across all experimental groups (Non-cap, Cap-HEPES, Cap-TRIS, Cap-TALP). This analysis was based on three independent biological replicates (*n* = 3), each consisting of a pooled ejaculate from three boars. Several proteins contained multiple phosphorylation sites, indicating isoform variability or dynamic phosphorylation states. Analysis of variance (ANOVA) of label-free quantification LFQ intensities revealed significant differences in 31 phosphoproteins (Figure 8 and Table 1). Among the identified phosphoproteins, several proteins associated with fertilization-related sperm functions showed significant LFQ differences between sperm groups. Notably, increased LFQ intensities were found for ZPBP1, ZPBP2, acrosin (ACR), and sperm hyaluronidase SPAM1, which contribute to zona pellucida recognition, acrosomal remodeling, and sperm penetration through the cumulus oophorus and zona pellucida, processes essential for successful fertilization. Functional annotation (UniProt; Figure 8) classified these proteins into seven categories: Binding to the zona pellucida, Cell adhesion, Fertilization, Gamete adhesion and fusion, Metabolism and enzymatic activity, Signal transduction and regulation, and Spermatogenesis and sperm development.

**Figure 9:**
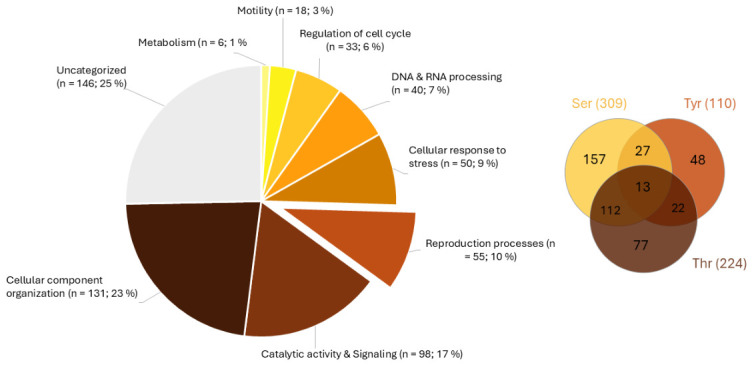
Functional distribution of identified phosphoproteins and residue-specific phosphorylation profile of sperm proteins. The pie chart shows the distribution of phosphoproteins across biological processes. The number and percentages of sperm phosphoproteins is in the pie chart legend. The categories are made based on the UniProt and Gene Ontology database. The Venn diagram illustrates the overlap of phosphoproteins phosphorylated on serine (Ser), threonine (Thr), and tyrosine (Tyr) residues, showing number of proteins that show single or multiple phosphorylation sites. The Venn diagram has been made using InteractiVen web-based tool [35].

However, phosphorylation was not detected in all 31 proteins. Supplementary Material contains a table of these proteins, where phosphorylation sites detected by mass spectrometry are recorded. For proteins in which phosphorylation was not detected, a map of possible phosphorylation sites based on FASTA and generic prediction using NetPhos-3.1 (DTU Health Tech) is provided (Supplementary Table S1). Supplementary material also includes a list of proteins for which phosphorylation was detected by MS, along with the specific phosphorylation sites (Supplementary Table S2). The functional categorization of all identified phosphoproteins is summarized in Figure 9, which illustrates their distribution across major biological processes such as catalytic activity and signaling, cellular organization, stress response, reproduction, and metabolism. The pie chart shows both the number and percentage representation of sperm proteins within each category, based on annotation in the UniProt and Gene Ontology databases. The Venn diagram in Figure 9 further illustrates the overlap of phosphoproteins phosphorylated on serine, threonine, and tyrosine residues, highlighting the number of proteins carrying single or multiple types of phosphorylation sites. All phosphoproteins included in this analysis are listed in Supplementary Table S2.

## 3. Discussion

Capacitation media (CMs) are designed to mimic the oviductal environment in which capacitation naturally occurs in vivo [5]. However, no standardized CM has been established, and compositions used among laboratories vary substantially. The present study aimed to demonstrate that such compositional differences can influence the course of capacitation and thereby alter the reliability and interpretation of its established biochemical markers.

To confirm capacitation in the tested media, spermatozoa were analyzed using the CTC fluorescence assay, a standard method for assessing capacitation status [32,36]. Although widely used, the method is partly subjective; therefore, all evaluations were performed immediately after staining by the same examiner to minimize variability and ensure reliable fluorescence assessment. In this assay, the Whole head fluorescence pattern is characteristic of non-capacitated, acrosome-intact spermatozoa, whereas the Acrosome pattern, defined by a loss of fluorescence from the postacrosomal region with retention over the acrosome, is widely accepted as indicative of capacitated but acrosome-intact sperm. Significant differences between non-capacitated and IVC spermatozoa confirmed that capacitation-associated events occurred in all CMs (Hepes-, Tris-, and TALP-based).

Since the CTC assay confirmed capacitation-associated events in all tested media, we examined upstream signaling events initiating this process, focusing on PKA activity, a key regulator of the capacitation cascade. An immediate increase in PKA activity occurred after exposure to CMs. Both Hepes- and TALP-based media contained bicarbonate, whereas the Tris-based medium was CO_2_ saturated. This likely reflects bicarbonate uptake via the Na^+^/HCO_3_^−^ cotransporter (NBC), activating soluble adenylyl cyclase (sAC), elevating cAMP, and stimulating PKA-dependent Ser/Thr phosphorylation [37], eventually leading to increased PTyr [38]. The bicarbonate-induced cAMP rise may also affect membrane hyperpolarization, intracellular pH, and Ca^2+^ influx, all crucial for capacitation [10,17]. Bicarbonate influx and cholesterol efflux are closely linked [39]. Cholesterol removal by BSA may, in turn, activate transporters and channels such as the NBC and CatSper cation channels, further stimulating the cAMP–PKA pathway [16]. Notably, PKA activity was relatively high even in Non-cap spermatozoa, suggesting partial capacitation or priming due to PBS washing, which may remove seminal plasma-derived decapacitation factors [40]. Within our experimental setting, the Non-cap group thus reflects a washed baseline rather than a completely inactive physiological state. Therefore, the differences among groups (Non-cap, Cap-HEPES, Cap-TRIS, and Cap-TALP) reflect variations in the level and timing of capacitation-related activation, rather than a simple comparison between non-capacitated and capacitated spermatozoa.

The transient decrease in PKA activity after one hour likely reflects an early regulatory response to the CM. The initial exposure probably triggers a rapid intracellular cAMP rise, activating PKA [38], followed by feedback adjustments that temporarily reduce its activity, possibly via phosphodiesterase or protein phosphatases, or cofactors depletion. A similar triphasic protein phosphorylation response, such as a rapid increase, transient decline, and subsequent gradual rise, has been described previously [38]. The presence of bicarbonate (or CO_2_) in the medium causes a sharp increase in intracellular cAMP, which then decreases and gradually rises again [41,42]. The TALP-based medium, containing caffeine, a nonspecific phosphodiesterase inhibitor, may sustain higher intracellular cAMP and consequently enhance PKA activity [43].

Apart from the 0 h time point, the Hepes-based medium consistently showed the lowest level of PKA activity throughout the entire monitoring period, with the lowest value after 1 h. Even though the Hepes-based CM contained HCO_3_^−^ (2 mM), this concentration was much lower than in TALP-based CM (25 mM), which showed the highest PKA activity. Indeed, boar spermatozoa can capacitate even in HCO_3_^−^-free media when sufficient BSA (5 mg/mL) is present [44]. Thus, despite low PKA activity in some conditions, capacitation can still proceed, possibly through alternative or compensatory signaling pathways.

Although PKA activity effectively reflects activation of the cAMP–PKA pathway, it may not directly predict sperm fertilizing competence, as downstream phosphorylation events and mechanical parameters, such as motility and acrosomal responsiveness, can be modulated independently. The relationship between PKA activity and fertilization competence may be more complex than a direct linear association with sperm motility. As suggested by Zapata-Carmona et al. (2020) [45], porcine oviductal fluid decreases PKA activity, PTyr, and the acrosome reaction in a concentration-dependent manner, likely serving to delay capacitation until sperm reach the oocyte. This indicates that PKA activation must be finely regulated, as both insufficient and premature activation can compromise fertilization success. In our study, the observed differences in PKA activity between media may therefore reflect varying degrees of physiological readiness rather than serve as a simple indicator of functional outcomes. Despite the observation that capacitation-associated signaling events, such as PKA activity, were most pronounced in sperm incubated in TALP-based medium, this group exhibited markedly reduced motility. This paradox suggests a potential decoupling between molecular markers of capacitation and actual sperm functionality under specific incubation conditions. TALP is widely used in in vitro fertilization (IVF) systems, where spermatozoa are additionally exposed to stimulatory signals from oocytes or cumulus–oocyte complexes (COCs) [46]. The absence of these signals in our experimental setup may have limited the CM capacity to promote motility. COCs produce significant amounts of nitric oxide, which supports sperm capacitation, motility, and subsequent fertilization [30,47]. The beneficial effect of sperm co-incubation with COCs on sperm motility has also been demonstrated in humans [48].

The highest sperm motility was observed in the Hepes-based medium, which contained the greatest BSA concentration among the tested media. Elevated BSA levels likely increase the viscoelasticity of the medium, a property known to facilitate collective sperm movement [49] and thus explain the enhanced motility observed. In media with lower viscoelasticity, sperm tend to exhibit rolling motion, whereas media enriched with polymers that increase viscoelasticity promote planar flagellar beating near solid surfaces. Considering that motility was evaluated along a solid surface (Makler’s chamber), sperm moving in a thin viscoelastic layer may experience reduced fluid resistance, which could further support their movement compared with that in a TALP-type medium [50].

In addition to viscoelastic properties, the energy substrates present in each medium may also contribute to the observed differences in sperm motility, which is tightly linked to cellular energy metabolism. Tris-based media, which contains 10-fold less glucose and pyruvate, showed the lowest TMOT and PMOT values at 0 h but maintained relatively stable motility throughout the incubation period. Limited substrate availability may reduce motility levels but also prevent rapid ATP depletion, thereby stabilizing motility over time. Interestingly, recent study demonstrated that low glucose conditions may promote sustained linear motility [51]. Hepes-based medium contains a significantly higher concentration of pyruvate, up to fivefold compared to TALP, as well as sorbitol, which may serve as an alternative substrate for glycolysis [52]. This medium therefore perhaps more effectively supports both glycolytic and oxidative ATP production, and TMOT and PMOT increased during the first hour of incubation and remained elevated. Sperm incubated in TALP exhibited the highest TMOT and PMOT at 0 h, followed by a significant decline during the incubation. TALP-based media are characterized by high lactate availability, which can efficiently support oxidative metabolism and sustain ATP production in the short term. However, the lower levels of glycolytic substrates, such as glucose and pyruvate, may limit long-term ATP production, which is required for sustained motility. This implies that despite having similar energy production capacities, sperm in TALP-based CM are unable to translate this into effective motility, possibly due to the absence of appropriate external stimuli such as chemotactic cues, paracrine signals from COCs, or mechanical modulation from surrounding structures. Therefore, supplementation of TALP-based CM with physiologically relevant factors, such as low concentrations of progesterone (2.53 ng/mL), a compound naturally present in periovulatory fluid [53] could improve its functional outcomes, such as hyperactivation and fertilizing ability [45,54]. Although all three media assessed here are widely used for boar sperm functional studies, the distinct energetic environments they provide should be considered when interpreting results. The rapid decline of the hyperactivated sperm subpopulation (Cluster 3) observed in TALP-based medium may reflect the high energetic demands associated with hyperactivated motility. Hyperactivation is characterized by asymmetrical high-amplitude flagellar beating and increased ATP consumption [55]. In TALP-based medium, the combination of high bicarbonate concentration and caffeine likely promotes strong activation of the cAMP–PKA pathway, leading to rapid induction of capacitation-associated signaling and hyperactivation. However, despite sustained PKA activity, the lower availability of glycolytic substrates, such as glucose and pyruvate, may limit the long-term maintenance of hyperactivated motility during prolonged incubation. Although intracellular ATP levels, oxidative stress, and pH changes were not directly measured in this study, the observed motility dynamics are consistent with a potential decoupling between capacitation-associated signaling and sustained sperm functionality under TALP incubation conditions. This interpretation is further supported by the numerical increase in ETC during capacitation, accompanied by a decline in the percentage of motile spermatozoa and motile subpopulations, which may indicate stress-related mitochondrial activation. These findings suggest that this medium may not be suitable for long-term incubation of boar spermatozoa.

Depending on the balance of available energy substrates, signaling stimuli, and physical properties of the medium, spermatozoa progressed through capacitation via different combinations of intracellular pathways. In our study, these medium-specific responses were accompanied by coordinated changes across multiple capacitation-related processes, including PKA activation, PTyr dynamics, calcium-associated signaling, and sperm motility patterns. Thus, individual media promoted distinct capacitation responses rather than a uniform capacitation course. Such medium-dependent differences may influence the timing and extent of downstream events required for fertilization, including acrosomal responsiveness and the ability of spermatozoa to effectively interact with the oocyte. At the same time, fertilization outcomes are not determined by capacitation medium composition alone but are also strongly shaped by experimental protocols, including sperm concentration, incubation time, handling procedures, and the overall IVF setup used in individual laboratories [56]. This is supported by the results of various IVF studies. For instance, Matas et al. (2010) [29] compared unwashed, PBS-washed, and Percoll-washed boar spermatozoa used for IVF in TALP and found that Percoll-washed sperm achieved the highest penetration (78.4 ± 3.8%) and activation rates (89 ± 3.3%), underscoring the importance of sperm processing prior to fertilization. Soriano-Úbeda et al. (2019) [5] demonstrated that varying bicarbonate concentrations in TALP (0–25 mM) significantly altered IVF efficiency and monospermy, with optimal fertilization achieved at 15 mM HCO_3_^−^ (33.9 ± 3.7% fertilization, 48.7 ± 4.7% monospermy). Staicu et al. (2019) reported nearly 100% penetration and pronucleus formation using TALP with cumulus–oocyte complexes, though fewer sperm were bound per zona pellucida when cumulus cells were removed [30]. Suzuki et al. (2002), using a Hepes-containing Pig-FM medium, showed that seminal plasma inhibits both capacitation and fertilization, while the presence of cumulus cells reduced penetration but increased monospermy [57]. Together, these studies highlight that apparent discrepancies among fertilization outcomes likely arise from combined effects of sperm preparation methods, media composition, the presence of oviductal or cumulus components, and overall IVF setup rather than from inconsistencies in capacitation itself. Nevertheless, our findings emphasize that the choice of capacitation medium represents a critical upstream factor that should be carefully considered and standardized together with IVF protocols when interpreting fertilization-related outcomes.

As was confirmed in the recent study [58] over 80% of ATP in boar spermatozoa is produced by oxidative phosphorylation with a significant decrease over time and storage at 17 °C. In this study, the authors supposed that OXPHOS represents a major pathway of ATP synthesis, predominantly required by boar spermatozoa. These short-term preservation conditions represent a significant stressor factor. During IVC, spermatozoa are exposed to highly demanding conditions as well. Based on that, we were focused on the analysis of OXPHOS activity. Interestingly, we used a minimally invasive, highly sensitive, high-resolution respirometer. The measurements were aimed at evaluating selected capacitation media and the dynamics of respiration changes. Although OXPHOS measurements were performed using sperm from individual boars, whereas other assays were conducted on pooled ejaculates to reduce individual variability, the general tendencies remained comparable across samples. Nevertheless, inter-individual variability in metabolic activity cannot be fully excluded and may have contributed to the absence of statistically significant differences, even though consistent trends between media were observed.

We showed no significant differences in both OXPHOS parameters between media and time points. This observation suggests that changes in sperm capacitation status are not necessarily accompanied by proportional changes in mitochondrial respiration during IVC. Our results, focusing directly on the detection of capacitation markers, showed that TALP medium induces the most successful capacitation status, and in contrast, sperm motility is at the lowest level, which is in agreement with the results of Staicu et al. (2019) [30]. Importantly, although a time-dependent increase in OXPHOS activity was observed within individual media under ETS capacity measurements (i.e., maximal respiratory capacity), pronounced differences in sperm motility and phosphorylation patterns occurred despite comparable mitochondrial respiratory capacity across conditions. Taken together, the absence of differences in OXPHOS activity among media, combined with marked differences in sperm motility and phosphorylation patterns, suggests that mitochondrial ATP production capacity alone does not account for the motility changes observed under the different capacitation conditions. Rather, these findings are consistent with the possibility that ATP availability may support capacitation-associated signaling events, including protein phosphorylation. However, because intracellular ATP levels and ATP utilization were not directly measured, the relationship between ATP production, sperm motility, and phosphorylation during IVC should be interpreted with caution. This interpretation is further supported by our observation that the BN-PAGE pattern of OXPHOS complexes does not show acute changes during capacitation. Here, we refer specifically to changes in supramolecular assembly of pre-existing complexes (individual complexes versus higher-order assemblies/supercomplexes), rather than to de novo synthesis of mitochondrial proteins. Importantly, using BN-PAGE, we provide the first direct evidence of the presence of the sperm/testis-specific subunit COX6B2 [59] in complex IV complexes in spermatozoa. Our data indicate the parallel existence of complexes harboring either the ubiquitous, canonical COX6B1 or the non-canonical COX6B2. It was previously shown that mouse Cox6b2 knock-out leads to decreased sperm motility and subfertility, but without accompanying changes in OXPHOS activity [60]. In this context, our observation of the COX6B2-specific, 300 kDa cIV complex warrants further investigation. Our results provide unique insight specifically into OXPHOS and its regulative role in boar sperm capacitation-associated events. Despite previous reports emphasizing the importance of oxidative phosphorylation in sperm capacitation, our data revealed no significant differences in OXPHOS activity among media. This highlights the complexity of energy metabolism and its regulation during capacitation and provides a foundation for future studies investigating how different metabolic pathways contribute to sperm function and capacitation.

The biochemical changes triggered by CM composition are reflected in distinct patterns of PKA activity, which we found differ among the tested media. Since the cAMP/PKA signaling pathway is known to activate protein tyrosine kinases, leading to subsequent PTyr events, we anticipated that these upstream differences would culminate in specific phosphorylation of target proteins. To explore these downstream effects, we performed phosphoproteomic profiling of spermatozoa incubated in the different media. Tyrosine phosphorylation is traditionally considered a hallmark of mammalian sperm capacitation. Our study revealed that PTyr distribution and abundance in boar sperm are strongly influenced by the composition of CMs. Although the EqSS fluorescence pattern dominated in all experimental groups, its prevalence in Non-cap sperm indicates that EqSS-associated phosphorylation cannot be regarded as a unique indicator of capacitation. Therefore, as we have already mentioned in our systematic review [6], we do not consider PTyr detection by immunofluorescence to be a reliable marker of capacitation when used alone. In this study, PTyr was evaluated within the signaling context rather than as a stand-alone marker of capacitation status. As we showed in the detection of PTyr using Western blot analysis, there is indeed an increase in total PTyr during capacitation, but these changes are not easily detectable by immunofluorescence. Therefore, we used the CTC assay to detect capacitation-associated events, which gave us clear differences between non-capacitated spermatozoa and those incubated in CM. There are other markers of capacitation that can be visualized by fluorescence, such as cholesterol efflux [61], zinc ion flux [25], and changes in actin structure [32,62], which may also serve as reliable indicators of sperm capacitation status.

Our observation that PTyr immunofluorescent detection is not a sufficient stand-alone marker supports the fact that some PTyr modifications in EqSS arise during epididymal maturation rather than exclusively during capacitation [63]. The detection of phosphorylated sperm acrosome-associated protein 1 (SPACA1) in our MS data further supports that idea, as this protein localizes to the EqSS already in epididymal sperm. In contrast, earlier studies linked PTyr in EqSS to the capacitation process [3,64], suggesting that the relationship between EqSS labeling and functional capacitation may differ among species or depend on the specific components of the medium. Our results thus confirm earlier assumptions [6] that the use of PTyr as a capacitation marker should be interpreted cautiously, since it is sensitive to the ionic and protein composition of CMs.

Western blot analysis revealed differences in total PTyr levels and several protein bands among sperm groups. Total PTyr increased in Cap-HEPES and Cap-TALP but not in Cap-TRIS compared with Non-cap spermatozoa. Four protein bands differed in ROD across the studied groups. The protein detected with an approximate molecular weight of 31 kDa could be sp32, the active form of acrosome-binding protein (ACRB). ACRB is produced in the form of a zymogen (61 kDa) and is subsequently cleaved, giving rise to the active form ~35 kDa [65]. Phosphorylation of ACRB has been described in connection with capacitation-related changes [66,67], occurring specifically in the presence of Ca^2+^ and independently of cAMP/PKA signaling. Its active, phosphorylated form participates in the conversion of proacrosin to acrosin, a process required for zona pellucida penetration [4,42,66,67,68,69]. Mass spectrometry identified SPACA1 (32 kDa), consistent with the phosphorylated protein band of 31 or 27 kDa as a truncated form. SPACA1 showed significantly lower LFQ intensity in Non-cap sperm and phosphorylation at multiple residues, including Tyr. The SPACA1 protein localizes to the EqSS and the acrosomal membrane and undergoes PTyr during epididymal maturation [63]. The increase in phosphorylation observed during IVC suggests that its post-translational activation contributes to membrane remodeling and the acquisition of fertilization competence facilitating sperm–oocyte interaction [70]. The 55 kDa band, which is higher in Cap-TALP, may correspond to the sperm adhesion molecule SPAM1 (hyaluronidase) [71], identified in our MS data. SPAM1 facilitates sperm transit through the cumulus matrix and zona pellucida binding [72].

The approximately 17 kDa band, variably present among samples, probably represents several phosphorylated proteins with similar molecular weights, including members of the spermadhesin family—Major seminal plasma glycoprotein PSP-I (14 kDa), Carbohydrate binding protein AQN-1 (14 kDa), Spermadhesin AQN-3-like protein (15 kDa), and Carbohydrate binding protein AWN (17 kDa), Jacalin-type lectin domain-containing protein (LOC110259943, 18 kDa) and Sperm acrosome membrane-associated protein SPACA3 (18 kDa). Spermadhesins function as key adhesive molecules involved in sperm–oviductal and sperm–zona pellucida interactions [73,74,75,76]. Their possible variable phosphorylation patterns suggest regulated detachment from the sperm surface during capacitation, consistent with the known shedding of decapacitation factors and remodeling of surface glycoproteins [13,77,78].

In addition to these surface-associated proteins, we identified phosphoproteins involved in the regulation of intracellular capacitation signaling pathways. Among them, AKAP and GSK3 represent the main regulators of sperm function. AKAP serves as a structural scaffold that anchors PKA within the fibrous sheath, spatially restricting its signaling activity. Its phosphorylation during capacitation enhances PKA anchoring and promotes the transition to hyperactivated motility [2,79]. GSK3, a Ser/Thr kinase that suppresses premature capacitation and motility [44,80,81], was also detected in a phosphorylated (inactive) form, suggesting its inhibition by PKB and PKC following PKA activation. This sequential phosphorylation cascade reflects fine-tuned regulation of capacitation initiation and timing [81].

We also identified proteins with affinity towards zona pellucida, such as lactadherin (MFGE8) [82] and zona pellucida-binding proteins (ZPBP)—ZPBP1 and ZPBP2. ZPBP1 and 2 both exhibit higher LFQ intensities in capacitated spermatozoa, consistent with previous reports describing increased affinity of these proteins for zona pellucida glycoproteins following PTyr-associated capacitation events [13,83]. This suggests that their post-translational modification represents a key molecular switch linking capacitation signaling with fertilization ability.

Our MS data identified angiotensin-converting enzyme (ACE) as a relevant phosphoprotein, with the highest LFQ intensity in sperm incubated in TALP-based medium and the lowest in HEPES-based medium. Based on sequence similarity to human and rabbit ACE, phosphorylation at Ser1302 has been shown to promote membrane retention [84]. In contrast, PTyr within the extracellular domain enhances secretase-mediated cleavage, generating soluble ACE [85]. As membrane-bound ACE facilitates sperm–oolemma binding in humans [86], a similar regulatory mechanism might also function in boar sperm.

Mass spectrometry identified several phosphorylated proteins across experimental groups, many carrying multiple phosphorylation sites that suggest dynamic regulation (Supplementary Table S2). Some enriched proteins showed no detectable phosphorylation, likely due to phospho-group lability or partial non-specific enrichment. Functional annotation (Gene Ontology) revealed that most phosphorylated proteins participate in cellular organization, stress response, cell-cycle regulation, and spermatogenesis.

Among 31 proteins showing significant LFQ differences between sperm groups, most were associated with spermatogenesis and sperm development. In contrast, the most abundant proteins were linked to fertilization, zona pellucida binding, and cell adhesion. Four proteins related to metabolism and enzymatic activity included ALDH16A1, detected only in Cap-HEPES sperm. Although phosphorylation of this protein has not been reported, epsilon protein kinase C (εPKC), which mediates phosphorylation of mitochondrial aldehyde dehydrogenase 16 family member A1 (ALDH2), has been described [87]. ALDH contributes to sperm motility by preventing the accumulation of toxic metabolites [88], consistent with the highest motility observed in Cap-HEPES spermatozoa. We hypothesize that phosphorylation of metabolic enzymes such as ALDH16A1 and pyruvate dehydrogenase E1α further suggests a regulatory link between energy metabolism and signaling events driving capacitation, indicating that energetic and post-translational pathways are tightly interconnected. Together, these phosphoproteomic data demonstrate that capacitation-associated phosphorylation affects not only general cellular processes but also specific functional proteins directly related to motility regulation, oocyte interaction, enzymatic activation, and energy metabolism. The convergence of these modifications underscores a coordinated, multi-level control of sperm functional maturation and fertilization competence.

## 4. Materials and Methods

Unless noted, chemicals were purchased from Sigma-Aldrich (St. Louis, MO, USA).

### 4.1. Sperm Preparation

Whole ejaculates from 12 fertile 12–18-month-old Duroc boars were provided by Insemination station Skrsin LIPRA PORK a.s., Rovensko pod Troskami, Czech Republic. Some individual boars contributed samples repeatedly, but each sample was always from a different collection. In total, 46 ejaculate samples were used. The animal handling followed Council Directive 98/58/EC, Act No. 154/2000 Coll., and Act No. 246/1992 Coll. of the Czech National Council. Ejaculates were transported at a constant temperature and evaluated for sperm concentration and motility using light microscopy. Ejaculates from three boars were diluted in Phosphate-buffered saline (PBS; #P4417; Sigma-Aldrich; St. Louis, MO, USA) to 5 × 10^7^ sperm/mL and pooled together to minimize individual variability. Thus, although a total of 12 boars were used in the study, each biological replicate consisted of a pooled sample from three boars. For bioenergetic assays, sperm from single boars were used. Biological replicates, except those for bioenergetic assays, consisted of a pool of three ejaculates obtained from three boars, which was subsequently divided into four experimental groups. Thus, within each experimental day, all groups contained spermatozoa originating from the same set of three boars. The experiments were independently repeated three to six times. Seminal plasma was removed, and spermatozoa were washed three times in PBS (300× *g*, 5 min, RT). Non-capacitated spermatozoa (Non-cap; 5 × 10^7^) were stored at −20 °C for PKA activity measurement, gel electrophoresis, and phosphoproteome analysis.

### 4.2. Sperm In Vitro Capacitation (IVC)

Spermatozoa were capacitated in three CMs: Hepes-based CM (Cap-HEPES), Tris-based CM (Cap-TRIS), and TALP-based CM (Cap-TALP) (Table 2) for 3 h at 37 °C with 5% (*v*/*v*) CO_2_.

Spermatozoa in Tris-based CM were incubated in 1 mL aliquots with air access, whereas those in Hepes- and TALP-based CMs were incubated in 15 mL tubes with limited air access. After IVC, CMs were removed by centrifugation (300× *g*), and spermatozoa were washed twice with PBS. Pellets containing 5 × 10^7^ spermatozoa (Cap-HEPES, Cap-TRIS, Cap-TALP) were prepared, with additional samples collected at 0, 1, 2, and 3 h of IVC for PKA activity analysis. All samples were stored at −20 °C for PKA, electrophoresis, and phosphoproteome analysis.

### 4.3. Determination of Capacitation Using CTC

Sperm capacitation was verified using the chlortetracycline (CTC) assay. A 0.78 mM CTC solution (#C-4881) in 20 mM Tris, 130 mM NaCl, and 5 mM L-cysteine (pH 7.8) was prepared and 30 µL of spermatozoa (Non-cap, Cap-HEPES, Cap-TRIS, Cap-TALP; 3 × 10^7^/mL) were mixed with 30 µL CTC, incubated 10 min at room temperature (RT) in the dark, then fixed with 20 µL 4% paraformaldehyde. Suspension (10 µL) was mounted in Vectashield with 4′,6-diamidino-2-phenylindole (DAPI; #H-1200, Vector Laboratories, Newark, CA, USA). Sperm fluorescence was analyzed under a Nikon Eclipse Ni-U microscope (Nikon, Tokyo, Japan) using constant settings in NIS-Elements software v5.21 (Nikon). The analysis was based on five independent biological replicates (*n* = 5), each consisting of a pooled ejaculate from three boars, at least 200 spermatozoa per sample were subjectively evaluated and classified as Non-cap (uniform whole head fluorescence) or as displaying capacitation-associated events (fluorescence restricted to the acrosome) according to Ded et al. (2019) [32].

### 4.4. PKA Activity Measurement

The analysis was based on four independent biological replicates (*n* = 4), each consisting of a pooled ejaculate from three boars. PKA activity was measured using the PKA Colorimetric Activity Kit (#EIAPKA, Thermo Fisher Scientific, Waltham, MA, USA) following the manufacturer’s instructions. Frozen–thawed pellets (Non-cap, Cap-HEPES, Cap-TRIS, Cap-TALP; 0–3 h) were lysed in 150 µL of Cell Lysis Buffer containing protease/phosphatase inhibitors (#11873580001, Roche, Basel, Switzerland) and phenylmethylsulfonyl fluoride (PMSF; #6367, Carl Roth GmbH, Karlsruhe, Germany). Samples were kept on ice for 30 min with intermittent vortexing, then centrifuged (10,000× *g*, 10 min, 4 °C). Supernatants were diluted 1:15 in Reaction Buffer and analyzed per kit protocol. Absorbance was measured at 450 nm using a VERSAmax™ Microplate Reader with SOFTmax^®^ PRO software v7.1 (Molecular Devices, San Jose, CA, USA).

### 4.5. Computer-Assisted Sperm Analysis (CASA)

Motility parameters for Non-cap and IVC sperm samples were analyzed using the CASA system integrated within NIS-Elements Ar 4.50 (Laboratory Imaging Ltd., Prague, Czech Republic; https://www.nis-elements.cz/en/niselements/nis_advanced_research; accessed on 1 June 2024). Non-cap samples were evaluated at 0 h and IVC samples at 0, 1, 2, and 3 h. The analysis used a phase-contrast microscope Nikon Eclipse E600 (Nikon) equipped with a heating plate, a negative phase-contrast objective, and digital camera DMK 23UM021 (Imaging Source, Bremen, Germany) capturing 60 FPS (frames per second). Sperm suspension (10 μL) was placed in a preheated (38 °C) Makler chamber. Motility was recorded in seven randomly selected fields, with a minimum of 200 spermatozoa analyzed. The analysis was based on three independent biological replicates (*n* = 3), each consisting of a pooled ejaculate from three boars. Spermatozoa with an average path velocity (VAP) threshold ≥ 15 μm and straightness (STR) ≥ 80% were classified as progressively motile. Common kinematic parameters, such as linearity (LIN), STR, the amplitude of lateral head displacement (ALH), straight-line velocity (VSL), VAP, and curvilinear line velocity (VCL), resulted from the analyzed recordings and were further analyzed.

### 4.6. Sperm Bioenergetics and Mitochondrial Complexes Detection

OXPHOS activity was analyzed in Non-cap, and IVC spermatozoa from four individual boars (*n* = 4) in each group. CMs were aliquoted and equilibrated in 5% CO_2_ for ≥1 h; pH was adjusted to 7.5–7.7. Ejaculates (15 mL) were washed three times with PBS (300× *g*, 5 min, RT) (Non-cap group) and resuspended in respective CMs (Cap groups) to 5 × 10^7^ sperm/mL. Oxygen consumption was measured using an Oroboros Oxygraph-2k sv module at 0, 1, 2, and 3 h of incubation (37 °C, 5% CO_2_). Sensors were air-calibrated before each run, 0.5 mL aliquots of sperm suspensions in respective CM were analyzed in duplicate devices. Routine respiration, LEAK state after ATP synthase inhibition by oligomycin (0.2 µM), and maximal respiratory capacity of the electron transport system (ETS, after Carbonyl cyanide p-trifluoromethoxyphenyl-hydrazone; FCCP; 0.5–1.5 µM) were recorded, followed by 0.2 µM rotenone and 0.2 µM antimycin A inhibition to determine residual oxygen consumption rate, which was subtracted as background.

Blue Native-Polyacrylamide gel electrophoresis (BN-PAGE) samples were obtained by solubilizing frozen centrifuged sperm cell pellets (10^9^ cells) by mild detergent digitonin (6 g detergent/1 g protein) in 100 µL solubilization buffer (50 mM NaCl, 50 mM imidazole/HCl, 2 mM 6-aminocaproic acid, 1 mM ethylenediaminetetraacetic acid—EDTA; pH 7.0) supplemented with dithiothreitol (DTT; 10 mM). Samples (30 µg of protein) were separated on a 5–13% polyacrylamide gradient gel using the Mini-PROTEAN III apparatus (Bio-Rad, Hercules, CA, USA) using an imidazole buffer system as described [89].

Proteins separated on BN-PAGE were then transferred onto polyvinylidene difluoride (PVDF) membranes (Immobilon FL 0.45 µm, Merck Millipore, Burlington, MA, USA) by semi-dry electroblotting (0.8 mA/cm^2^, 1.5 h) using a Transblot SD apparatus (Bio-Rad). Proteins were detected by primary antibodies [anti-COX1 (cIV; #ab14705, Abcam, Cambridge, UK) 1:1000, anti-COX6B1 (cIV; #11425-1-AP, Proteintech, Rosemont, IL, USA) 1:500, anti-COX6B2 (cIV; #11437-1-AP, Proteintech) 1:500, anti-NDUFB8 (cI; #ab110242, Abcam) 1:2000, anti-ATP5F1B (cV; #ab14730, Abcam)] 1:2000, followed by fluorescently tagged secondary antibodies (Donkey anti-Mouse IgG (H+L) Highly Cross-Adsorbed Secondary Antibody, Alexa Fluor™ 680 and Donkey anti-Rabbit IgG (H+L) Highly Cross-Adsorbed Secondary Antibody, Alexa Fluor™ 680 (#A10038 and #A10043, respectively, Thermo Fisher Scientific) both 1:3000, and IRDye 800CW Donkey anti-Mouse IgG or IRDye 800CW Donkey anti-Rabbit IgG (#926-32212, #926-32213, Li-COR, Lincoln, NE, USA) both 1:15000. Membranes were scanned using the Odyssey instrument (Li-COR).

### 4.7. Fluorescent Analysis of PTyr Patterns

Non-cap, and IVC sperm suspensions were washed twice in PBS (300× *g*, 10 min) and fixed in 2% methanol-free formaldehyde (#4235.1, Carl Roth GmbH) containing 2% BSA for 20 min at RT. Suspension (10 µL) was placed on slides, blocked with SuperBlock Blocking Buffer (#37517, Thermo Fisher Scientific) for 30 min, and incubated overnight at 4 °C with mouse anti-phosphotyrosine antibody (clone 4G10; Merck Millipore; 1:300) or an IgG Isotype Control (#31903, Invitrogen; Waltham, MA, USA; 1:1000). After washing with PBS, samples were labeled with Alexa Fluor^®^ 488 secondary antibody (#A11001, Invitrogen; 1:300, 1 h, 4 °C, dark) and Peanut agglutinin (PNA)–rhodamine (#RL-1072-5, Vector Laboratories; 1:1000, 30 min, RT, dark). Slides were mounted with VectaShield + DAPI (#H-1200, Vector Laboratories) and examined under a Nikon Eclipse Ni-U microscope. The analysis was based on three independent biological replicates (*n* = 3), each consisting of a pooled ejaculate from three boars. At least 200 spermatozoa per sample were evaluated.

### 4.8. SDS Gel Electrophoresis and Protein Blotting

Frozen sperm pellets (Non-cap, and IVC) from six independent biological replicates (*n* = 6), each consisting of a pooled ejaculate from three boars were lysed in 100 µL of 2× reducing Laemmli buffer [20% (*v*/*v*) glycerol (#G9012), 4% (*w*/*v*) SDS (Sodium Dodecyl Sulfate; #161-0732), 0.125 M Tris-HCl (pH 6.8; #161-0799), 5% (*v*/*v*) β-mercaptoethanol (#M7522), 0.005% (*w*/*v*) bromophenol blue (#B0126)] supplemented with protease/phosphatase inhibitors, incubated on ice for 30 min with stirring, and boiled. Proteins were separated on a 12% polyacrylamide gel using the Precision Plus Protein All Blue Standards (#1610373, Bio-Rad), and transferred (0.5 A, 1 h) to nitrocellulose membranes (#GE10600001, Amersham Protran, GE Healthcare, Chicago, IL, USA). Membranes were blocked with 5% (*w*/*v*) Blotto non-fat dry milk (#SC-2325, ChemCruz, Santa Cruz Biotechnology, Inc., Dallas, TX, USA) in PBS and incubated overnight at 4 °C with anti-phosphotyrosine antibody (clone 4G10; 1:1000) or Mouse IgG Isotype Control (#31903, Invitrogen; 1:10,000). After washing with PBS-T [PBS with 0.1% (*v*/*v*) Tween-20] blots were incubated with horseradish peroxidase (HRP)-conjugated secondary antibody (goat anti-mouse IgG-HRP, Bio-Rad; 1:3000, 1 h, RT), washed, and developed with SuperSignal™ West Pico PLUS substrate (#34580, Thermo Fisher Scientific). Signals were visualized using an Azure C300 system (Azure Biosystems, Dublin, CA, USA) and quantified with IMAGE Studio Digits 3.1 (Li-COR).

### 4.9. Sperm Phosphoproteome Determination

Phosphorylated proteins were isolated from three independent biological replicates (*n* = 3), each consisting of a pooled ejaculate from three boars using the Pierce^®^ Phosphoprotein Enrichment Kit (#90003, Thermo Fisher Scientific). Non-cap and IVC sperm samples were washed twice in 50 mM Hepes buffer (pH 7.0) and lysed in 1 mL of Lysis/Binding/Wash Buffer containing 3-((3-cholamidopropyl) dimethylammonio)-1-propanesulfonate (CHAPS) and protease/phosphatase inhibitors. The subsequent procedure was carried out according to the manufacturer’s protocol. Isolated proteins were analyzed at the Core Facility of Structural Mass Spectrometry (IBT, CAS, BIOCEV) using an Agilent 1200 LC system coupled to a timsTOF SCP mass spectrometer (Bruker Daltonics, Billerica, MA, USA). Data were acquired in data-dependent mode and searched against the *Sus scrofa* database using MaxQuant.

### 4.10. Data and Statistical Analysis

For statistical analysis, GraphPad Prism 9.0 software (GraphPad Software, Boston, MA, USA) was used. Two-way ANOVA, after prior testing of normal distribution using the Shapiro–Wilk test, was used for statistical analysis of sperm capacitation status determined using CTC, CASA, fluorescent analysis of PTyr patterns, phosphoprotein immunodetection, LFQ Intensity of MS-identified proteins, and PKA activity assessment, with the level of statistical significance for the analyses set at *p* < 0.05. Tukey’s multiple comparisons test was used to evaluate statistical relevance.

Principal Component Analysis (PCA) and k-means cluster analysis were used to identify subpopulations of motile sperm during incubation in CMs. The values of all individual CASA parameters ALH, beat cross frequency (BCF), LIN, STR, VAP, VCL, VSL, and wobble (WOB) of motile sperm (VAP ≥15) from all measurements were entered into GraphPad, where PCA was performed to evaluate inter-parameter relationships and to identify variables contributing most strongly to the major sources of variance. High correlations were detected between the parameters LIN and STR (r = 0.881), VSL and VAP (r = 0.915), LIN and VSL (r = 0.759), and STR and WOB (r = 0.881). The variables with high absolute loadings on PC1 were VSL (−0.956), LIN (−0.882), STR (-0.854), VAP (−0.840), and BCF (−0.497). The variables with high absolute loadings on PC2 were ALH (0.744) and VCL (0.837). Because several CASA parameters were highly correlated and therefore represented overlapping aspects of sperm movement, only a subset of non-redundant variables was retained for clustering [22]. Subsequently, a k-means cluster analysis was performed in Excel, for which only the parameters VSL, STR, ALH, and BCF were retained. The parameters LIN (strongly correlated with VSL and STR), VAP (strongly correlated with VSL), and WOB (strongly correlated with STR and VAP) were excluded for the k-means cluster. K-means clustering was applied to the standardized data (Z-score) using three clusters (k = 3), with 40 replicates and 50 iterations per run. The clustering results were visualized and further analyzed to compare motility characteristics among treatment groups and incubation times.

## 5. Conclusions

**Figure 10:**
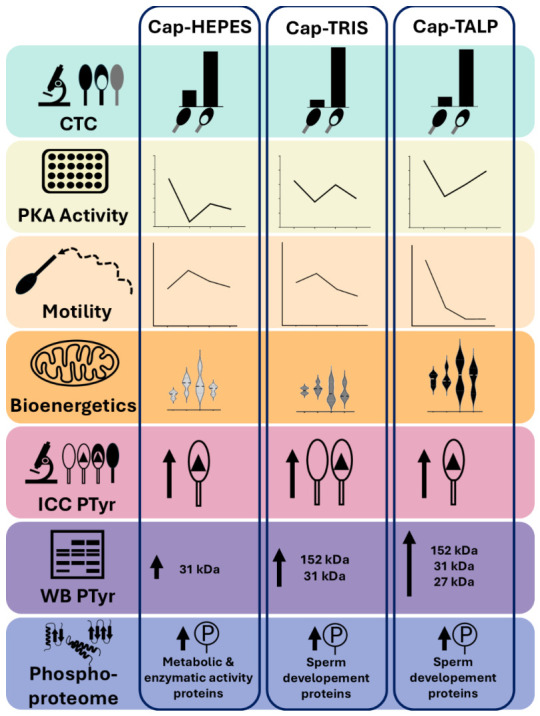
Comparative infographic summarizing the effects of Hepes-based, Tris-based and TALP-based capacitation media.

Our results demonstrate that the CM composition significantly influences both molecular and functional aspects of sperm capacitation (summary in Figure 10). Although all tested media induced capacitation-associated changes, they differed in PKA activity, tyrosine phosphorylation patterns, and motility profiles, indicating that distinct signaling dynamics are triggered depending on medium composition. Among the tested media, the TALP-based CM appears to provide the most physiologically relevant environment for capacitation-associated signaling. However, its reduced motility likely reflects the absence of oviductal or cumulus-derived cues that normally modulate sperm function in vivo. These findings underline the need for more reliable and standardized approaches to evaluate capacitation. Future studies will therefore employ a single, standardized medium and assess boar sperm responses using validated molecular biomarkers to improve analytical reproducibility. Moreover, our findings emphasize the complex regulation of energy metabolism during sperm capacitation. In summary, rather than proposing a universal protocol, this study highlights the necessity of refining and standardization existing formulations to balance molecular activation and functional competence of boar spermatozoa. These findings emphasize the importance of carefully selecting and characterizing in vitro capacitation conditions and providing a basis for new scientific lines for further research.

## Figures and Tables

**Table 1:** Table summarizing proteins with significant differences between sperm groups in Label-free quantification (LFQ) Intensity. Function in biological process and localization of the identified proteins were classified based on annotations available in the protein database UniProt; significant differences are indicated by letters above the average LFQ Intensity values; *n* = 3 independent biological replicates, each replicate consisted of a pooled ejaculate from three boars.

Protein IDs	Protein Names	Gene Names	MW [kDa]	Biological Process (UniProt)	Cell Localization (UniProt)	Mean LFQ Intensity Non-Cap	Mean LFQ Intensity Cap-HEPES	Mean LFQ Intensity Cap-TRIS	Mean LFQ Intensity Cap-TALP
Q4R0H3	Carbohydrate-binding protein AQN-1	AQN-1	14	Binding of sperm to ZP	Cell membrane	13,913,433 ^a^	5,310,900 ^b^	12,204,300 ^c^	16,042,733 ^d^
Q4R0H8	Carbohydrate-binding protein AWN	AWN	17	Binding of sperm to ZP	Cell membrane, Acr.	17,324,000 ^a^	11,109,200 ^b^	17,913,000 ^ca^	22,636,333 ^d^
C8C4M8	Zona pellucida binding protein 2	ZPBP2	37	Binding of sperm to ZP	Acr.	3,340,633 ^a^	10,031,567 ^b^	10,125,067 ^b^	7,610,333 ^b^
Q29108	Zona pellucida-binding protein 1	ZPBP	40	Binding of sperm to ZP	Cell membrane, Acr.	5,189,100 ^a^	8,315,167 ^b^	7,882,000 ^b^	7,532,033 ^b^
A0A5G2QZP6	Zonadhesin	ZAN	286	Binding of sperm to ZP	Cell membrane	4,411,667 ^a^	9,777,033 ^c^	11,108,367 ^c^	10,411,400 ^c^
F1SS24	Fibronectin	FN1	270	Cell adhesion	ECM	191,253,337 ^a^	10,642,967 ^b^	15,565,333 ^c^	17,500,000 ^d^
A0A287AVU8	Jacalin-type lectin domain-containing protein	LOC110259943	18	Cell adhesion	ECM	27,250,333 ^a^	26,188,667 ^b^	33,527,333 ^a^	35,689,333 ^c^
A0A8W4FBB9	Milk fat globule EGF and factor V/VIII domain containing	MFGE8	43	Cell adhesion	Cell membrane	3,394,400 ^a^	8,257,000 ^b^	6,828,200 ^b^	8,497,100 ^c^
A0A287BCE6	VWFD domain-containing protein	FCGBP	271	Cell adhesion	Cell membrane	6,712,167 ^a^	4,734,800 ^b^	4,948,300 ^ab^	4,057,800 ^ab^
A0A287AFN9	Acrosin	ACR	45	Fertilization	Acr.	12,646,000 ^a^	28,854,333 ^b^	19,221,000 ^c^	16,225,000 ^c^
P35495	Major seminal plasma glycoprotein PSP-I	PSP-I	14	Fertilization	ECM	5,537,067 ^a^	2,705,627 ^a^	4,221,167 ^a^	7,637,467 ^b^
A0A8D0TH99	Membrane cofactor protein	LOC100736569	49	Fertilization	Cell membrane	10,630,600 ^a^	13,612,667 ^ab^	12,671,333 ^c^	9,002,667 ^b^
A0A8W4FCQ2	Spermadhesin AQN-3-like protein	SPMI	15	Fertilization	ECM	5,909,267 ^a^	5,645,067 ^a^	14,385,667 ^b^	22,428,667 ^c^
Q866A8	Disintegrin and metalloproteinase domain-containing protein 2	FTNB	82	Gamete adhesion & fusion	Cell membrane	381,997 ^a^	1,658,850 ^ab^	3,905,000 ^ab^	6,005,800 ^b^
Q8MI02	Hyaluronidase	SPAM1	56	Gamete adhesion & fusion	Cell membrane	8,713,267 ^a^	16,429,333 ^b^	15,817,000 ^c^	13,682,000 ^b^
A0A4X1TEC5	Sperm acrosome membrane-associated protein 3	SPACA3	18	Gamete adhesion & fusion	Cell membrane, Acr.	5,453,400 ^ab^	2,550,763 ^a^	4,694,667 ^ab^	4,424,900 ^b^
A0A4X1T5L6	Sperm equatorial segment protein 1	SPESP1	40	Gamete adhesion & fusion	Acr.	2,371,967 ^a^	1,508,350 ^a^	2,693,100 ^ab^	3,410,000 ^b^
A0A287AEA5	Aldehyde dehydrogenase 16 family member A1	ALDH16A1	91	Metabolism & enzymatic activity	Cytoplasm	0 ^a^	15,734,333 ^b^	0 ^a^	0 ^a^
A0A287BPT0	Amine oxidase	IL4I1	64	Metabolism & enzymatic activity	Acr.	3,759,233 ^a^	5,846,033 ^b^	5,154,667 ^b^	6,110,167 ^c^
A0A5G2RC85	Angiotensin-converting enzyme	LOC100515049	88	Metabolism & enzymatic activity	Cell membrane	257,867 ^a^	890,653 ^a^	1,585,783 ^a^	7,546,340 ^b^
A0A286ZMB2	Folate gamma-glutamyl hydrolase	GGH	35	Metabolism & enzymatic activity	ECM	1,819,133 ^a^	3,965,633 ^ab^	5,241,933 ^b^	4,763,300 ^b^
A0A287AQK7	Heat shock protein HSP 90-alpha	HSP90AA1	84	Signal transduction & regulation	Cytoplasm	9,251,200 ^a^	5,354,133 ^b^	7,199,200 ^a^	6,568,367 ^a^
A0A4X1T135	Leucine zipper protein 2	LUZP2	39	Signal transduction & regulation	ECM	17,539,667 ^a^	7,977,600 ^b^	8,129,333 ^b^	12,969,667 ^a^
A0A287B9V6	Leucine-rich repeat-containing protein 37A2	LOC100626147	164	Signal transduction & regulation	Cell membrane	4,885,267 ^a^	7,990,533 ^b^	6,719,267 ^ab^	5,239,567 ^ab^
I3LK18	Polycystin-2-like protein 2	PKD2L2	74	Signal transduction & regulation	Cell membrane	1,591,277 ^a^	3,486,667 ^ab^	4,520,633 ^b^	4,638,700 ^b^
Q29016	Acrosin-binding protein	ACRBP	61	Spermatogenesis & sperm development	Acr.	8,631,767 ^a^	14,562,233 ^b^	10,741,633 ^b^	11,298,600 ^b^
F1RRW5	Angiotensin-converting enzyme	ACE	150	Spermatogenesis & sperm development	Cell membrane and Cytoplasm	4,079,767 ^ab^	2,617,100 ^a^	3,341,667 ^ab^	4,480,367 ^b^
F1S1R1	Cylicin 1	CYLC1	74	Spermatogenesis & sperm development	Cytoplasm, PT, Acr.	22,337,333 ^a^	16,183,000 ^b^	17,085,667 ^a^	13,902,333 ^b^
A0A5K1TZB6	Heterogenous nuclear ribonucleoprotein H3	HNRNPH3	35	Spermatogenesis & sperm development	Nucleus	0 ^a^	0 ^a^	10,189,000 ^b^	4,441,000 ^ab^
D5K8A9	Sperm acrosome membrane-associated protein 1	SPACA1	32	Spermatogenesis & sperm development	Cell membrane, Acr.	4,681,567 ^a^	8,573,900 ^b^	7,546,300 ^b^	6,941,167 ^b^
A0A5G2QGK1	Tubulin beta chain	TUBB4B	50	Spermatogenesis & sperm development	Cytoplasm	2,454,967 ^a^	2,798,100 ^a^	3,500,700 ^ab^	4,528,900 ^b^

Acr.—acrosome; ECM—extracellular matrix; PT—perinuclear theca; ZP—zona pellucida.

**Table 2:** Capacitation media composition.

Hepes-Based CM (pH 7.2–7.4; 310 mOsm/L) [22]	Tris-Based CM (pH 7.2–7.4; 290 mOsm/L) [31]	TALP-Based CM (pH 7.4; 355 mOsm/L) [30]
114 mM NaCl (#SZBC1650V)	113 mM NaCl	114 mM NaCl
3.2 mM KCl (#P-5405)	0.3 mM KCl	3.2 mM KCl
0.34 mM NaH_2_PO_4_ (#S9638)	-	0.35 mM NaH_2_PO_4_
10 mM Na-Lactate (#L4263)	-	18 mM Na-Lactate
-	-	8 mM Ca-Lactate (#C8356)
0.5 mM MgCl_2_ (#M2393)	-	0.5 mM MgCl_2_
10 mM Hepes (#H4034)	2 mM Tris (#22H044110)	-
5.2 mM Na-Pyruvate (#P2256)	0.5 mM Na-Pyruvate	1.1 mM Na-Pyruvate
12 mM Sorbitol (#S3889)	-	-
0.05 mM Gentamicin (#G1264)	-	0.17 mM Kanamycin sulfate (#606-15)
0.19 mM Penicillin (#P3032)	-	-
0.01% (*w*/*v*) PVA (#P8136)	-	1% (*w*/*v*) PVA
10.99 mM Glucose (#G-7021)	1.1 mM Glucose	5 mM Glucose
2% (*w*/*v*) BSA (#A9647)	0.1% (*w*/*v*) BSA	0.3% (*w*/*v*) BSA
2 mM NaHCO_3_ (#S5761)	-	25 mM NaHCO_3_
2 mM CaCl_2_ (#C7902)	1.33 mM CaCl_2_	-
-	-	2 mM Caffeine (#C0750)

BSA—bovine serum albumin, PVA—polyvinyl alcohol.

## Data Availability

The original contributions presented in this study are included in the article/Supplementary Materials. Further inquiries can be directed to the corresponding authors.

## Supplementary Materials

The following supporting information can be downloaded at: https://www.mdpi.com/article/10.3390/ijms27104567/s1.

## Abbreviations

The following abbreviations are used in this manuscript:

Acr.	Acrosome
ACRB	Acrosome binding protein
ALDH	Aldehyde dehydrogenase
ALH	Amplitude of lateral head displacement
AQN-1	Carbohydrate binding protein AQN-1
AQN-3	Carbohydrate binding protein AQN-3
ATP	Adenosine triphosphate
AWN	Carbohydrate binding protein AWN
BCF	Beat cross frequency
BN-PAGE	Blue Native Polyacrylamide Gel Electrophoresis
BSA	Bovine serum albumin
cAMP	Cyclic adenosine monophosphate
Cap-HEPES	Spermatozoa capacitated in Hepes-based capacitation medium
Cap-TALP	Spermatozoa capacitated in TALP-based capacitation medium
Cap-TRIS	Spermatozoa capacitated in Tris-based capacitation medium
CASA	Computer Assisted Sperm Analysis
CatSper	Cation channels of sperm
CHAPS	3-((3-cholamidopropyl) dimethylammonio)-1-propanesulfonate
CM	Capacitation medium
COC	Cumulus–oocyte complex
CTC	Chlortetracycline
DAPI	4′,6-diamidino-2-phenylindole
DTT	Dithiotreitol
ECM	Extracellular matrix
EDTA	Ethylenediaminetetraacetic acid
EqSS	Equatorial subsegment
ETS	Electron transport system
FCCP	Carbonyl cyanide p-trifluoromethoxyphenyl-hydrazone
FPS	Frames per second
HRP	Horseradish peroxidase
HVC1	Voltage-gated hydrogen channel 1
IVC	In vitro capacitation
IVF	In vitro fertilization
LFQ	Label-Free Quantification
LIN	Linearity
MS	Mass spectrometry
NBC	Na^+^/HCO_3_^−^ cotransporter
Non-cap	Non-capacitated spermatozoa
OXPHOS	Oxidative phosphorylation
PBS	Phosphate-buffered saline
PBS-T	Phosphate-buffered saline supplemented with Tween
PCA	Principal component analysis
PKA	Protein kinase A
PMOT	Progressive motility
PMSF	Phenylmethylsulfonyl fluoride
PNA	Peanut agglutinin
PSP-I	Major seminal plasma glycoprotein
PT	Perinuclear theca
PTyr	Tyrosine phosphorylation
PVA	Polyvinyl alcohol
PVDF	Polyvinyllidene difluoride
ROD	Relative optical density
RT	Room temperature
sAC	Soluble adenylate cyclase
SDHA	Succinate dehydrogenase complex flavoprotein subunit A
SDS	Sodium dodecyl sulfate
SEM	Standard error of the mean
Ser	Serine
SPACA1	Sperm acrosome-associated protein 1
SPACA3	Sperm acrosome membrane-associated protein 3
SPAM1	Hyaluronidase
STR	Straightness
TALP	Tyrode’s albumin lactate pyruvate medium
Thr	Threonine
TMOT	Total motility
Tyr	Tyrosine
VAP	Average path velocity
VCL	Curvilinear line velocity
VSL	Straight-line velocity
WOB	Wobble
ZP	Zona pellucida
εPKC	Epsilon protein kinase C
