# Supplementary material for: Medium Composition Determines the Dynamics of Boar In Vitro Sperm Capacitation-Associated Events
Source: Int J Mol Sci. 2026 May 19;27(10):4567. doi: 10.3390/ijms27104567 (PMC13206783; doi:10.3390/ijms27104567)
Supplement: Supplementary file 1 [file ijms-27-04567-s001.zip › ijms-4298754-supplementary.pdf]

Medium composition determines the dynamics of boar in vitro sperm capacitation-associated events

Barbora Klusackova[1], Zuzana Pilsova[1], Barbora Bryndova[2,3], Aneta Pilsova[1], Natalie Zelenkova[1], Petr Pecina[4], Michal Knezu[4,5], Petra Secova[6], Pavla Tymich Hegrova[1], Eva Chmelikova[1], Katerina Komrskova[2,7], Ondrej Simonik[2*], Pavla Postlerova[1,2*]

[1] Department of Veterinary Sciences, Faculty of Agrobiology, Food, and Natural Resources, Czech University of Life Sciences Prague, Prague, Czech Republic

[2] Laboratory of Reproductive Biology, Institute of Biotechnology of the Czech Academy of Sciences, BIOCEV, Vestec, Czech Republic

[3] Department of Biochemistry, Faculty of Science, Charles University, Prague, Czech Republic

[4] Laboratory of Bioenergetics, Institute of Physiology, Czech Academy of Sciences, Prague, Czech Republic

[5] Department of Cell Biology, Faculty of Science, Charles University, Prague, Czech Republic

[6] Laboratory of Reproductive Physiology, Institute of Animal Biochemistry and Genetics, Centre of Biosciences, Slovak Academy of Sciences, Bratislava, Slovak Republic

[7] Department of Zoology, Faculty of Science, Charles University, Prague, Czech Republic

Corresponding authors: Ondrej Simonik, email: ondrej.simonik@ibt.cas.cz; Pavla Postlerova, email: postlerova@af.czu.cz; pavla.postlerova@ibt.cas.cz

Supplementary Table S1: Proteins with significant differences between sperm groups in Lower Quantification Limit

Uniprot ID	Gene names	Identified phosphorylation via MS	Predicted Phosphorylation based on FASTA and generic prediction using NetPhos-3.1 (DTU Health Tech)
**A0A287AFN9**	*ACR*	Ser56	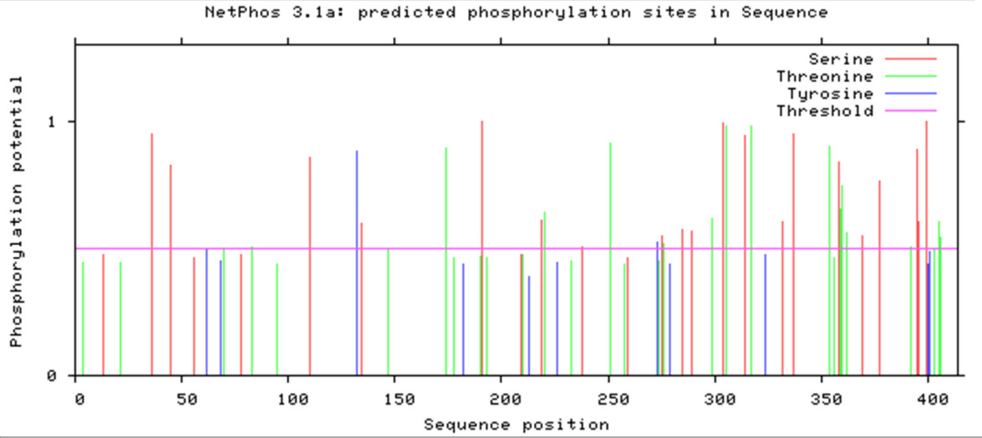<br>%1 ...................................S........S..... # 50<br>%1 ...........Y....................T................. # 100<br>%1 .........S.....................Y.S................ # 150<br>%1 .......................T................S......... # 200<br>%1 ..................ST.................S............ # 250<br>%1 T.....................Y.ST........S...S.........T. # 300<br>%1 ...ST........S..T..............S....S............. # 350<br>%1 ...T...STT.T......S.......S..............T..SS..S. # 400<br>%1 ..T.TT........
**Q29016**	*ACRBP*	-	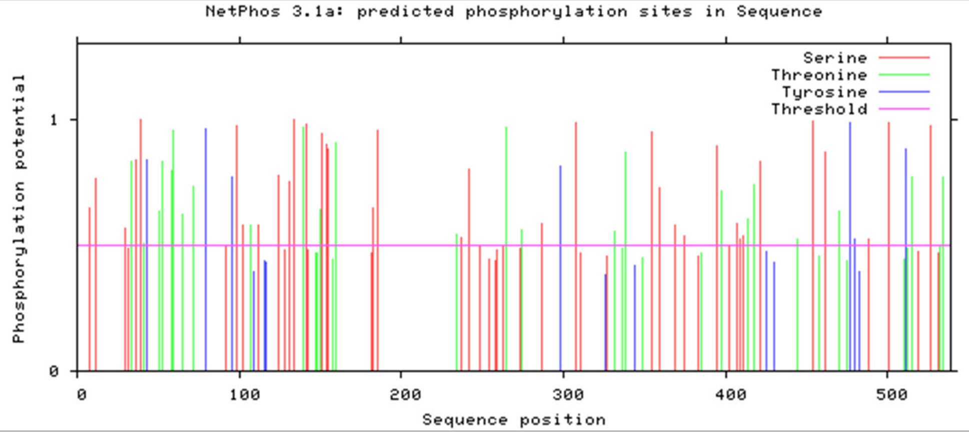<br>%1 .......S..S..................S..T..S..S.T.Y....... # 50<br>%1 T.T....TT.....T......T......Y................Y.S.. # 100<br>%1 .S....T....S...........S......S..S.....TS........T # 150<br>%1 S..SS....T......................S.S............... # 200<br>%1 .................................T..S....S........ # 250<br>%1 ..............T........T............S..........Y.. # 300<br>%1 .......S.......................T.....T............ # 350<br>%1 ....S...S.........S.....S...................S..T.. # 400<br>%1 .S....S.S.S..T...T..S......................T...... # 450<br>%1 ...S.......S.......T......Y..Y.......S............ # 500<br>%1 S.........Y...T...........S......T.....
**A0A287AEA5**	*ALDH16A1*	Ser7; Thr 5	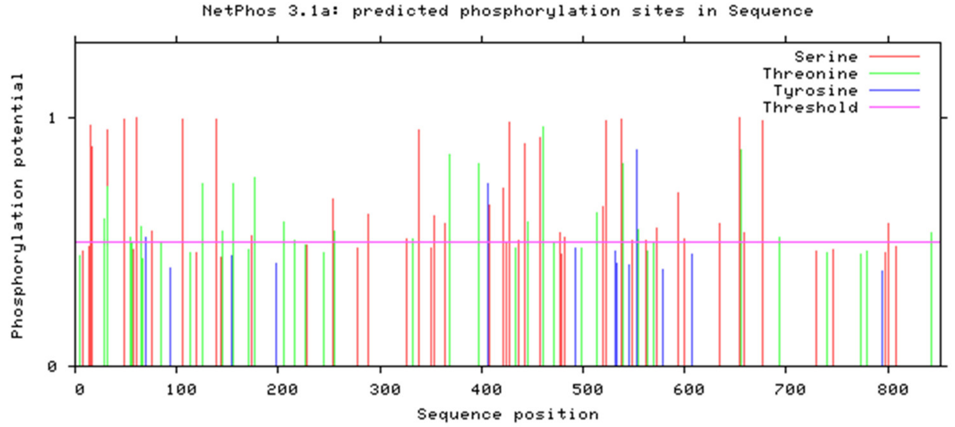<br>%1 ..............S.S..........T..TS................S. # 50<br>%1 ...T......S...T...Y.....S.........T............... # 100<br>%1 .....S...................T...........S.....T..... # 150<br>%1 .....T.................S..T....................... # 200<br>%1 ....T..........T.................................. # 250<br>%1 ...S.T................................S........... # 300<br>%1 ..........................S....T......S........... # 350<br>%1 ..S..........S....T...........................T... # 400<br>%1 .....Y.S.............S....S........S.....S..T..... # 450<br>%1 ......S...T...............S...S................... # 500<br>%1 .............T.....S..S..............S.T.......S.. # 550<br>%1 ..Y.T......S..........S...................SS.....S # 600<br>%1 ....S................ # 650<br>%1 ...ST..S..................S................T...... # 700<br>%1 .... # 750<br>%1 .... # 800<br>%1 S...T....... # 850<br>%1 ..
**A0A287BPT0**	*IL4I1*	-	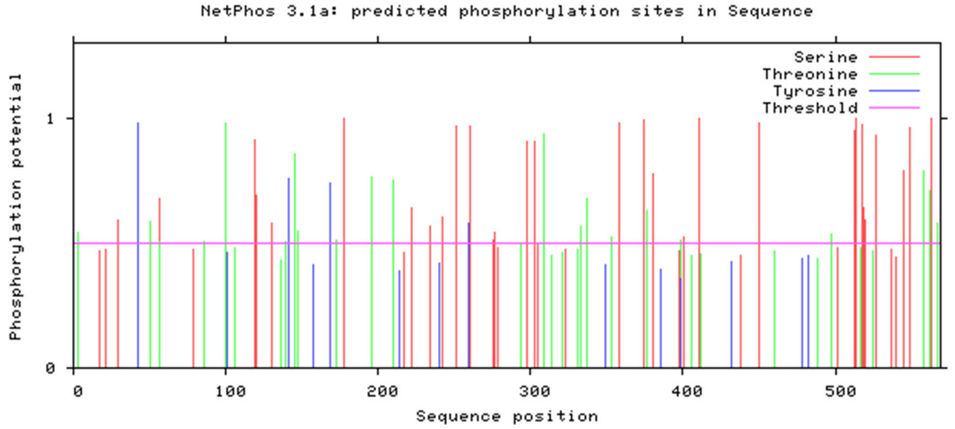<br>%1 ..T.........................S............Y.......T # 50<br>%1 .....TS............................T.............T # 100<br>%1 ..................SS.........S........T.Y...T.T... # 150<br>%1 .................Y....T....S.................T.... # 200<br>%1 .........T...........S...........S.......S........ # 250<br>%1 S.......YS..............SS.....................S.. # 300<br>%1 ..S.....T.......................T...T............. # 350<br>%1 ..T....S...............S.T...S..................T. # 400<br>%1 S.........S......................................S # 450<br>%1 ....T... # 500<br>%1 ............SS...SSS......S.................S...S. # 550<br>%1 .......T...TS...T..

A0A5G2RC85 | *LOC100515049* | -

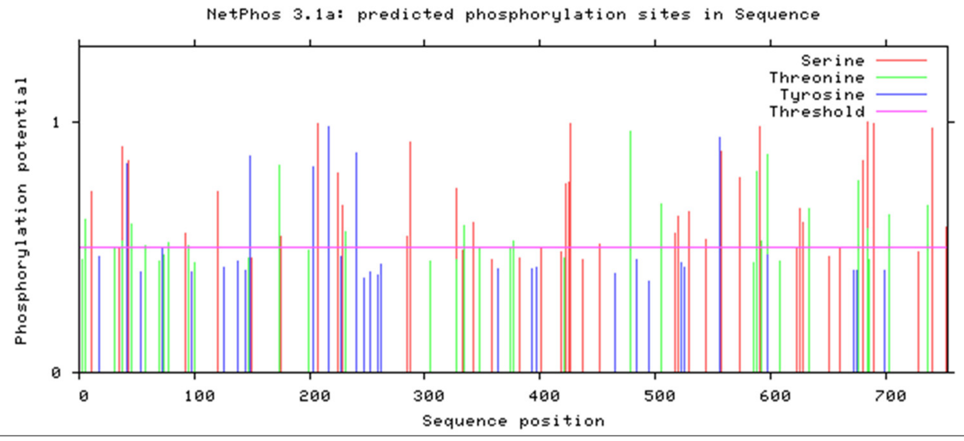


```
%1  .....T....S.........................ST...YS.T.....  #    50
%1  ......T...................T..............S..T.....  #   100
%1  ...................S...........................TY.  #   150
%1  .......................TS.........................  #   200
%1  ..Y...S........Y........S...S.T........Y..........  #   250
%1  ..................................S..S............  #   300
%1  ...........................S.....T.......S........  #   350
%1  ..........................T.......................  #   400
%1  S.....................S.SS........................  #   450
%1  .S.........................T......................  #   500
%1  ....T............S.S........S..............S......  #   550
%1  .....Y.S..............SS.............T..ST....T...  #   600
%1  ........................S...S....T................  #   650
%1  .........................T....S..TS....S..........  #   700
%1  ..T.................................T..S..........  #   750
%1  .S..
```

F1RRW5 | *ACE* | Ser1302

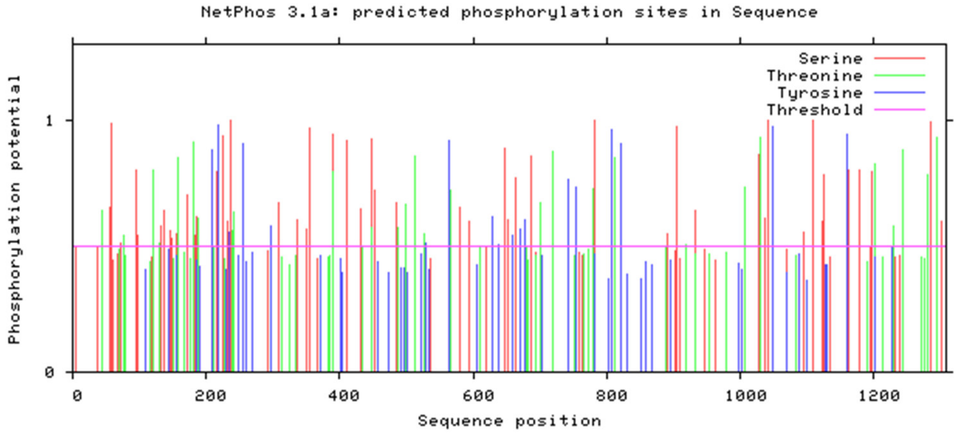


```
%1  ...........................................T......  #    50
%1  .....S..S............S....T................S...S..  #   100
%1  ...................T.........T..S...S........S..S.  #   150
%1  .....ST.............S...........TS.S.T............  #   200
%1  .......Y......S...Y.....S.......S.Y.S.T.T.........  #   250
%1  ....Y..........................................Y..  #   300
%1  ........S..........................S.............S  #   350
%1  .....S..................................ST........  #   400
%1  ..........S...................S................TS.  #   450
%1  ..S..............................S..T............T  #   500
%1  .............T.............T.Y....................  #   550
%1  ............Y.ST.............SS............S......  #   600
%1  ............................Y.........Y.......S...  #   650
%1  ..S.....Y...S......Y......Y........S.............T  #   700
%1  ..................T.......................Y.......  #   750
%1  ...Y........................TS.S..................  #   800
%1  .......Y...T........Y.............................  #   850
%1  ........................................T.S.......  #   900
%1  ....S.............T.............S.................  #   950
%1  ..................................................  #  1000
%1  .......T...................S.ST.....S.....S....Y..  #  1050
%1  ............................................S.....  #  1100
%1  .........S.............SS.........................  #  1150
%1  ..........Y.S...............S................S.S..  #  1200
%1  .T...........................T............T.......  #  1250
%1  .............................T....S........T......  #  1300
%1  .S......S
```

Q4R0H3 | *AQN-1* | -

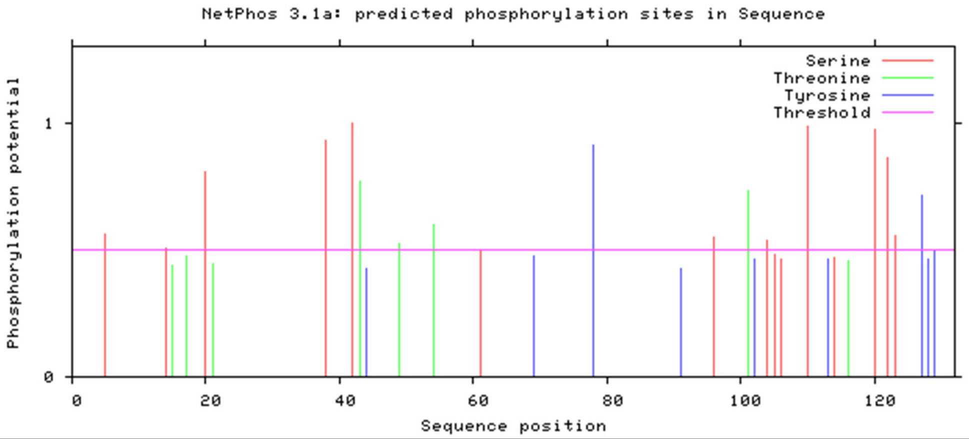


```
%1  ....S........S.....S.................S...ST.....T.  #    50
%1  ...T.......................Y.................S....  #   100
%1  T..S.....S.........S.SS...Y.....
```

F1S1R1 | *CYLC1* | Ser297; Ser339; Ser371; Ser371; Ser376; Ser408; Ser413; Ser416; Ser445; Ser450; Ser452; Ser478; Ser485; Ser490; Ser497; Ser519; Ser523; Ser544; Ser547; Thr409; Thr446; Ser486

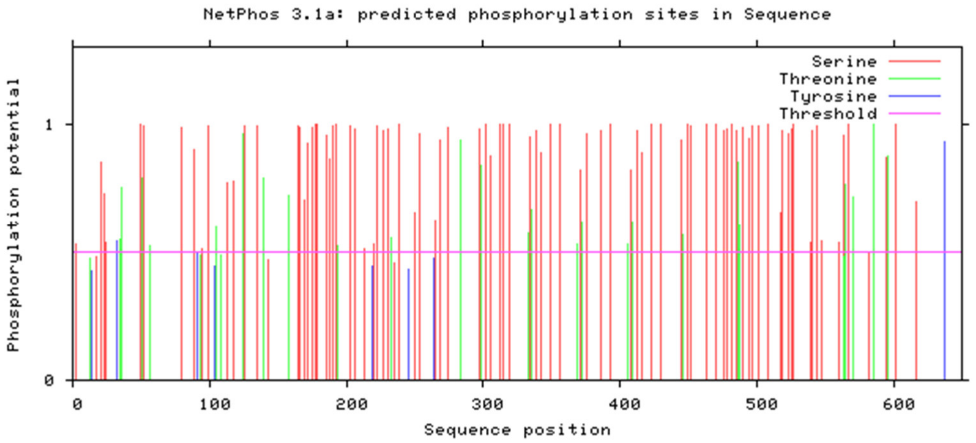


```
%1  .S..................S.SS.......Y.T.T............S.  #    50
%1  TS....T......................S........S.....S...S.  #   100
%1  ....T.......S....S.....TTS........S...T...........  #   150
%1  .......T......SS..S..S..S.S.S.....S..S.S.S.T......  #   200
%1  ..S..S......S......S.SS...S...S.T.....S..........S  #   250
%1  ...S..........S...S....S.........T............ST..  #   300
%1  .S..S......S..S...S.............TST...S..S......S.  #   350
%1  .....S............T.ST...S.........S......S.......  #   400
%1  .....T.ST...S..S......S......S..............ST...S  #   450
%1  .S..........S......S.....S.S...S..ST.T.S...S..S...  #   500
%1  S......S.........SS...S..SS...........SS...S..S...  #   550
%1  .........S...ST.S...T.............TT........ST....  #   600
%1  .S..............S...................Y.............  #   650
```

<table>
<tr>
<td>Q866A8</td>
<td>FTNB</td>
<td>-</td>
<td>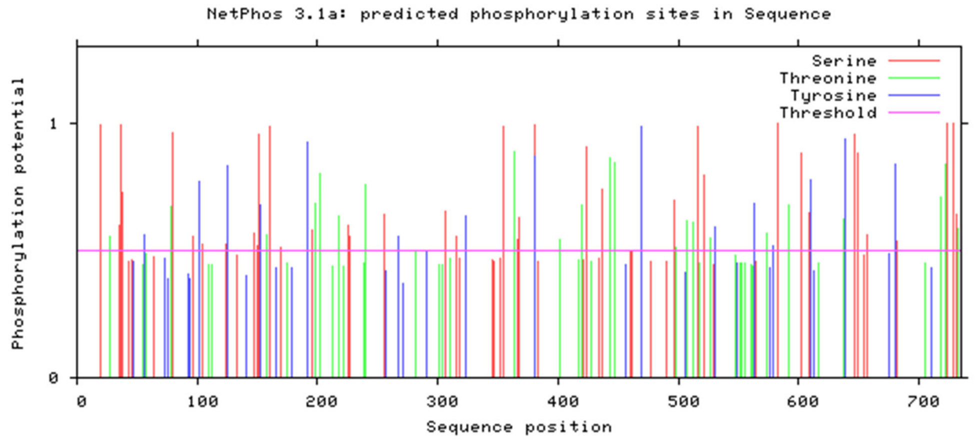
</td>
<td>%1 ..................S........T......S.SS............ # 50
%1 .....Y.....................T.S................S... # 100
%1 Y..S...................SY.....................S..S # 150
%1 S.Y....T.S........S.....................Y....S.T.. # 200
%1 .T..............TS.......SS............T.......... # 250
%1 .....S..........Y................................. # 300
%1 .....S.........S......Y........................... # 350
%1 ....S.......T..S.S...........SY................... # 400
%1 T.................T....S...........S......T...T... # 450
%1 ..................Y..........................S.T.. # 500
%1 ......T....T...S....S.....T...Y................... # 550
%1 ............Y.........T....Y....S........T........ # 600
%1 .S......SY..........................TY.......S..S. # 650
%1 ......S......................Y.S.................. # 700
%1 .................T...TS.....S.S.T..</td>
</tr>
<tr>
<td>F1SS24</td>
<td>FN1</td>
<td>Thr214</td>
<td>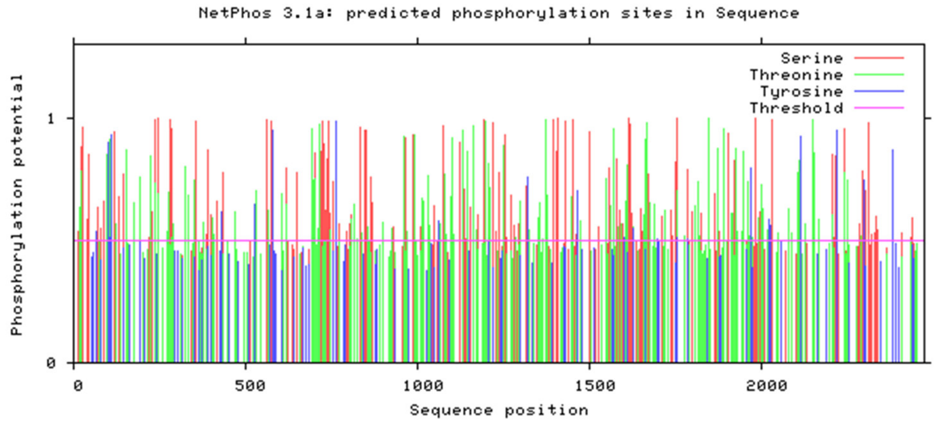
</td>
<td>%1 ..............S..TT..ST..S.S...........S....S..... # 50
%1 .................Y..S.........S......S......T....Y # 100
%1 ...TY....TY.....S.....T.........S............S.... # 150
%1 .T........................T...............T....... # 200
%1 ...................S.....TS..........TS.....T.S... # 250
%1 .......................TS..T.S..S.S.T...T......... # 300
%1 ........................T........T...............T # 350
%1 ....S.........T..........T..........S..S........S. # 400
%1 ....T....T....S...............Y....S.............. # 450
%1 ....T............................. # 500
%1 ...........S............Y....T.................... # 550
%1 .............S...T......S...Y..................... # 600
%1 ...T...T..........T.S............................S # 650
%1 ....T....T.T # 700
%1 STSS.....T....TT..S.....S.S.T....SS...S..S...T.S.. # 750
%1 ......S.......Y.......TS...................S...... # 800
%1 ...S.S.T.........T......T......S......T......S.S.. # 850
%1 .SST.........S...S..............................T. # 900
%1 ...........................T...S.................. # 950
%1 .........T.......S...T.............S.......T...... # 1000
%1 ..........ST.....T...........T...T..............S. # 1050
%1 ...........Y..T.........S...T...T............T.... # 1100
%1 TT.....T.............S.........TS...S...S..T...... # 1150
%1 ...S.........T.......T..S..................S....TT # 1200
%1 ........TTT.T......S.........S.......S..........T. # 1250
%1 .....S...S.............S.......S.....T....S....... # 1300
%1 T................SS..YY..T.......................T # 1350
%1 T.T..T..........T.....T...T................S...... # 1400
%1 .....S.S......................S...............T... # 1450
%1 S............S...Y......T.T......................S # 1500
%1 ..S.......................S......................T # 1550
%1 ......S......T......T.......S.....T...S..T.T.S.... # 1600
%1 ...Y..T....T....S..SS...S..Y.T..................S. # 1650
%1 .....SS..T....TTT....S..S...T........T.........Y.. # 1700
%1 ..........S........T................S......S.....S # 1750
%1 ....TYSS.................T.........S.Y............ # 1800
%1 S.....T........T.......T..S...................T... # 1850
%1 ...............S.........T....S........TS.......T. # 1900
%1 ....S.......T....TT...S..T.T.T..............T....T # 1950
%1 ..........T.....T.Y.....T.......S.......T.....S... # 2000
%1 ...TT...............T.Y...Y....S................T. # 2050
%1 T.............................T.......T........... # 2100
%1 .....T...T.......Y..........T....................T # 2150
%1 T.....T.........T................................. # 2200
%1 ......S..T.S......S..Y....................TS.....T # 2250
%1 ......................................S..T..S....Y # 2300
%1 ..S...........S......S.......S......SS............ # 2350
%1 .................................T.Y.............. # 2400
%1 .......S..........................S...S........... # 2450
%1 ....S..</td>
</tr>
<tr>
<td>A0A286ZMB2</td>
<td>GGH</td>
<td>-</td>
<td>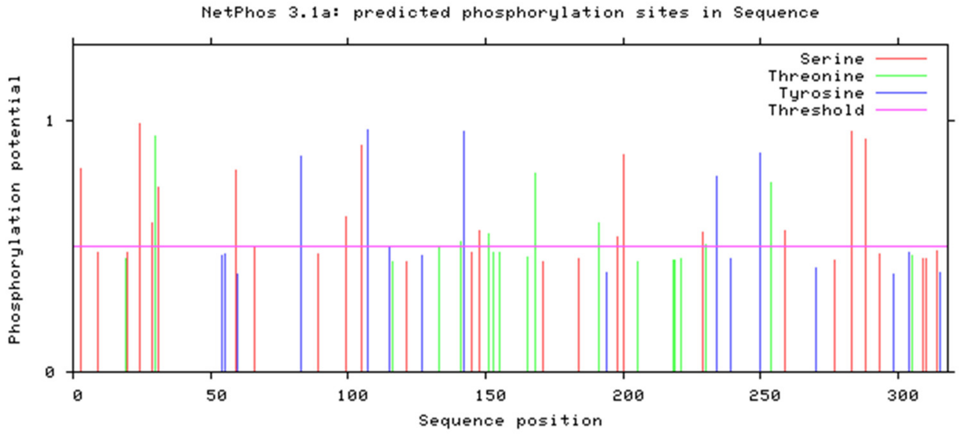
</td>
<td>%1 ..S....................S....STS................... # 50
%1 ........S.......................Y...............S. # 100
%1 ....S.Y.................................TY.....S.. # 150
%1 T................T......................T......S.S # 200
%1 ............................ST...Y...............Y # 250
%1 ...T....S.......................S....S............ # 300
%1 ....</td>
</tr>
</table>

<table>
<tr><td>A0A287AQK7</td><td>HSP90AA1</td><td>Ser260; Ser263;
Ser228; Ser231</td><td>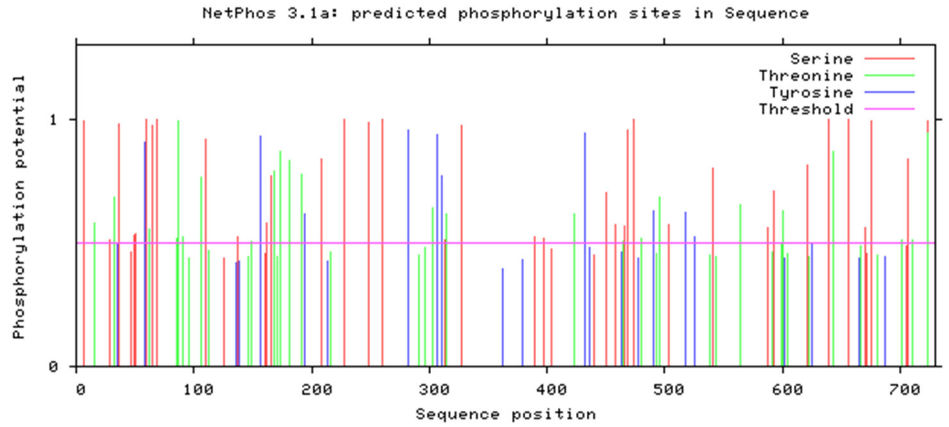
</td><td>%1 .....S.........T...........S....T.YS............SS # 50
%1 .......Y.S.T..S...S...............T.T...T......... # 100
%1 .....T...S..........................S...........T. # 150
%1 ......Y....S...S.T....T.......T..........T.Y...... # 200
%1 .......S...................S....................S. # 250
%1 .........S.....................Y.................. # 300
%1 ..T...Y...Y.S.T............S...................... # 350
%1 ......................................S.......S... # 400
%1 ......................T........Y.................. # 450
%1 S......S......TS.S.....S.....T.........YY....T.... # 500
%1 ..S..............Y.......Y..............S......... # 550
%1 .............T......................S.....S....... # 600
%1 T...................S.................S...T....... # 650
%1 .....S..............S...S......................... # 700
%1 .T....S...T...........TS......</td></tr>
<tr><td>A0A5K1TZB6</td><td>HNRNPH3</td><td>-</td><td>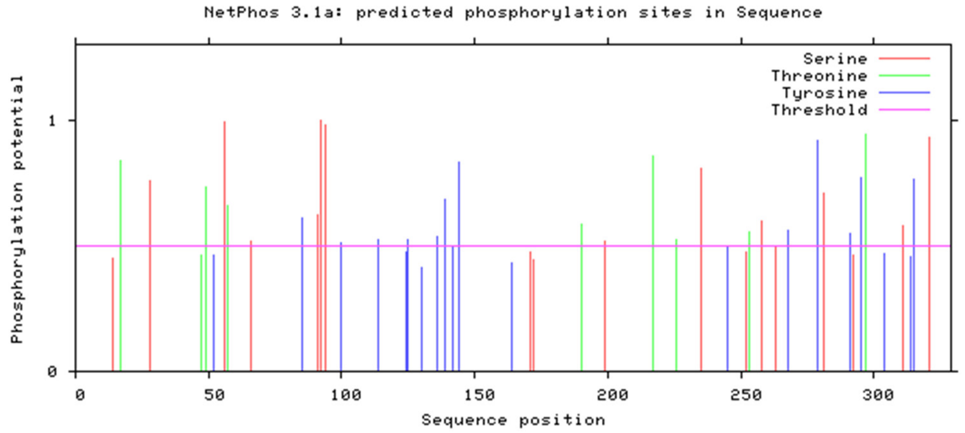
</td><td>%1 ................T..........S....................T. # 50
%1 .....ST........S..................Y.....SS.S.....Y # 100
%1 .............Y..........Y..........Y..Y....Y...... # 150
%1 ....T........S. # 200
%1 ................T........T........S............... # 250
%1 ..T....S.........Y..........Y.S.........Y...Y.T... # 300
%1 ..........S...Y.....S........</td></tr>
<tr><td>Q8MI02</td><td>SPAM1</td><td>Thr360; Thr361;
Thr370; Tyr365</td><td>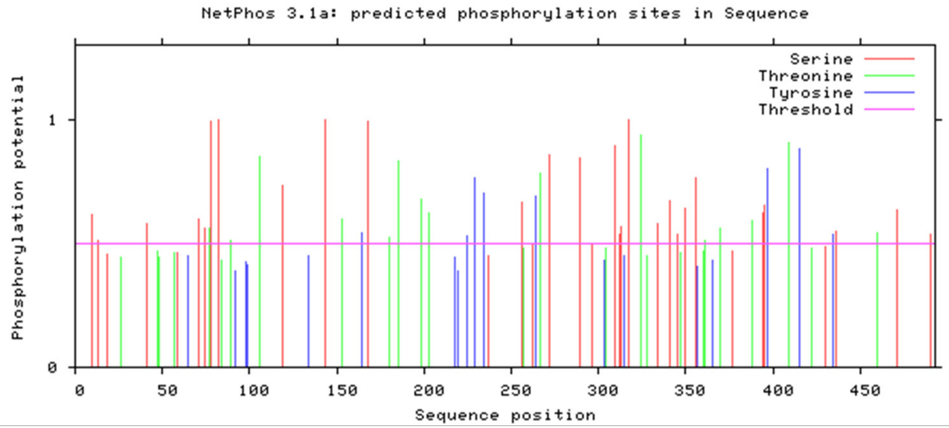
</td><td>%1 .........S..S...........................S......... # 50
%1 ....................S..S..TS...S......T........... # 100
%1 .....T............S.......................S....... # 150
%1 ..T..........Y...S...........T....T............T.. # 200
%1 ..T.....................Y...Y....Y................ # 250
%1 .....S.......Y..T....S................S........... # 300
%1 ........S..SS...S......T.........S......S...S....S # 350
%1 .....S....T........T.................T.....SS.Y... # 400
%1 ........T.....Y..................Y.S.............. # 450
%1 .........T..........S..................S...</td></tr>
<tr><td>A0A287AVU8</td><td>LOC110259943</td><td>-</td><td>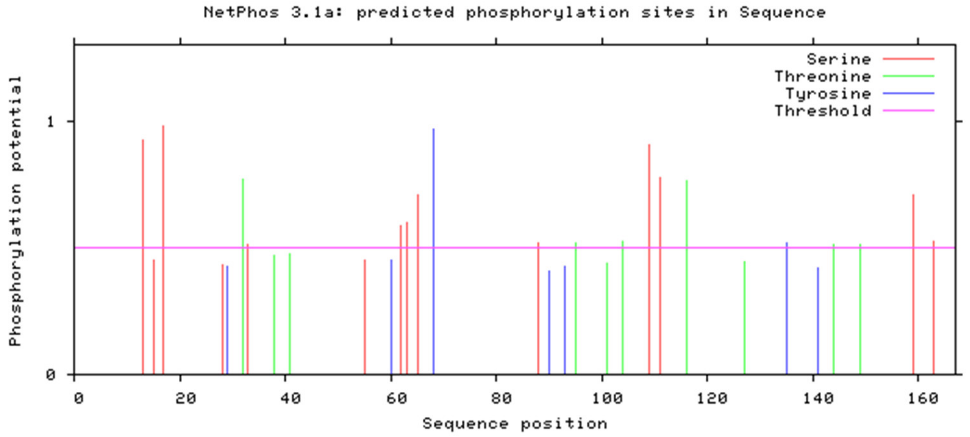
</td><td>%1 ............S...S..............TS................. # 50
%1 ...........SS.S..Y...................S......T..... # 100
%1 ...T....S.S....T..................Y........T....T. # 150
%1 ........S...S....</td></tr>
</table>

**A0A4X1T135**	*LUZP2*	-	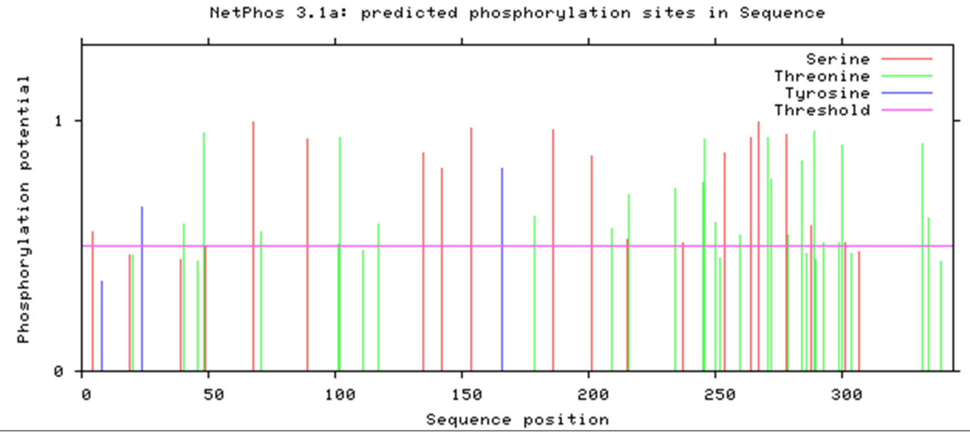	%1 ...S...................Y...............T.......T.. # 50<br>%1 .................S..T.................S........... # 100<br>%1 TT..............T.................S......S........ # 150<br>%1 ...S...........Y............T......S.............. # 200<br>%1 S.......T.....ST.................T..S.......TT...T # 250<br>%1 ...S.....T...S..S...TT.....ST....T...ST...T.....TT # 300<br>%1 S..............................T.T..........
**A0A287B9V6**	*LOC100626147*	-	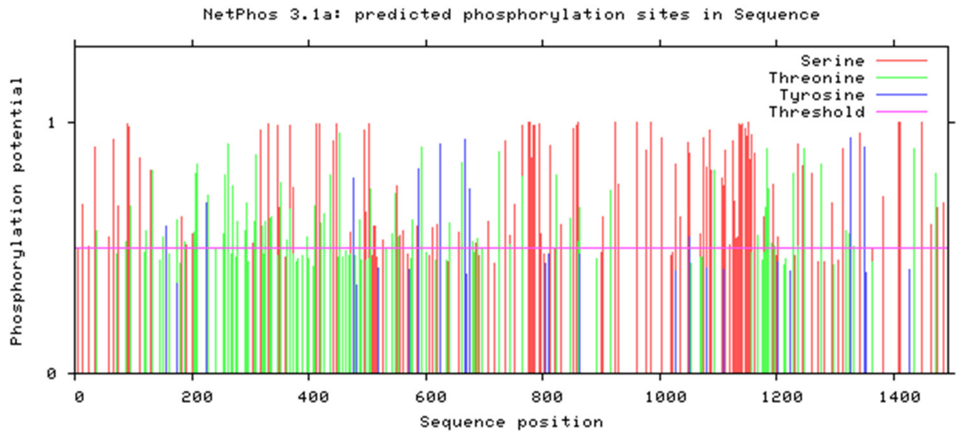	%1 .............S.........S.........S.T.............. # 50<br>%1 ......S.........S......S............T..S.S...T.... # 100<br>%1 ..........S.........T........S.T.................. # 150<br>%1 .T....Y..................T......S....T.S.........S # 200<br>%1 ..T..T...T.............Y.S.T............T......... # 250<br>%1 ....T.T...TT......T........T............T.T....... # 300<br>%1 ....S..T..T.....S..T....T....S.S.T..T..........S.S # 350<br>%1 .T.........TT...ST....S.......................T... # 400<br>%1 .........T.S......S.T...T............T....S...S... # 450<br>%1 ..T.................S....Y..........T.......S..S.. # 500<br>%1 .S...T.....SS.............S...............T....T.S # 550<br>%1 .T..S......S.............T.........SY....T........ # 600<br>%1 ..........S.......S.....Y..............T.......... # 650<br>%1 .....S....TT....Y........Y....T...S.TT............ # 700<br>%1 .....S..................T...........S......TS..... # 750<br>%1 .S...........TS..........SS..S..S.SS........S..S.. # 800<br>%1 ...........S.........T.........S.............T.... # 850<br>%1 .S....S..ST..T.................................... # 900<br>%1 ..S.............T......S.....S.................... # 950<br>%1 .........S.................S.....S................ # 1000<br>%1 ...S......................S......S............S... # 1050<br>%1 SY.................S...S......S...S.S.....TT...... # 1100<br>%1 ......SS..S.......S....S...S.S...SS.S...SS....S..S # 1150<br>%1 .S.S..S.....S...T...........S.T.T...T....T..ST.... # 1200<br>%1 .S.........................T......TS.......S..T... # 1250<br>%1 ........S................T..................S..... # 1300<br>%1 ............S.....T....T..Y....T........SS........ # 1350<br>%1 Y.............................S................... # 1400<br>%1 ......S..S.S.....................T.............SS. # 1450<br>%1 ............S........T..S........S.........
**P35495**	*PSP-I*	-	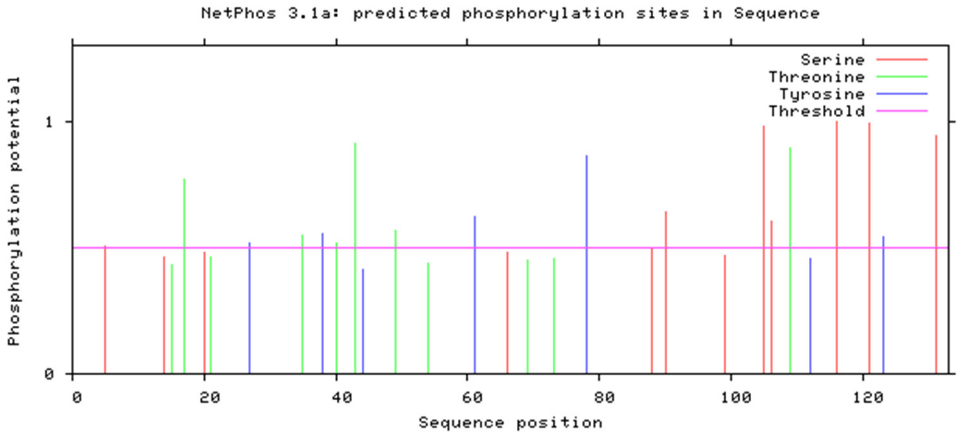	%1 ....S...........T.........Y.......T..Y.T..T.....T. # 50<br>%1 ..........Y................Y...........S.......... # 100<br>%1 ....SS..T......S....S.Y.......S..
**A0A8D0TH99**	*LOC100736569*	Ser413; Ser 422; Ser424; Thr421	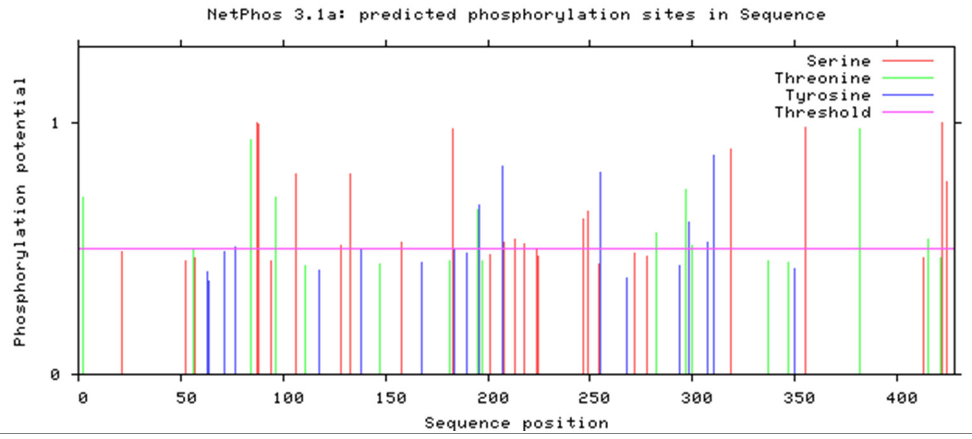	%1 .T.. # 50<br>%1 ..........................Y......T..SS.......T.... # 100<br>%1 .....S.....................S....S....Y............ # 150<br>%1 .......S........................SY..........TY.... # 200<br>%1 ......YS....S....S............................S.S. # 250<br>%1 ....Y..........................T..............TY.T # 300<br>%1 ......Y..Y........S............................... # 350<br>%1 ....S..........................T.................. # 400<br>%1 ..............T......S.S....

**A0A8W4FBB9**	*MFGE8*	-	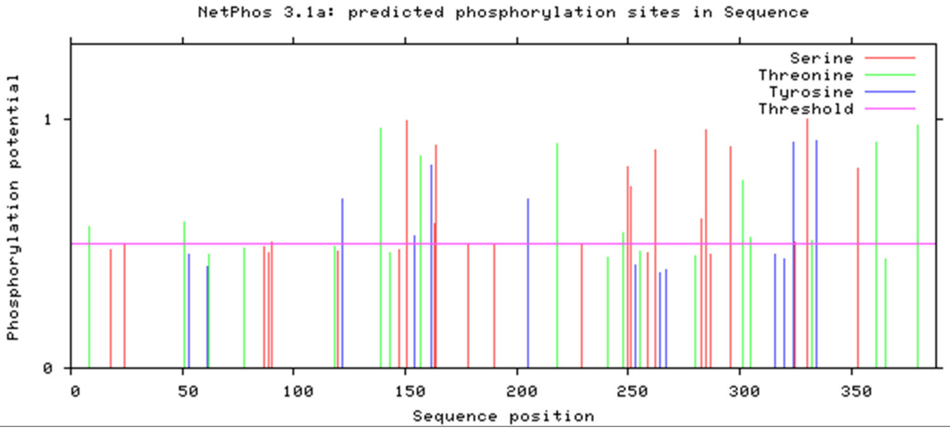	%1 ....T.. # 50<br>%1 T......................................S.......... # 100<br>%1 .....................Y................T........... # 150<br>%1 S..Y..T....YSS.................................... # 200<br>%1 ....Y............T.............................T.S # 250<br>%1 S..........S....................S.S..........S.... # 300<br>%1 T...T..................YS....S.T.Y................ # 350<br>%1 ..S.......T..................T........
**I3LK18**	*PKD2L2*	Ser561	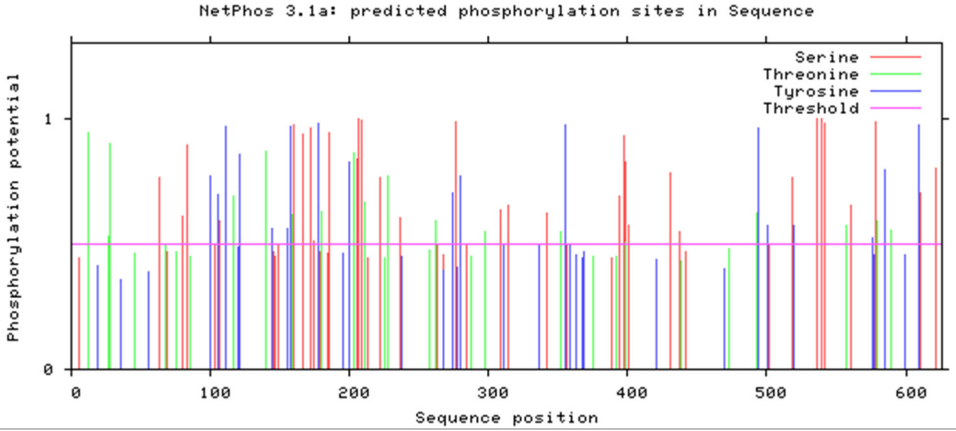	%1 ...........T..............TT...................... # 50<br>%1 ............S................S..S................Y # 100<br>%1 ....Y.S...Y....TS...Y..................T...Y...... # 150<br>%1 ....Y..YTS.....S.....S.S...Y.T....S..............Y # 200<br>%1 ..T.S.S.S.T..........S....ST.......S.............. # 250<br>%1 ...........T...........Y.S...Y................TT.. # 300<br>%1 ........S....S...........................S........ # 350<br>%1 .T..Y......................................S..SST. # 400<br>%1 S............................S......S............. # 450<br>%1 ....TY...... # 500<br>%1 Y................SY................S..S..S........ # 550<br>%1 ......T...S..............Y.ST.....Y...T........... # 600<br>%1 ........Y.S..........S....
**A0A8W4FCQ2**	*SPMI*	Tyr127; Tyr106; Thr27; Thr28; Thr48; Thr49	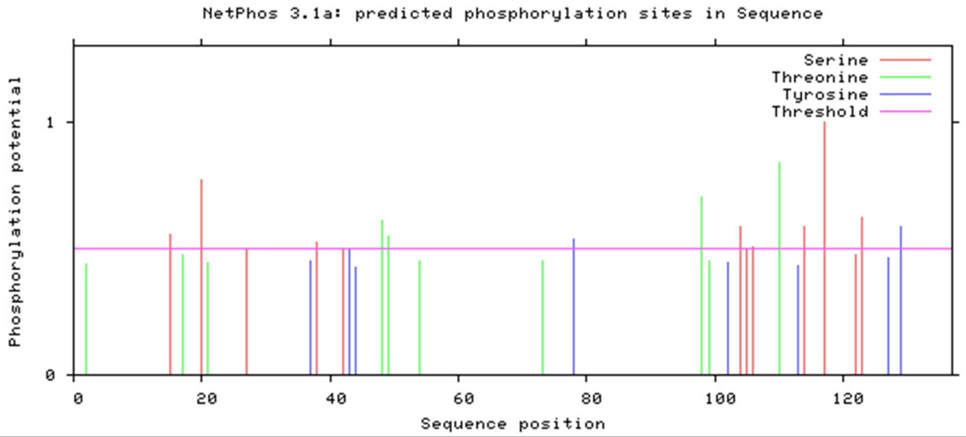	%1 ..............S....S.................S...S.....TT. # 50<br>%1 ...........................Y...................T.. # 100<br>%1 ...S.S...T...S..S.....S.....Y........
**D5K8A9**	*SPACA1*	Ser256; Ser262; Ser266; Ser278; Thr280; Tyr269-	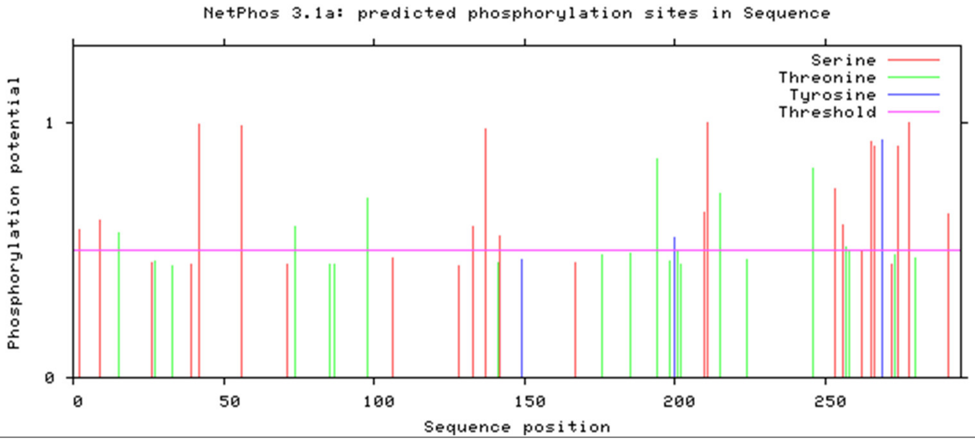	%1 .S......S.....T..........................S........ # 50<br>%1 .....S.................T.......................T.. # 100<br>%1 ................................S...S....S........ # 150<br>%1 ....T.....Y # 200<br>%1 .........SS...T..............................T.... # 250<br>%1 ..S..ST.......SS..Y....S...S............S....

**A0A4X1TEC5**	*SPACA3*	-	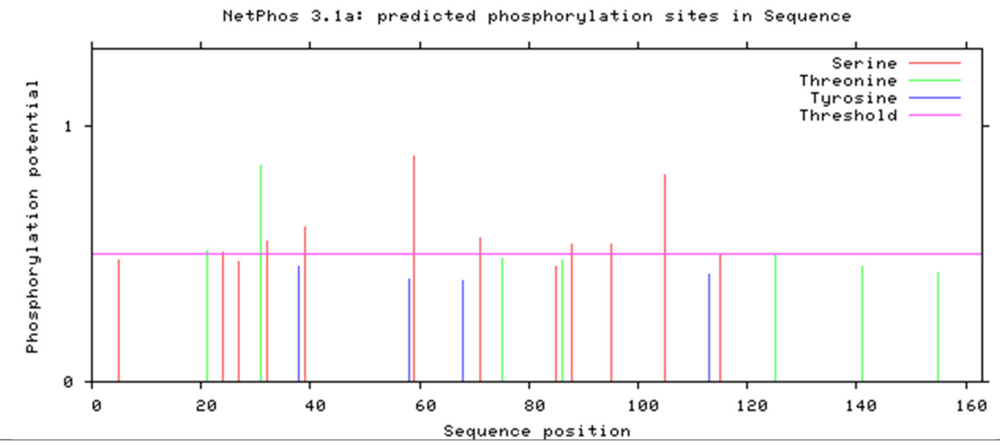	%1 ....................T..S......TS......S........... # 50<br>%1 ........S...........S................S......S..... # 100<br>%1 ....S... # 150<br>%1 ....
**Q4R0H8**	*AWN*	-	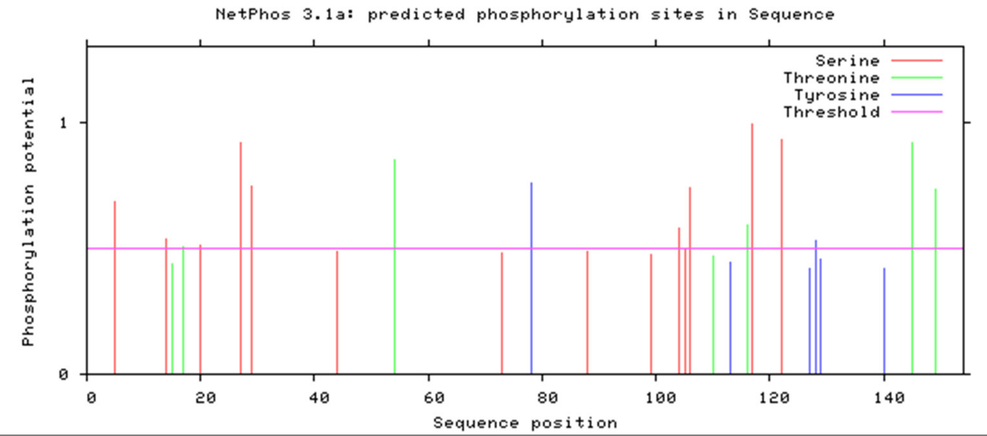	%1 ....S........S.....S.................S...ST.....T. # 50<br>%1 ...T.......................Y.................S.... # 100<br>%1 T..S.....S.........S.SS...Y.....
**A0A4X1T5L6**	*SPESP1*	-	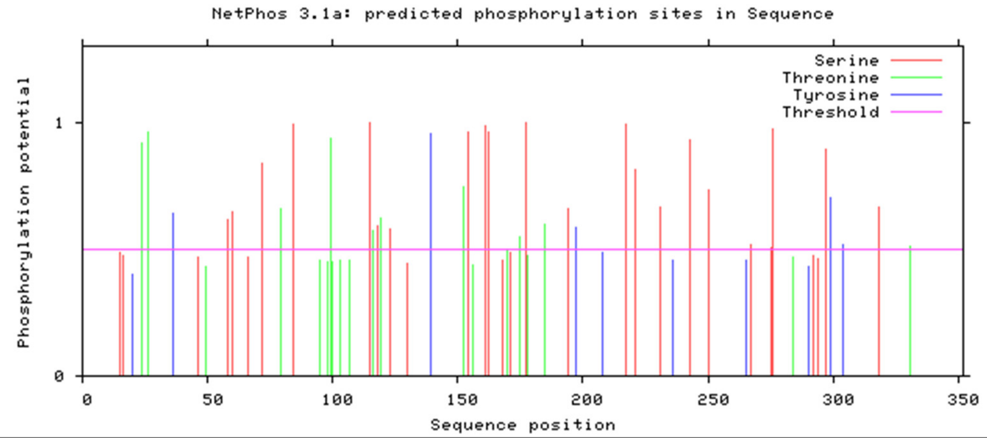	%1 .......................T.T.........Y.............. # 50<br>%1 .......S.S...........S......T....S..............T. # 100<br>%1 ..............ST.ST...S...............Y........... # 150<br>%1 .T.S......SS............T.S.......T........S..Y... # 200<br>%1 ................S...S.........S...........S......S # 250<br>%1 ................S.......SS....................S.Y. # 300<br>%1 ...Y.............S............T................... # 350<br>%1 ..
**A0A5G2QGK1**	*TUBB4B*	-	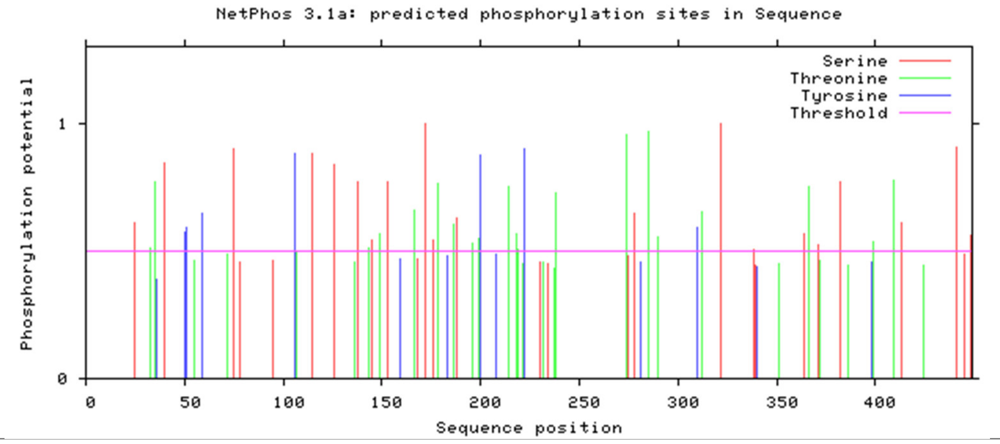	%1 ........................S.......T.T....S.........Y # 50<br>%1 Y.......Y...............S......................... # 100<br>%1 .....Y........S..........S...........S....T.S...T. # 150<br>%1 ..S............T.....S...S.T.......T.S.......T..TY # 200<br>%1 .............T...TT..Y...............T............ # 250<br>%1 .......................T...S......T....T.......... # 300<br>%1 .........Y.T.........S...............S............ # 350<br>%1 .............S.T....S..........S................T. # 400<br>%1 ........T...S...........................S......S.

**A0A287BCE6**	*FCGBP*	-	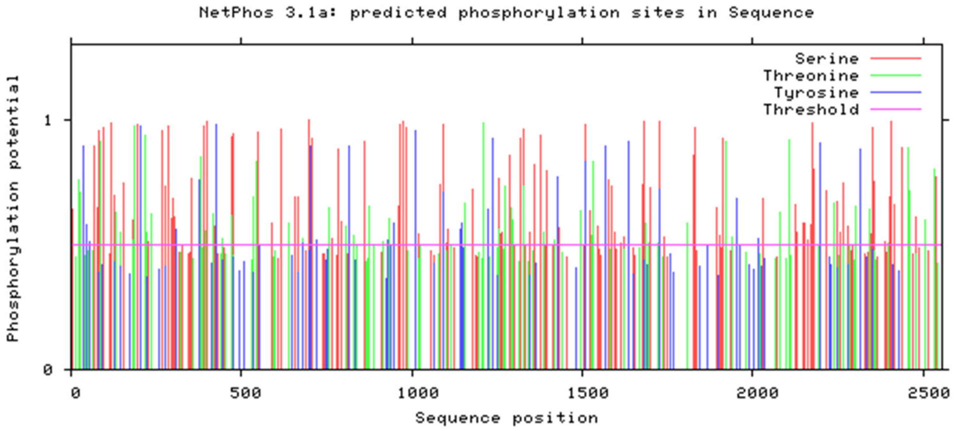	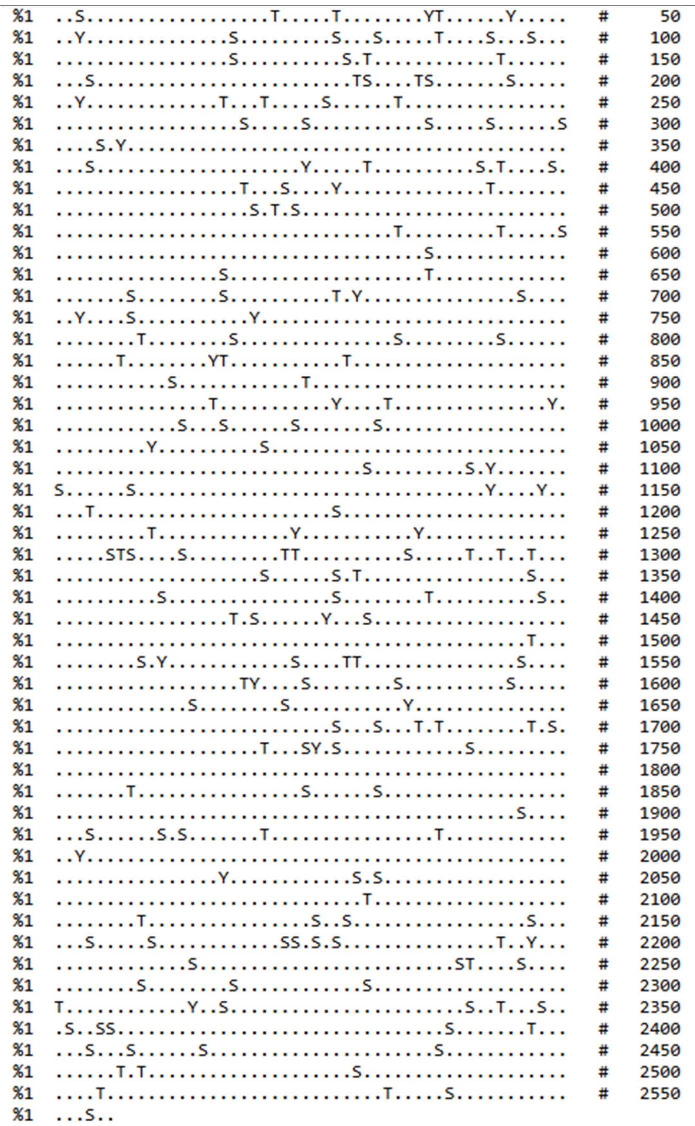
**C8C4M8**	*ZPBP2*	-	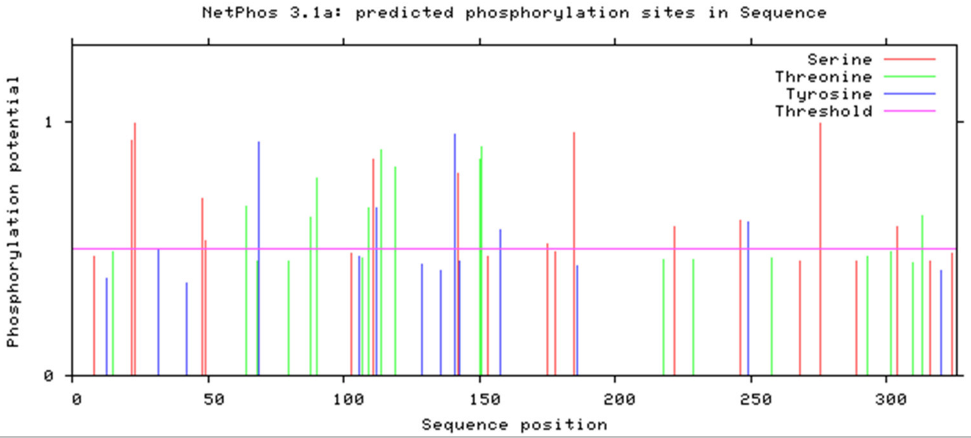	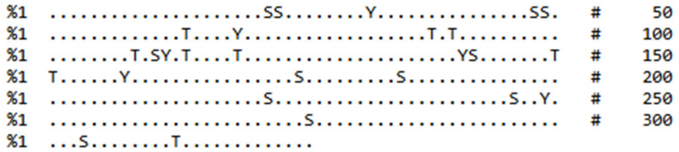
**Q29108**	*ZPBP*	Ser52	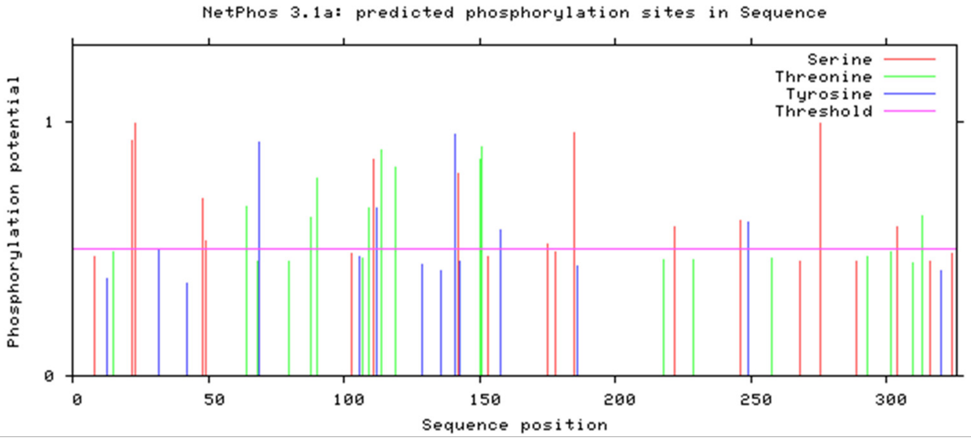	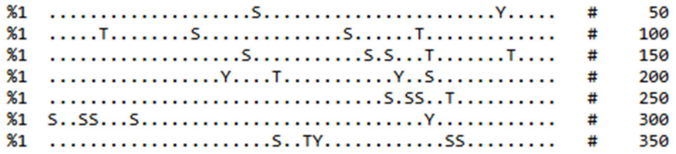

A0A5G2QZP6 *ZAN* -

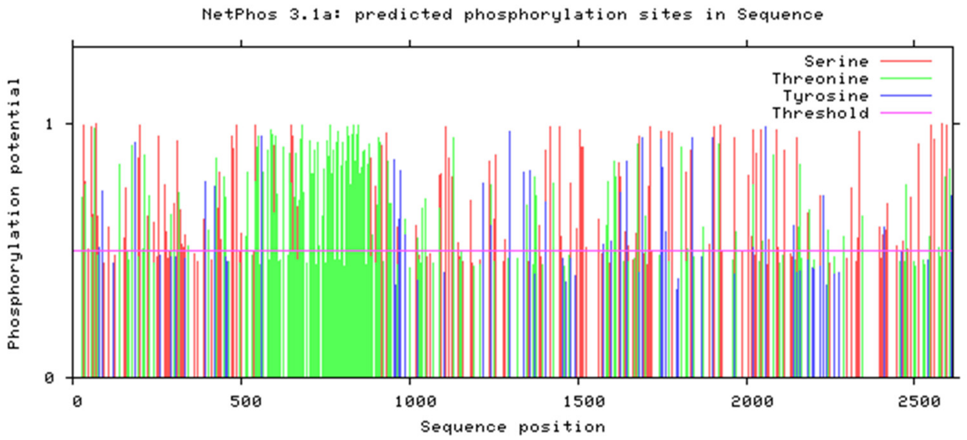


```
%1  ..........................T....S...S..T.......S...   #    50
%1  ...T..SS.......TS..SS..S......Y......Y............   #   100
%1  .....S..............................TS.S..........   #   150
%1  .S...S.S..................T......ST.Y........S....   #   200
%1  S........T....T.......S...T.T.........S...........   #   250
%1  ..S...................S...S...............T.....S.   #   300
%1  .T.........S....T..S.....S....S....S......T.......   #   350
%1  ......................................S....Y......   #   400
%1  .T.................Y....T...S....S....S...........   #   450
%1  T.T.................S...S..SS........S...........S   #   500
%1  ...............T.T......................TS.ST.....   #   550
%1  .T.........Y...T.............T......T......T..ST..   #   600
%1  T...T....................T...............T....S.T.   #   650
%1  .S...T......T..S...T......T..T.T.T..TT............   #   700
%1  ....T..TT..T..TT..T......T......T......T......T...   #   750
%1  ...T......T..TTT.T..TT..T......T...S..T......T....   #   800
%1  ..T......T..T.T.T..T.T.T..T.T.T..T.T.T..T.T.T..T.T   #   850
%1  .T..TT..T..T......T......T...TT...S.....T........S   #   900
%1  ....T.T...T...T...S...........SS.....T....T.T.....   #   950
%1  ...Y...........S.Y....Y..............TY...........   #  1000
%1  .................S..S....T.............T.......T..   #  1050
%1  .......................................ST.S.......   #  1100
%1  ......SS........S.........SS..TS............S.....   #  1150
%1  ................................SS................   #  1200
%1  ..................YT.................S.YT.........   #  1250
%1  ..S..S..........................S.S..........Y....   #  1300
%1  .......................................Y........T.   #  1350
%1  T......Y............T.T...T......S................   #  1400
%1  ....SY.............S.......T.................S..S.   #  1450
%1  ...........................S................S.....   #  1500
%1  .......SSS.....S......S...........................   #  1550
%1  ..........S................Y....T.....T.......Y...   #  1600
%1  ........T....T.......S.SSYS...........S.....Y..S..   #  1650
%1  ......................S.....T...S........YT.......   #  1700
%1  .T.......S....S.............................S.Y.S.   #  1750
%1  ..SY........Y.......S.........S...................   #  1800
%1  ........T.............S....T........S...Y.........   #  1850
%1  .....................T..................S.........   #  1900
%1  Y..S................T...S.........................   #  1950
%1  .............S......T.............................   #  2000
%1  .......SS......S..S.YT........S..............ST...   #  2050
%1  .......Y..........S.........T.........SS..S.......   #  2100
%1  .............S...........................Y......S.   #  2150
%1  ......T.T.........................S.T.............   #  2200
%1  ..................S.......Y...T.........T.........   #  2250
%1  .......................................T..........   #  2300
%1  ...........S..................S..S.........T......   #  2350
%1  ............................................S.....   #  2400
%1  .....S..Y....S......S..........................S..   #  2450
%1  ...............S......S...T..S.........S..........   #  2500
%1  .........S.................................T......   #  2550
%1  S.....S............T..........S..........T.S......   #  2600
%1  ..T.......Y...
```

Supplementary Table S2: Proteins for which phosphorylation was detected using MS, and their specific phosphorylation sites.

Uniprot ID	Protein names	Gene names	Amino acid	Positions within proteins
**A0A286ZIE8**	ADP/ATP translocase	*SLC25A4*	S	2
			S	7
**A0A286ZIV7**	LRRC37A/B like protein 1 C-terminal domain-containing protein	*LOC102158372*	S	766
**A0A286ZIX7**	Olfactory receptor	*LOC100155398*	S	75
			S	76
			T	77
			Y	83
**A0A286ZJL6**	MAPK/MAK/MRK overlapping kinase	*MOK*	S	322
			T	323
**A0A286ZJP9**	Neurabin-1	*PPP1R9A*	S	880
			T	875
**A0A286ZJV5**	FOS like 2, AP-1 transcription factor subunit	*FOSL2*	T	105
			T	108
**A0A286ZJV8**	Krev interaction trapped protein 1	*KRIT1*	S	236
			Y	230
**A0A286ZKB1**	Carboxylic ester hydrolase	*CEL*	Y	439
**A0A286ZLG7**	Small ribosomal subunit protein eS17		T	6
**A0A286ZMM8**	Tyrosine-protein kinase	*LYN*	S	248
			T	249
**A0A286ZMN5**	Inositol hexakisphosphate and diphosphoinositol-pentakisphosphate kinase	*PPIP5K2*	S	473
			Y	469
**A0A286ZMT6**	Tripartite motif-containing protein 29	*TRIM29*	S	67
			S	68
**A0A286ZNZ5**	Uncharacterized protein		S	302
			S	304
**A0A286ZPQ4**	DENN domain containing 4C	*DENND4C*	S	1114
**A0A286ZQE3**	Calmodulin-binding transcription activator 1	*CAMTA1*	Y	1573
			Y	1576
**A0A286ZQY1**	Dynein axonemal heavy chain 5	*DNAH5*	S	3023
			S	3027
			T	3026
**A0A286ZRS0**	Glutathione synthetase	*GSS*	T	438
**A0A286ZS33**	Breast cancer type 1 susceptibility protein homolog	*BRCA1*	S	4
**A0A286ZSG9**	Protein interacting with cyclin A1	*PROCA1*	S	383
			S	387
			S	392
**A0A286ZSQ8**	Beta-galactoside alpha-2,6-sialyltransferase 1	*ST6GAL1*	S	118
**A0A286ZT62**	Relaxin receptor 1	*RXFP1*	S	462
			Y	459
**A0A286ZVI6**	SAM and SH3 domain-containing protein 1	*SASH1*	S	1156
			T	1160
**A0A286ZWH7**	A-kinase anchoring protein 4	*AKAP4*	S	348
			S	232
			S	328
			S	136

			S	140
**A0A286ZWM0**	5'-nucleotidase	*NT5C3B*	T	202
			Y	203
**A0A286ZWQ5**	Coiled-coil domain containing 106	*CCDC106*	S	74
**A0A286ZWS2**	SH3 and PX domain-containing protein 2A	*SH3PXD2A*	S	1002
			T	1004
			T	1006
**A0A286ZXJ1**	Conserved oligomeric Golgi complex subunit 5	*COG5*	S	50
			T	45
			Y	46
**A0A286ZXL7**	PAX-interacting protein 1	*PAXIP1*	S	349
**A0A286ZYG3**	Brother of CDO	*BOC*	S	117
			T	121
			T	123
			Y	104
**A0A286ZYX7**	Bromodomain-containing protein 2	*BRD4*	S	601
**A0A286ZZ17**	Partner and localizer of BRCA2	*PALB2*	T	502
			T	504
**A0A286ZZD9;F1S8J6**	RAB2A, member RAS onco family;RAB2B, member RAS onco family	*RAB2A;RAB2B*	S	67;67
			S	70;70
**A0A287A0G8**	Endogenous retrovirus group K member 25 Pol		T	359
			Y	353
**A0A287A0K2**	Large ribosomal subunit protein mL49	*MRPL49*	T	135
			T	138
			T	144
			T	146
**A0A287A0S8**	Olfactory receptor	*LOC100513421*	T	132
			Y	122
			Y	131
**A0A287A450**	ubiquitinyl hydrolase 1	*USP31*	S	1195
			S	1200
			S	1203
			S	1205
**A0A287A4M4**	DNA helicase	*CHD2*	S	1695
**A0A287A580**	Cordon-bleu WH2 repeat protein like 1	*COBLL1*	S	251
			T	250
			T	253
**A0A287A8F1**	Filamin A interacting protein 1	*FILIP1*	S	85
**A0A287A9U5**	Protocadherin gamma subfamily B, 1	*PCDHGB1*	S	361
			S	363
			T	362
**A0A287A9Y2**	Serine/threonine-protein kinase RIO1	*RIOK1*	T	125
**A0A287AC03**	PAN2-PAN3 deadenylation complex catalytic subunit PAN2	*PAN2*	T	724
**A0A287AC30**	PAK4-inhibitor INKA2	*INKA2*	S	138
			T	137
			T	139
**A0A287AE79**	Vacuolar protein sorting 13 homolog D	*VPS13D*	S	1714
**A0A287AEA5**	Aldehyde dehydrogenase 16 family member A1	*ALDH16A1*	S	7
			T	5

**A0A287AEG1**	Microtubule actin crosslinking factor 1	*MACF1*	Y	494
			Y	495
**A0A287AFM0**	B-TFIID TATA-box binding protein associated factor 1	*BTAF1*	S	647
			S	649
			T	638
**A0A287AFN9**	Acrosin	*ACR*	S	56
**A0A287AFW5**	Transmembrane protease serine	*TMPRSS11E*	S	84
**A0A287AGC1**	G-protein coupled receptors family 1 profile domain-containing protein	*LOC100622639*	Y	232
**A0A287AH35**	SAM domain-containing protein SAMSN-1	*SAMSN1*	S	7
**A0A287AHW4**	Transient receptor potential cation channel subfamily A member 1	*TRPA1*	T	671
**A0A287AHY5**	Ribonucleoprotein PTB-binding 1	*RAVER2*	T	45
			T	49
**A0A287AHZ3**	Xin actin binding repeat containing 2	*XIRP2*	S	2623
**A0A287AIG0**	DNA repair protein REV1	*REV1*	S	789
			T	780
			T	782
			T	787
**A0A287AIU9**	Multiple C2 and transmembrane domain-containing protein 2	*MCTP2*	Y	621
**A0A287AK36**	Small integral membrane protein 20	*SMIM20*	S	82
**A0A287ANI1**	Regulator of G protein signaling like 1	*RGSL1*	T	1073
**A0A287ANV0**	F-BAR domain only protein 2	*FCHO2*	S	410
			T	408
**A0A287APA5**	Protein 4.1	*EPB41L3*	T	480
			T	481
**A0A287AQ20**	Complement factor I	*CFI*	S	400
			T	399
			T	402
**A0A287AQA5**	Dermatan sulfate epimerase like	*DSEL*	S	942
			S	943
**A0A287AQJ6;I3L8Z8**	LRRC37A/B like protein 1 C-terminal domain-containing protein	*LOC110255209;LOC100514*	S	763;754
			S	764;755
**A0A287AQK7;O02705**	Heat shock protein 90 alpha family class A member 1;Heat shock protein HSP 90-alpha	*HSP90AA1*	S	260;263
			S	228;231
**A0A287AR35**	Testis-expressed protein 10	*TEX10*	S	64
			Y	69
**A0A287ARA7**	Proteasome adapter and scaffold protein ECM29	*ECPAS*	S	956
			S	963
			S	981
**A0A287ARB4**	Myosin VC	*MYO5C*	S	931
			T	938
			Y	943
**A0A287ARW8**	Activating transcription factor 7 interacting protein 2	*ATF7IP2*	S	429
			S	432
			S	435
**A0A287ASB3**	Leucine rich repeats and IQ motif containing 1	*LRRIQ1*	T	1176
**A0A287ASK6**	Tyrosine-protein kinase receptor	*ROS1*	S	2220
**A0A287ATK3**	Bridge-like lipid transfer protein family member 1	*BLTP1*	Y	3286
			Y	3290
**A0A287AUX3**	Tyrosine-protein kinase	*ABL2*	S	1043

			S	1044
			S	1046
			T	1042
**A0A287AVR4**	Tyrosine 3-monooxygenase	*TH*	S	92
			S	94
			T	84
			T	88
**A0A287AVR8**	Zinc finger protein 677		S	342
			T	352
**A0A287AVZ9**	NACHT and WD repeat domain-containing protein 2	*NWD2*	T	8
**A0A287AWB1**	Voltage-dependent L-type calcium channel subunit alpha	*CACNA1F*	T	688
**A0A287AWH7**	SEC24 homolog A, COPII coat complex component	*SEC24A*	S	654
			Y	664
**A0A287AXD6**	WD repeat and FYVE domain-containing protein 1	*WDFY1*	S	190
			T	188
**A0A287AXW1**	Coiled-coil domain containing 121	*CCDC121*	T	22
**A0A287AY65**	Zinc finger protein 546	*ZNF546*	S	510
			S	515
**A0A287AYC9**	Dynein axonemal intermediate chain 3	*DNAI3*	S	472
			S	473
**A0A287AYP3**	COMM domain-containing protein 1	*COMMD1*	S	180
			S	182
**A0A287AZP6**	DNA excision repair protein ERCC-6-like 2	*ERCC6L2*	T	314
**A0A287B0A0**	Carotenoid-cleaving dioxygenase, mitochondrial	*BCO2*	S	298
**A0A287B0E7**	RNA polymerase II-associated protein 3	*RPAP3*	Y	326
**A0A287B0V4**	Ninein	*NIN*	S	1759
**A0A287B1C5**	Adenomatous polyposis coli protein	*APC*	T	1440
			T	1451
**A0A287B1Z4**	NHS like 2	*NHSL2*	S	276
			T	277
**A0A287B280**	DNA repair protein XRCC2	*XRCC2*	S	203
			T	194
			T	195
			T	202
**A0A287B2G1**	Tetratricopeptide repeat, ankyrin repeat and coiled-coil containing 2	*TANC2*	S	252
			S	254
**A0A287B2G4**	Stalled ribosome sensor GCN1	*GCN1*	S	8
			T	10
**A0A287B3D4**	Regulator of telomere elongation helicase 1	*RTEL1*	S	42
			T	44
			T	46
**A0A287B4X8**	Chromosome 6 C19orf81 homolog	*C6H19orf81*	T	144
**A0A287B580**	Protein regulator of cytokinesis 1	*PRC1*	S	195
			T	194
**A0A287B5E3**	E3 ubiquitin-protein ligase MIB2	*MIB2*	T	51
			T	60
			T	62
**A0A287B5X4**	Junctophilin-2	-	S	92
			S	93

			T	87
			T	98
**A0A287B7D2;I3LTX2**	Centrosomal protein of 70 kDa	*LOC110258506;CEP70*	S	220;220
**A0A287B8D1**	Adhesion G-protein coupled receptor G6	*ADGRG6*	S	1000
			Y	996
			Y	1001
**A0A287BA33**	Olfactory receptor	*LOC100522850*	T	11
**A0A287BAX5;F1RPV3**	T-box transcription factor T;T-box transcription factor 19	*TBXT;TBX19*	Y	210;209
**A0A287BCL7**	CFAP20 domain containing	*CFAP20DC*	S	45
			S	53
			T	56
**A0A287BD18**	Complement factor H	*CFH*	S	835
			Y	842
**A0A287BFN0**	Glutamate receptor	*GRIK4*	S	854
			T	850
			T	852
**A0A287BFY0;P00883**	Fructose-bisphosphate aldolase;Fructose-bisphosphate aldolase A	*ALDOA*	S	110;36
			S	113;39
**A0A287BFZ4**	SMC5-SMC6 complex localization factor 2	*SLF2*	S	236
			S	239
			Y	240
**A0A287BGF1**	Nesprin-2	*SYNE2*	S	4343
			T	4344
**A0A287BHB9**	Deubiquitinase MYSM1	*MYSM1*	T	504
**A0A287BIE4**	E3 ubiquitin-protein ligase NEDD4	*NEDD4*	T	569
			Y	570
**A0A287BIE7**	Cilia and flagella associated protein 65	*CFAP65*	S	345
			S	350
			Y	342
**A0A287BIL5**	Sad1 and UNC84 domain containing 2	*SUN2*	S	164
			S	166
			S	173
**A0A287BIW9**	Coiled-coil domain containing 18	*CCDC18*	S	1058
			S	1065
**A0A287BL02**	Protrudin	*ZFYVE27*	Y	50
**A0A287BLD5**	Phenazine biosynthesis like protein domain containing	*PBLD*	T	224
**A0A287BLF6**	Sushi domain-containing protein		T	1278
**A0A287BLM0**	Sprouty related EVH1 domain containing 1	*SPRED1*	S	216
**A0A287BLW7**	Adhesion G-protein coupled receptor G1	*ADGRG1*	S	219
			S	229
			T	228
**A0A287BNF0**	ATP binding cassette subfamily A member 12	*ABCA12*	S	702
			T	704
**A0A287BNZ6**	Disintegrin and metalloproteinase domain-containing protein 20-like	*LOC100525589*	S	742
			T	740
**A0A287BRX6**	DNA polymerase theta	*POLQ*	S	353
			S	355
			S	359
**A0A287BS45**	Junction mediating and regulatory protein, p53 cofactor	*JMY*	T	491

**A0A287BSL8**	DNA helicase	*CHD9*	Y	1491
**A0A480C1V5**	Tribbles homolog 1	*TRIB1*	S	139
			Y	127
			Y	134
**A0A480DIL6**	Coiled-coil domain containing 171	*CCDC171*	T	1158
**A0A480E3F5**	nucleoside diphosphate phosphatase	*ENTPD6*	S	398
			S	409
			S	410
			T	406
**A0A480EIA9**	PX domain-containing protein kinase-like protein	*PXK*	S	467
			S	470
			S	475
			S	479
**A0A480ELA5**	Transmembrane protein 178B	*TMEM178B*	T	154
			T	156
			Y	151
**A0A480I6I0**	Collagen alpha-1(XIV) chain	*COL14A1*	Y	95
**A0A480JM90**	alpha-1,2-Mannosidase	*MAN1A2*	T	304
			T	306
**A0A480JST9**	Elongator complex protein 1	*ELP1*	S	1283
			S	1284
**A0A480JZF5**	Disks large homolog 5	*DLG5*	S	773
			S	779
**A0A480K058**	palmitoyl-protein hydrolase	*DESI1*	Y	7
**A0A480LKZ8**	Zinc finger Ran-binding domain-containing protein 2	*ZRANB2*	T	55
**A0A480NQN9**	Outer dynein arm-docking complex subunit 2	*ODAD2*	S	479
			T	471
			T	473
**A0A480U871**	Electron transfer flavoprotein beta subunit lysine methyltransferase	*ETFBKMT*	S	140
**A0A480VJI0**	Gamma-tubulin complex component	*TUBGCP2*	S	72
			T	75
			Y	83
**A0A480WYZ2**	N-acetylneuraminate (7)9-O-acetyltransferase	*CASD1*	Y	74
**A0A480YVC5**	E2F transcription factor 1	*E2F1*	S	124
			S	129
			T	133
			T	134
**A0A481A0E5**	Mitochondrial transcription rescue factor 1	*MTRES1*	S	157
			S	161
**A0A4X1SN16**	Uncharacterized protein		S	44
**A0A4X1SWJ9**	26S proteasome non-ATPase regulatory subunit 12	*PSMD12*	S	222
			Y	206
			Y	207
**A0A4X1T6L4**	Monocarboxylate transporter 1	*SLC16A1*	S	468
**A0A4X1TGL9**	Vesicle-trafficking protein SEC22a	*SEC22A*	S	133
			T	134
**A0A4X1THK7**	Lysozyme like 4	*LYZL4*	S	125
**A0A4X1TI56**	Basal body-orientation factor 1	*BBOF1*	Y	72
**A0A4X1TLL5**	PHD finger protein 14	*PHF14*	S	303

			S	307
			S	318
**A0A4X1TNR4**	RUN and FYVE domain containing 2	*RUFY2*	T	435
**A0A4X1TT02**	Coiled-coil domain containing 69	*CCDC69*	S	149
			S	152
**A0A4X1TUN3;F1RN84**	Serine/threonine-protein kinase TAO3;non-specific serine/threonine protein kinase	*TAOK3;TAOK1*	Y	305;309
**A0A4X1TV12**	Olfactory receptor	*LOC100623897*	S	332
			S	335
**A0A4X1TZW7**	Enkurin	*ENKUR*	S	101
			S	58
			T	154
**A0A4X1U5C9**	FA complementation group I	*FANCI*	S	963
**A0A4X1U8I3**	TLC domain containing 2	*TLCD2*	S	234
			T	241
**A0A4X1UE61**	Sperm acrosome developmental regulator	*SPACDR*	S	131
**A0A4X1UE92**	Spectrin beta chain	*SPTB*	S	1236
			S	1240
**A0A4X1UI84**	Glucosamine-6-phosphate isomerase	*GNPDA2*	T	161
			T	166
**A0A4X1UN77**	Forkhead box protein M1	*FOXM1*	S	357
**A0A4X1UP83**	Basonuclin zinc finger protein 1	*BNC1*	T	303
			T	305
**A0A4X1V0Q0**	Zinc finger protein 106	*ZNF106*	S	471
			T	469
**A0A4X1V5G1**	F-box-like/WD repeat-containing protein TBL1XR1	*TBL1XR1*	T	337
			T	342
**A0A4X1VDQ8**	E3 ubiquitin-protein ligase TRIM69	*TRIM69*	T	288
**A0A4X1VG91**	Structural maintenance of chromosomes protein	*SMC4*	T	682
			T	686
			T	690
**A0A4X1VGA9;A0A5G2R8T3**	Myosin IF; Myosin IE	*MYO1F;MYO1E*	S	147;149
			T	159;161
**A0A4X1VI83**	Ankyrin repeat domain-containing protein 6	*ANKRD6*	T	352
**A0A4X1VLW1**	Coiled-coil domain-containing protein 86	*CCDC86*	S	270
**A0A4X1VT10**	Family with sequence similarity 221 member B	*FAM221B*	S	106
			S	112
**A0A4X1VTR6**	Palmitoyl-protein thioesterase 1	*PPT1*	S	115
			Y	109
**A0A4X1VTU2**	UDP-glucose 4-epimerase	*GALE*	S	276
			T	271
			T	273
**A0A4X1W3F8**	Alkaline phosphatase	*ALPL*	S	110
			T	113
			T	115
**A0A4X1W4K8**	Nuclear autoantigenic sperm protein	*NASP*	S	293
			Y	297
**A0A4X1WAV2**	ELAV-like protein	*ELAVL4*	S	253
			S	254
			S	259

**A0A4X1WBI1;CON__P05787**	Keratin, type II cytoskeletal 8	*KRT8*	S	400
			S	404
			S	408
			S	410
			S	412
			S	418
			T	413
			T	421
**A0A5G2Q9C7**	T-complex protein 1 subunit gamma	*CCT3*	S	214
**A0A5G2Q9P8**	Activating transcription factor 6	*ATF6*	S	175
			S	178
**A0A5G2QAH5**	Oxysterol-binding protein	*OSBP2*	S	65
			S	68
**A0A5G2QAN4**	Olfactory receptor	*LOC100525165*	T	13
**A0A5G2QCL1**	Complement C4A (Rodgers blood group)	*C4A*	S	313
**A0A5G2QCY2**	DnaJ heat shock protein family (Hsp40) member C5 gamma	*DNAJC5G*	T	17
**A0A5G2QET6**	Prolactin receptor	*PRLR*	S	161
			T	165
			Y	168
**A0A5G2QGP0**	Thrombospondin-2	*THBS2*	S	477
**A0A5G2QH93**	Uncharacterized protein	*LOC110261321*	S	112
			T	107
**A0A5G2QHE2**	Zinc finger protein 584	*ZNF584*	T	326
			Y	327
**A0A5G2QHR1**	Adhesion G protein-coupled receptor E2	*LOC100518417*	S	803
			S	805
			Y	808
**A0A5G2QI02**	IQ calmodulin-binding motif-containing protein 1	*IQCB1*	S	222
			S	225
			T	223
**A0A5G2QJ86**	mRNA cap guanine-N(7) methyltransferase	-	S	14
**A0A5G2QJI8**	Coiled-coil domain-containing protein 39	*CCDC39*	S	32
**A0A5G2QK43**	Golgi associated RAB2 interactor family member 5B	*GARIN5B*	S	282
			S	285
			T	22
			T	29
**A0A5G2QL50**	Zinc finger protein 862	*ZNF862*	S	571
			T	572
			T	574
**A0A5G2QLA4;O77636**	Disintegrin and metalloproteinase domain-containing protein 17	*ADAM17*	S	382
			T	387
			Y	379
**A0A5G2QNS8**	A-type voltage-gated potassium channel KCND1	*KCND1*	S	440
			T	442
			T	443
**A0A5G2QPC0**	WW and C2 domain containing 1	*WWC1*	S	203
			S	208
**A0A5G2QPT8**	Peptidase A1 domain-containing protein	*LOC100523770*	S	270
			Y	254

**A0A5G2QQD3**	Outer dense fiber protein 2	*ODF2*	S	124
**A0A5G2QSD9**	Uncharacterized protein	-	T	39
			T	41
			T	43
			T	54
			T	55
**A0A5G2QUG9**	Elongation factor 1-delta	*EEF1D*	S	36
			S	37
**A0A5G2QVG6**	Dipeptidyl peptidase like 10	*DPP10*	Y	277
			Y	283
			Y	285
**A0A5G2QVZ0**	Nitric oxide synthase-interacting protein	*NOSIP*	T	15
			Y	14
			Y	16
**A0A5G2QWA5**	Beta/gamma crystallin domain-containing protein 1 isoform X1	*CRYBG1*	S	143
**A0A5G2QWP0**	Plexin-D1	*PLXND1*	Y	1879
**A0A5G2QXD3**	IF rod domain-containing protein	*LOC100515166*	Y	153
**A0A5G2QZ86**	Ankyrin repeat and death domain containing 1A	*ANKDD1A*	T	321
			T	335
**A0A5G2R0K2**	Uncharacterized protein	-	T	90
			Y	93
**A0A5G2R1R6**	Acylamino-acid-releasing enzyme	*APEH*	S	187
**A0A5G2R3B0**	Uncharacterized protein	-	S	13
			S	16
**A0A5G2R3E7**	Tetratricopeptide repeat protein 29	*TTC29*	T	331
			Y	336
**A0A5G2R5J1**	Calcium-binding tyrosine phosphorylation-regulated protein	*CABYR*	S	350
			S	353
			S	356
			S	125
			S	132
			S	145
			T	348
			T	148
			T	149
**A0A5G2R7M3**	Inter-alpha-trypsin inhibitor heavy chain 4;Inter-alpha-trypsin inhibitor heavy chain H4	*ITIH4*	T	145
			T	67
**A0A5G2R815**	Phosphatidylinositol transfer protein alpha isoform	*PITPNA*	Y	102
**A0A5G2R9A4**	Formin homology 2 domain containing 3	*FHOD3*	T	1109
			T	1120
**A0A5G2R9R1**	NAC alpha domain containing	*NACAD*	S	1915
			T	1908
			T	1911
**A0A5G2RAW8**	Cullin family profile domain-containing protein	*LOC102159347*	Y	614
**A0A5G2RC17**	G-protein coupled receptors family 1 profile domain-containing protein	*LOC100519587*	S	132
			Y	131
**A0A5G2RG72**	HAT C-terminal dimerisation domain-containing protein	*ZBED10*	Y	984
**A0A5G2RGQ6**	Zinc finger protein 227	*ZNF226*	S	206
			S	207

			T	204
			T	211
**A0A5G2RIF5**	Tubulin-specific chaperone E	*TBCE*	S	7
			T	4
			T	6
			T	10
**A0A5G2RKN9**	Schlafen family member 11	*SLFN11*	Y	137
**A0A5G2RMU3**	L1 transposable element dsRBD-like domain-containing protein		S	76
			T	87
**A0A5G2RNH1**	cyclin-dependent kinase	*CDK17*	S	92
**A0A5K1U0J0**	Copper-transporting ATPase 1	*ATP7A*	S	1460
			S	1463
			S	1466
			S	1469
**A0A5K1U589**	E1 ubiquitin-activating enzyme	*UBA1*	S	833
**A0A5K1ULP5**	COP9 signalosome complex subunit 2	*COPS2*	Y	92
**A0A5K1UXV2**	DOP1 leucine zipper like protein A	*DOP1A*	S	735
			T	739
**A0A5K1VBH1;P29804**	Pyruvate dehydrogenase E1 component subunit alpha;Pyruvate dehydrogenase E1 component subunit alpha, somatic form, mitochondrial (Fragment)	*PDHA1;PD*	S	299;292
			S	301;294
			S	306;299
			T	292;285
**A0A8D0PFN3**	DNA repair protein RAD51 homolog 3	*RAD51C*	T	274
			T	278
			T	279
**A0A8D0TH99**	Membrane cofactor protein	*LOC100736569*	S	413
			S	422
			S	424
			T	421
**A0A8D0UY69**	Guanine nucleotide-binding protein subunit gamma	*GNG14*	S	9
			T	7
**A0A8D0UZ35**	PDZ domain-containing protein 11	*PDZD11*	S	13
			S	15
**A0A8D0X5U1**	Leucine-rich glioma-inactivated protein 1	*LGI1*	T	505
			Y	517
**A0A8D1CWK9**	Uncharacterized protein		T	35
			T	37
**A0A8D1F3W3**	Membrane-associated guanylate kinase, WW and PDZ domain-containing protein 2	*MAGI2*	S	426
			T	423
			T	427
			T	428
**A0A8D1HDH9**	Uncharacterized protein		T	15
**A0A8D1NY58**	Signal transducing adaptor family member 1	*STAP1*	S	159
**A0A8D1SDL0**	Uncharacterized protein	*ANKMY1*	S	920
			T	919
**A0A8W4F7B2**	HCLS1-binding protein 3	*HS1BP3*	S	9
**A0A8W4F8V4**	5'-nucleotidase, cytosolic IB	*NT5C1B*	S	213
			S	188
			S	173

			S	177
			S	185
			T	189
			T	180
**A0A8W4F987**	Ig-like domain-containing protein	*LOC110260307*	S	277
**A0A8W4F9A4**	Serine/threonine-protein kinase PLK	*PLK5*	Y	248
**A0A8W4FAN9**	DUF1725 domain-containing protein		T	26
**A0A8W4FBH2**	PKHD1 like 1	*PKHD1L1*	S	930
			S	934
			S	936
			Y	942
**A0A8W4FBI6**	Isobutyryl-CoA dehydrogenase, mitochondrial	*ACAD8*	S	30
			S	33
			S	38
			T	39
**A0A8W4FBJ9**	Bile acid-CoA:amino acid N-acyltransferase	*LOC110255172*	S	345
			Y	335
**A0A8W4FBQ5**	Huntingtin-interacting protein 1	*HIP1*	T	62
			T	67
**A0A8W4FBY2**	Uncharacterized protein		S	6
**A0A8W4FC68**	DUF1725 domain-containing protein	-	S	3
			S	4
			S	8
			S	23
			T	9
			T	18
			T	19
			T	24
**A0A8W4FCA6**	Reverse transcriptase domain-containing protein	-	Y	70
**A0A8W4FCQ2;P24020**	Seminal plasma sperm motility inhibitor/spermadhesin AQN-3-like protein;Carbohydrate-binding protein AQN-3	*SPMI*	Y	127;106
			T	48;27;48
			T	49;28;49
**A0A8W4FE41**	Golgin A4	*GOLGA4*	S	789
			S	526
			S	527
**A0A8W4FE64**	Dynein axonemal heavy chain 8	*DNAH8*	S	115
			S	118
**A0A8W4FEC6**	Olfactory receptor	-	Y	126;132
**A0A8W4FEL1**	Coiled-coil domain-containing protein	-	S	708
			S	711
			T	716
**A0A8W4FFY9**	SH3 domain-containing protein	-	S	3
			T	9
**A0A8W4FGN1**	KPRP N-terminal and LCE C-terminal like protein	*KPLCE*	T	51
			T	53
			T	63
			T	64
			Y	54
**A0A8W4FGW2**	Folliculin interacting protein 1	*FNIP1*	S	354

			S	361
**A0A8W4FGX9**	Uncharacterized protein	-	S	43
**A0A8W4FHN1**	Acetyl-CoA carboxylase 1	*ACACB*	Y	1132
**A0A8W4FHP0**	CDK5 regulatory subunit-associated protein 2	*CDK5RAP2*	S	1854
			T	1847
**A0A8W4FI55**	Fibrous sheath CABYR binding protein	*FSCB*	S	193
			S	125
			T	122
**A0A8W4FJ90**	Spt20-like SEP domain-containing protein	-	S	185
			T	184
**A0A8W4FJD2**	Unc-13 homolog A	*UNC13A*	S	949
			S	955
**A0A8W4FLJ2**	Ig-like domain-containing protein	-	S	93
			S	97
**A0A8W4FM87**	Endonuclease/exonuclease/phosphatase domain-containing protein	-	S	20
			Y	26
**A0A8W4FMH0**	Testis expressed 29	*TEX29*	S	102
**A0A8W4FMZ5**	Ubiquitin carboxyl-terminal hydrolase	*USP15*	T	160
**A0A8W4FNA2**	L1 transposable element RRM domain-containing protein		T	112
**A0A8W4FP74**	Interferon regulatory factor 5	*IRF5*	S	392
			T	388
**A0A8W4FPU7**	ATP synthase mitochondrial F1 complex assembly factor 1	*ATPAF1*	T	284
			T	290
**A0A8W4FQB9**	Olfactory receptor	-	S	310
			S	311
**A0A8W4FQL0**	H1.10 linker histone	*H1-10*	S	49
			T	55
			Y	48
**A1IU54**	Carboxypeptidase E	*CPE*	T	222
**B1PXG0**	Protein lin-28 homolog A	*LIN28A*	S	3
**B7TJ08**	RING-type E3 ubiquitin transferase	*RAD18*	T	328
**ENSBTAP00000038329**	-	-	S	15
			T	6
			T	14
**A0A4X1SVF9**	Periostin	*POSTN*	T	755;812
**CON__Q3MHN2**	-	-	S	238
**D5K8A2**	Mitochondria-eating protein	*SPATA18*	S	10
			S	13
			S	159
**D5K8A9**	Sperm acrosome membrane-associated protein 1	*SPACA1*	S	256
			S	262
			S	266
			S	272
			S	278
			T	257
			T	258
			T	280
			Y	269
**D5KJI2**	High mobility group protein HMG-I/HMG-Y	*HMGA1*	S	6

			S	8
			S	9
			S	14
**E2IKQ8**	Suppressor of cytokine signaling 4	*SOCS4*	S	9
**E5F1H9**	Suppressor of cytokine signaling 5	*SOCS5*	S	70
			S	72
			S	76
**F1RF02;A0A287BF95;A0A287AH55;A0A286ZKM7;F1RF01;F1S043**	Iroquois homeobox 6;Iroquois homeobox 2;Iroquois homeobox 1	*IRX6;IRX2;IRX1*	T	186;154;167;153;167;183
			T	189;157;170;156;170;186
			T	191;159;172;158;172;188
**F1RFL2**	Serine/threonine-protein kinase LMTK2	*LMTK2*	T	153
			Y	152
**F1RFN6**	DEAH-box helicase 37	*DHX37*	S	104
			T	102
			T	103
			T	108
**F1RFZ7**	BAI1 associated protein 3	*BAIAP3*	S	549
			T	547
**F1RGH8;I6YP72**	[tau protein] kinase;Glycogen synthase kinase-3 beta	*GSK3A;GSK3B*	Y	279;216
**F1RI72**	Golgin A3	*GOLGA3*	S	530
			T	526
**F1RIA2**	T-cell surface glycoprotein CD5	*CD5*	Y	54
**F1RIU9**	Coiled-coil-helix-coiled-coil-helix domain containing 2	*CHCHD2*	S	7
			S	17
**F1RJ11**	Family with sequence similarity 217 member B	*FAM217B*	T	191
**F1RJ15**	Synaptonemal complex protein 2	*SYCP2*	S	791
			S	794
			S	887
**F1RJ27**	Sperm associated antigen 5	*SPAG5*	S	706
			S	716
**F1RKC3**	RAS protein activator like 1	*RASAL1*	S	4
			S	5
**F1RKK0**	long-chain-fatty-acid--CoA ligase	*SLC27A6*	Y	325
**F1RL75**	Platelet-derived growth factor receptor beta	*PDGFRB*	S	731
			T	747
**F1RLR4**	DNA repair endonuclease XPF	*ERCC4*	S	312
			T	293
**F1RM87**	ADAM metallopeptidase domain 28	*ADAM28*	Y	243
**F1RMK9**	NLR family pyrin domain containing 8	*NLRP8*	S	227
**F1RNM3**	Protein phosphatase 1 regulatory subunit 35	*PPP1R35*	S	181
**F1RPB5**	DCN1-like protein	*DCUN1D3*	S	26
			S	28
			S	30
			Y	29
**F1RPB7**	Developmentally-regulated GTP-binding protein 1	*DRG1*	Y	245
**F1RPZ6**	Cysteine-rich secretory protein 2	*CRISP2*	S	213

			S	217
			T	205
**F1RQ56**	Testis expressed basic protein 1	*TSBP1*	S	404
			S	389
			T	396
**F1RQK4**	PH-interacting protein	*PHIP*	S	1274
**F1RQU2**	Heat shock protein 90 alpha family class B member 1	*HSP90AB1*	S	255
**F1RQZ6**	Meiosis specific with coiled-coil domain	*MEIOC*	S	420
**F1RR02**	Glial fibrillary acidic protein	*GFAP*	T	176
**F1RR24**	Spermatosis associated 32	*SPATA32*	S	129
			S	132
**F1RRS0**	Gasdermin-C	*LOC100151870*	S	3
			S	9
**F1RRS3**	Vesicle-fusing ATPase	*NSF*	S	717
**F1RRS9**	KAT8 regulatory NSL complex subunit 1	*KANSL1*	T	347
**F1RRW5**	Angiotensin-converting enzyme	*ACE*	S	1302
**F1RS42**	Serine/threonine-protein kinase Nek10	*NEK10*	S	1017
**F1RSD6**	Coilin	*COIL*	S	157
			S	159
**F1RSN2**	Glycosylphosphatidylinositol anchor attachment 1 protein	*GPAA1*	Y	122
**F1RT80**	Crossover junction endonuclease EME1	*EME1*	S	512
			Y	520
**F1RTE2**	Ubiquitin carboxyl-terminal hydrolase	*USP26*	Y	739
**F1RTT1**	Family with sequence similarity 135 member A	*FAM135A*	S	574
			T	578
			Y	575
**F1RTY6**	Peptidyl-prolyl cis-trans isomerase D	*PPID*	T	247
			Y	252
**F1RTY8**	DnaJ homolog subfamily C member 5B	*DNAJC5B*	S	14
			S	16
			T	15
**F1RX67**	UBX domain protein 8	*UBXN8*	T	126
			Y	120
**F1RXC2**	Carbonic anhydrase	*CA2*	S	2
**F1RXS2**	Synaptotagmin-like protein 5	*SYTL5*	S	252
			S	259
			S	262
			S	266
			S	269
			T	267
**F1RXT8**	Condensin-2 complex subunit H2	*NCAPH2*	S	106
			S	107
**F1RXY2**	Ubiquitin carboxyl-terminal hydrolase	*USP45*	Y	654
**F1RZM4**	Laminin subunit alpha-4	*LAMA4*	S	828
			S	844
**F1S027**	Telomerase reverse transcriptase	*TERT*	T	193
			T	200
**F1S098**	HECT-type E3 ubiquitin transferase	*HERC1*	S	237
			T	233

**F1S0C2**	2-amino-3-carboxymuconate-6-semialdehyde decarboxylase	*ACMSD*	S	106
			T	103
**F1S0H4**	tRNA-queuosine alpha-mannosyltransferase	*GTDC1*	S	355
			S	360
			S	363
**F1S0P7**	Outer dynein arm-docking complex subunit 4	*ODAD4*	S	268
**F1S1J7**	Zinc finger transcription factor Trps1	*TRPS1*	S	1194
			T	1189
			T	1198
**F1S1R1**	Cylicin 1	*CYLC1*	S	371
			S	376
			S	408
			S	413
			S	416
			S	297
			S	334
			S	339
			S	445
			S	450
			S	452
			S	518
			S	519
			S	523
			S	526
			S	527
			S	539
			S	540
			S	544
			S	547
			S	478
			S	482
			S	485
			S	490
			S	497
			T	409
			T	446
			T	486
**F1S1T6**	Exosome complex component RRP45	*EXOSC9*	S	270
			T	277
**F1S2M8**	Transmembrane protein 63C	*TMEM63C*	S	350
			T	346
**F1S3B0**	Dihydroorotate dehydrogenase (quinone), mitochondrial	*DHODH*	Y	37
**F1S3Q9**	Vanin 2	*VNN2*	S	73
			T	78
			Y	84
**F1S3S9**	Gamma-aminobutyric acid receptor subunit gamma-1	*GABRG1*	S	128
			T	129
**F1S564**	RNA 3'-terminal phosphate cyclase	*RTCA*	S	253
			S	261

			S	262
			T	252
**F1S5N5**	Cilia-and flagella-associated protein 58	*CFAP58*	S	843
**F1S675**	Voltage-dependent R-type calcium channel subunit alpha	*CACNA1E*	T	127
			Y	130
**F1S6T2**	AT-rich interactive domain-containing protein 3	*ARID3A*	T	261
**F1S6W6**	Polypeptide N-acetylgalactosaminyltransferase	*GALNT18*	S	127
			Y	125
**F1S700**	HIVEP zinc finger 2	*HIVEP2*	S	156
			S	157
			S	166
**F1S7N6**	cAMP responsive element binding protein 3 like 3	*CREB3L3*	S	308
			T	311
**F1S890**	Serine/threonine-protein kinase 35	*STK35*	S	300
			T	299
			Y	293
**F1S8J6**	RAB2B, member RAS onco family	*RAB2B*	S	200
			S	202
			S	205
			T	206
**F1S8N8**	Inactive phospholipase D5 isoform 1	*PLD5*	S	30
			S	32
			S	42
			S	43
			T	34
**F1SAH5**	UDP-N-acetylglucosamine--dolichyl-phosphate N-acetylglucosaminephosphotransferase	*DPAGT1*	S	319
			T	309
			T	311
**F1SB85**	Coiled-coil domain containing 146	*CCDC146*	S	156
**F1SB93**	Hepatocyte growth factor	*HGF*	S	404
			T	406
**F1SBA5**	Proteasome subunit alpha type	*PSMA8*	S	152
			S	166
			T	149
			T	154
**F1SBU6**	Cation channel sperm-associated auxiliary subunit delta	*CATSPERD*	Y	402
**F1SCV6**	Cilia and flagella associated protein 46	*CFAP46*	S	155
			S	157
**F1SDH8**	Phospholipid-transporting ATPase	*ATP8B3*	T	558
			T	567
**F1SED0**	Short/branched chain specific acyl-CoA dehydrogenase, mitochondrial	*ACADSB*	S	367
			T	380
			T	381
**F1SEU6**	LON peptidase N-terminal domain and RING finger protein 1	*LONRF1*	S	746
**F1SF51**	U3 small nucleolar RNA-associated protein 25 homolog	*UTP25*	T	15
			T	17
**F1SGC6**	NADH dehydrogenase [ubiquinone] 1 beta subcomplex subunit 5, mitochondrial	*NDUFB5*	S	11
			T	13
**F1SGF1**	26S proteasome non-ATPase regulatory subunit 6	*PSMD6*	T	430

**F1SGF3**	Sentan, cilia apical structure protein	*SNTN*	T	88
**F1SGI5**	IF rod domain-containing protein	*LOC100621351*	Y	72
**F1SGK9**	2',5'-phosphodiesterase 12	*PDE12*	S	76
			S	432
			S	433
			S	438
			S	442
			T	80
**F1SGR5**	Chromosome 2 C11orf91 homolog	*C2H11orf91*	S	90
**F1SGU1**	Olfactory receptor	*LOC100517094*	S	87
**F1SI15**	Pre-mRNA-processing factor 39	*PRPF39*	Y	536
**F1SIB6**	Low-density lipoprotein receptor-related protein 4	*LRP4*	T	670
**F1SID8**	MAP kinase-activating death domain protein	*MADD*	T	1369
**F1SJU0**	EF-hand domain-containing protein		S	79
**F1SK06**	WD repeat domain 93	*WDR93*	S	112
			T	121
			Y	115
**F1SLC7**	MAX dimerization protein 1	*MXD1*	S	145
			S	148
			S	151
			S	152
			T	146
			T	149
**F1SM62**	RIB43A domain with coiled-coils 2	*RIBC2*	S	100
**F1SMT6**	Endothelin converting enzyme like 1	*ECEL1*	S	542
			S	550
**F1SNB0**	Sushi domain containing 1	*SUSD1*	S	375
			T	370
			T	373
**F1SPI5**	FYVE, RhoGEF and PH domain containing 5	*FGD5*	Y	1165
**F1SPM2**	Rho GTPase activating protein 25	*ARHGAP25*	S	546
			S	553
**F1SPP5**	Semaphorin 3F	*SEMA3F*	Y	248
**F1SQF7**	Transcriptional adapter 3	*TADA3*	S	290
**F1SQN3**	Connector enhancer of kinase suppressor of Ras 2	*CNKSR2*	S	488
**F1SQU9**	FA complementation group B	*FANCB*	S	415
			S	417
**F1SQW2**	SUMO specific peptidase 5	*SENP5*	S	82
**F1SS24**	Fibronectin	*FN1*	T	214
**F1SS52**	Dynein axonemal heavy chain 9	*DNAH9*	S	1933
			S	1941
**F1SSJ1**	GTPase IMAP family member 8	*GIMAP8*	T	415
			Y	417
			Y	419
**F1SSL6**	Proteasome subunit alpha type	*PSMA3*	S	250
**F1SSS4**	Unc-80 homolog, NALCN channel complex subunit	*UNC80*	T	1352
**F1STE8**	Eukaryotic translation initiation factor 5B	*EIF5B*	S	221
**F1STR4**	Serine protease 23	*PRSS23*	Y	369
**F1STU5**	Transmembrane protein 126B	*TMEM126B*	S	85

			T	75
			T	77
**F1SU01**	Ubiquitin carboxyl-terminal hydrolase 48	*USP48*	T	577
**F1SUS3**	Spermatosis associated 21	*SPATA21*	S	646
			T	651
**F1SUT8**	Ephrin type-A receptor 2	*EPHA2*	S	481
**F2Z552**	RAB39B, member RAS onco family	*RAB39B*	T	129
			T	134
**I3L568**	Centrosomal protein 250	*CEP250*	S	931
			T	935
			T	939
**I3L5I4**	Structural maintenance of chromosomes flexible hinge domain-containing protein 1	*SMCHD1*	T	1958
			T	1960
**I3L601**	Cilia and flagella associated protein 92 (putative)	*CFAP92*	T	870
**I3L7Q5**	Ecotropic viral integration site 5 like	*EVI5L*	S	66
**I3L7Q8**	Anaphase-promoting complex subunit 7	*ANAPC7*	T	67
**I3L7S0**	Progesterone immunomodulatory binding factor 1	*PIBF1*	T	130
**I3L802**	CBFA2/RUNX1 partner transcriptional co-repressor 2	*CBFA2T2*	Y	381
**I3L9T2**	DNA ligase	*LIG3*	S	884
**I3LC61**	Cylicin 2	*CYLC2*	S	122
			S	128
			S	131
			S	135
			S	169
			S	105
			S	107
**I3LC88**	Low-density lipoprotein receptor-related protein 1B	*LRP1B*	S	4071
			T	4081
**I3LD14**	Receptor expression-enhancing protein	*REEP1*	S	138
			T	131
**I3LD34**	Membrane spanning 4-domains A14	*MS4A14*	S	444
			S	3
			S	4
			T	446
**I3LDB3**	Olfactory receptor	*LOC106507330*	S	3
**I3LDC5**	Galactose-3-O-sulfotransferase 3	*GAL3ST3*	Y	223
**I3LDH5**	Aldehyde oxidase 1	*AOX1*	S	531
**I3LDH9**	Uncharacterized protein	*CCDC162*	S	497
**I3LDS3**	Keratin, type I cytoskeletal 10	*KRT10*	S	6
			S	7
			S	8
			S	12
			S	13
			S	14
			S	16
**I3LDY5**	D-lactate dehydrogenase (cytochrome)	*LDHD*	S	131
			T	139
**I3LG01**	Rho guanine nucleotide exchange factor 38	*ARHGEF38*	S	217
**I3LG23**	Macrophage-stimulating protein receptor	*MST1R*	S	1133

			Y	1132
**I3LGD4**	Clathrin heavy chain	*CLTC*	T	173
**I3LI11**	Zinc finger protein 628	*ZNF628*	S	587
			Y	593
**I3LK18**	Polycystin-2-like protein 2	*PKD2L2*	S	561
**I3LL53**	Trimethylguanosine synthase	*TGS1*	Y	733
**I3LM88**	Helicase-like transcription factor	*HLTF*	T	641
**I3LMW7**	MAX dimerization protein 4	*MXD4*	S	114
**I3LMZ5**	Succinate receptor 1	*SUCNR1*	T	219
			T	220
**I3LN22**	Patched domain-containing protein 3	*PTCHD3*	S	75
**I3LN27**	PDZ domain containing 9	*PDZD9*	S	149
**I3LQS1**	Neuronal tyrosine phosphorylated phosphoinositide-3-kinase adaptor 1	*NYAP1*	Y	5
**I3LQY2**	DNA excision repair protein ERCC-5	*ERCC5*	S	259
			T	984
**I3LR59**	Family with sequence similarity 83 member C	*FAM83C*	T	731
			Y	733
**I3LRC2**	Zinc finger B-box domain containing	*ZBBX*	S	598
			S	602
			S	604
**I3LRP8**	Sulfotransferase	*SULT2B1*	S	289
			S	294
			T	285
**I3LS52**	Centrosomal protein of 76 kDa	*CEP76*	S	305
			Y	296
**I3LSJ7**	Ectopic P-granules 5 autophagy tethering factor	*EPG5*	S	897
			Y	884
			Y	890
**I3LT35**	Glutamate receptor	*GRIK1*	Y	167
			Y	168
**I3LUU8**	Sorting nexin-13	*SNX13*	S	953
**I3LVT2**	Testicular spindle-associated protein SHCBP1L	*SHCBP1L*	Y	320
**K7GKM6**	Repetin	*RPTN*	S	7
			T	10
**K7GKY0**	CD109 antigen	*CD109*	T	392
			T	394
**K7GT68**	Integrin alpha-6	*ITGA6*	S	585
			S	592
			T	593
**M3TYW5**	Glutamine-tRNA ligase	*QARS1*	T	305
**O02696**	Phosphoinositide 3-kinase regulatory subunit 5	*PIK3R5*	S	428
			S	430
			T	429
**P02666**	Beta-casein	*CSN2*	S	35
			S	50
**P02768**	Albumin	*ALB*	S	443
			S	442
			T	443
			T	444

**P02769**	Albumin	*ALB*	S	296;296
			Y	184;184
**P04264**	Keratin, type II cytoskeletal 1	*KRT1*	S	21
**P05207**	cAMP-dependent protein kinase type II-alpha regulatory subunit (Fragment)	*PRKAR2A*	S	41
**P07802**	cAMP-dependent protein kinase type I-alpha regulatory subunit	*PRKAR1A*	S	5
			S	8
**P14082**	Neurotrophic factor BDNF precursor form	*BDNF*	S	218
			T	215
			T	216
			T	224
**P36887**	cAMP-dependent protein kinase catalytic subunit alpha	*PRKACA*	S	339;339
			T	209
			T	209
**P67985**	Large ribosomal subunit protein eL22	*RPL22*	S	66
**Q06A98**	Serine/arginine-rich splicing factor 2	*SRSF2*	T	51
			Y	44
**Q1W674**	Hexokinase-2	*HK2*	S	788
			S	792
			T	784
**Q29077**	Outer dense fiber protein 1	*ODF1*	S	5
			S	167
**Q29106;A0A5G2QHK1**	Regulatory solute carrier protein family 1 member 1;Regulator of solute carriers 1	*RSC1A1*	S	370;775
			S	371;776
			S	379;784
			T	378;783
**Q29108**	Zona pellucida-binding protein 1	*ZPBP*	S	52
**Q29244;A0A287B644**	Polymeric immunoglobulin receptor (Fragment);Polymeric immunoglobulin receptor	*PIGR*	T	45;274
			T	52;281
**Q2HY40**	Thioredoxin-interacting protein	*TXNIP*	T	227
			T	231
**Q2IA00**	Sperm flagellar protein 2	*SPEF2*	S	486
**Q6PQZ2**	Cystic fibrosis transmembrane conductance regulator	*CFTR*	S	956
			S	963
			T	964
**Q7PCJ9**	Thialysine N-epsilon-acetyltransferase	*SAT2*	S	3
**Q8MI02**	Hyaluronidase	*SPAM1*	T	360
			T	361
			T	370
			Y	365
**Q9GL01**	Dolichyl-diphosphooligosaccharide--protein glycosyltransferase subunit 2	*RPN2*	S	242
			S	242

Key: S – serine, T – threonine, Y – tyrosine

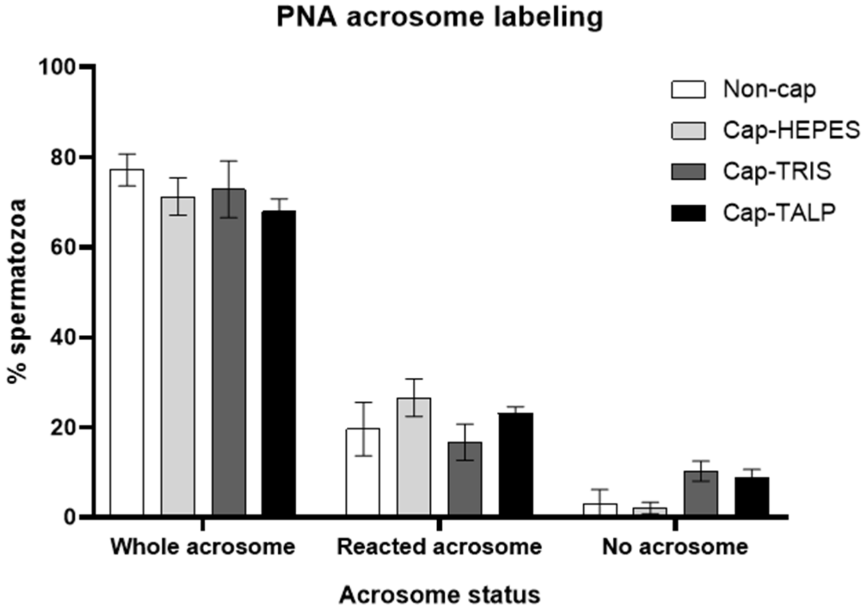

	Non-cap	Cap-HEPES	Cap-TRIS	Cap-TALP
**Whole acrosome**	77.23 ± 2.89 [a]	71.30 ± 3.35 [a]	72.91 ± 5.13 [a]	68.01 ± 2.30 [a]
**Reacted acrosome**	19.68 ± 4.84 [b]	26.68 ± 3.39 [b]	16.78 ± 3.29 [b]	23.18 ± 1.18 [b]
**No acrosome**	3.09 ± 2.52 [c]	2.03 ± 1.06 [c]	10.31 ± 1.85 [b]	8.82 ± 1.54 [c]

Supplementary Figure S1: PNA acrosome labeling of boar spermatozoa under different incubation conditions. The bar graph shows the proportion of spermatozoa classified according to acrosome status as whole acrosome, reacted acrosome, or no acrosome following peanut agglutinin (PNA) labeling in non-capacitated spermatozoa (Non-cap) and spermatozoa incubated in Hepes-, Tris-, or TALP-based capacitation media (Cap-HEPES, Cap-TRIS, Cap-TALP). Data are expressed as mean ± SEM (% of spermatozoa). Different superscript letters in the table indicate statistically significant differences ($p < 0.05$). Quantification was based on the evaluation of at least 200 spermatozoa per sample.

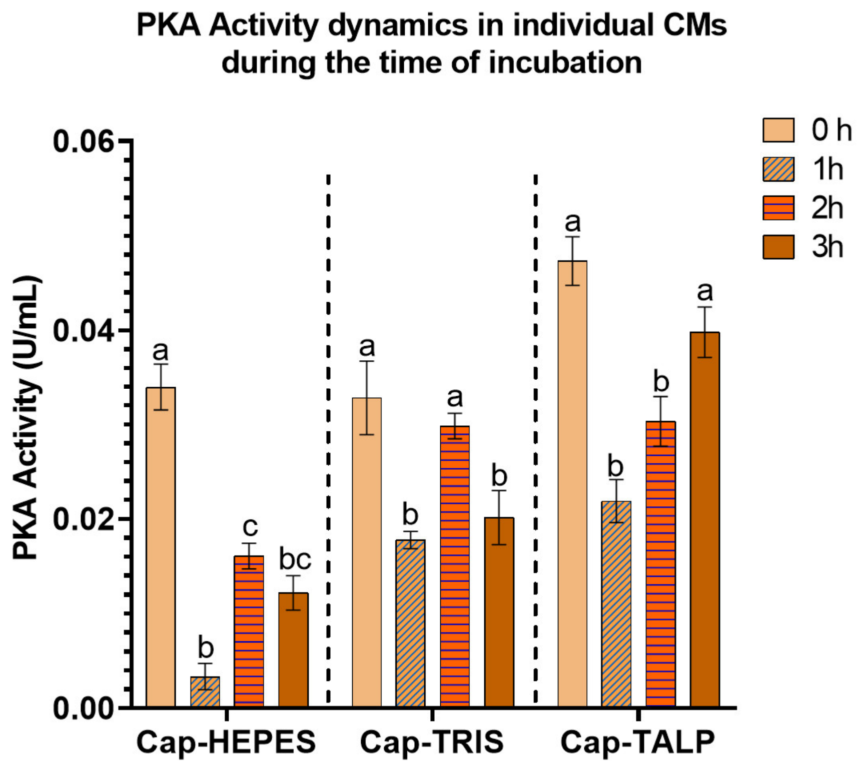

Supplementary Figure S2: Comparison of changes in PKA activity within each group (Cap-HEPES, Cap-TRIS, and Cap-TALP) during incubation. Error bars represent SEM; statistically significant differences ($p < 0.05$) in PKA activity are indicated by letters; $n = 4$.
